# Hijacking the MDM2 E3 Ligase with Novel BRD4‐Targeting Proteolysis‐Targeting Chimeras in Pancreatic Cancer Cells

**DOI:** 10.1002/cbic.202500133

**Published:** 2025-06-23

**Authors:** Mihaela P. Ficu, Dan Niculescu‐Duvaz, Mohammed Aljarah, Christopher S. Kershaw, Caroline J. Springer

**Affiliations:** ^1^ Drug Discovery Unit Cancer Research UK Manchester Institute University of Manchester Alderley Park Macclesfield SK10 4TG UK; ^2^ Oncodrug Ltd Alderley Park Macclesfield SK10 4TG UK

**Keywords:** BRD4, HDM2, MDM2, MIA PaCa‐2, pancreatic cancer, PROTACs, targeted protein degradation

## Abstract

The phenotypic effect induced by a Proteolysis‐Targeting Chimera (PROTAC) can depend on several factors, including the E3 ligase recruited. For the discovery of a first‐in‐class PROTAC for a target of interest, the E3 ligases commonly hijacked remain the Von Hippel‐Lindau (VHL) and Cereblon (CRBN) since potent and accessible ligands are readily available to recruit them. Mouse double minute 2 (MDM2) E3 ligase stands out because it regulates p53 levels to maintain cellular homeostasis. However, the synthesis of the most potent MDM2 ligands remains very complex. Herein, the discovery of novel MDM2‐recruiting PROTACs incorporating *rac*‐Nutlin‐3 as a ligand with an easier synthetic tractability is reported, further demonstrating its potential in this technology. The most promising degrader, PROTAC **3**, showed preferential degradation of the BRD4 short isoform (BRD4 S) and c‐Myc compared with MZ1, a validated VHL‐based PROTAC.

## Graphical abstract

The discovery of novel ligands recruiting less explored E3 ligases remains challenging.  Herein, the discovery of a novel Mouse double minute 2‐recruiting Proteolysis‐Targeting Chimera is reported, which shows preferential degradation of the Bromodomain‐containing protein 4 short isoform (BRD4 S) and c‐Myc compared with MZ1, a validated Von‐Hippel Lindau‐based PROTAC.

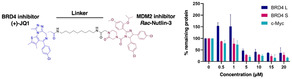

## 1 Introduction

Novel strategies and technologies in the oncology drug discovery field have been directed towards tackling the complexities of cancer progression, particularly to overcome and potentially prevent undesired side effects of existing first‐line cancer therapies.^[^ ^1^ ^]^ One such exciting technology that expanded rapidly over the past 20 years is the Targeted Protein Degradation (TPD) field, which encompasses molecules that hijack the cellular machinery to degrade proteins involved in disease progression.^[^ ^2^ ^]^ Its tremendous potential has been demonstrated by 30 degraders in clinical trials, one of them already used in a Phase III clinical trial.^[^ ^2^ ^]^

Protein degradation strategies can involve the recruitment of the component proteins from the Ubiquitin‐Proteasome System (UPS) such as an E3 ligase,^[^ ^3^ ^]^ an E2 enzyme,^[^ ^4^ ^]^ or even the proteasome.^[^ ^5^ ^]^ Examples include Proteolysis‐Targeting Chimeras (PROTACs),^[^ ^3^ ^]^ molecular glues,^[^ ^6^, ^7^ ^]^ or hydrophobic tagging.^[^ ^8^ ^]^ The Autophagy‐Lysosome pathway has also been hijacked with Autophagy‐Targeting Chimeras (AUTACs),^[^ ^9^ ^]^ Lysosome‐Targeting Chimeras (LYTACs),^[^ ^10^ ^]^ Autophagosome‐Tethering Compounds (ATTECs),^[^ ^11^ ^]^ and others.^[^ ^3^ ^]^

An effective PROTAC molecule needs to simultaneously bind to a protein of interest (POI) and an E3 ubiquitin ligase to induce the tagging of the POI for proteasomal destruction, through the formation of a productive ternary complex^[^ ^12^ ^]^ and efficient ubiquitination.^[^ ^13^ ^]^ PROTACs are heterobifunctional molecules incorporating ligands that bind to the POI and the E3 ligase and a linker between the two, attached to solvent‐exposed functional groups.^[^ ^3^ ^]^

With over 800 E3 ubiquitin ligases that dictate the substrate recognition and specificity of the UPS, having the tools to hijack any E3 ligase could provide the opportunity to discover tissue‐selective or cell type‐specific degraders.^[^ ^14^, ^15^ ^]^ Although there is active research leveraging chemoproteomics approaches, such as activity‐based protein profiling, to expand the E3 ligase toolbox,^[^ ^16^, ^17^ ^]^ the Von Hippel‐Lindau (VHL) and Cereblon (CRBN) remain the most commonly recruited E3 ligases for PROTAC development with well‐validated ligands.^[^ ^3^ ^]^

The major barriers that have slowed drug discovery efforts to target E3 ligases beyond VHL and CRBN are the lack of small molecule ligands,^[^ ^18^ ^]^ the structural heterogeneity of E3 ligases within the same family, their unknown mechanism of activation^[^ ^19^ ^]^ and the high costs to elucidate their biology and ligandability.^[^ ^20^ ^]^ As a result, to date only 14 E3 ligases have been used to hijack the UPS.^[^ ^18^ ^]^

There are several recent innovations that are facilitating the discovery of novel ligands to expand the E3 ligase toolbox in the TPD field.^[^ ^18^ ^]^ For example, cryo‐EM and artificial intelligence tools, such as AlphaFold, are used in structure‐guided ligand design.^[^ ^18^ ^]^ In addition, phenotypic and genomic screening strategies were used to discover novel ligands for E3 ligases, such as DCAF15, DDB1, and Met30.^[^ ^18^ ^]^ Other strategies include fragment‐based covalent screening, biophysical, and biochemical techniques, such as affinity selection mass spectrometry, DNA‐encoded libraries, docking‐based virtual high‐throughput screening, and protein microarrays.^[^ ^18^ ^]^

Mouse double minute 2 (MDM2) E3 ligase stands out because it regulates the tumor suppressor p53 by inducing its degradation to maintain cellular homeostasis.^[^ ^21^ ^]^ Dysregulation of p53 leads to a loss of its key function as a tumor suppressor, with p53 mutants gaining oncogenic properties.^[^ ^22^ ^]^ The majority of malignant tumors have p53 mutated or inactivated, and rescuing wild type p53 with novel drugs is a good strategy to prevent tumor progression.^[^ ^22^ ^]^

Since cancer cells rely on p53 inactivation for survival through MDM2 overexpression or by gaining p53 mutations, targeting the MDM2‐p53 protein–protein interaction has emerged as a therapeutic strategy to rescue wild type p53.^[^ ^23^ ^]^ The nine MDM2 inhibitors evaluated in clinical trials^[^ ^24^ ^]^ mimic three key amino acid residues in the p53 pocket recognized by MDM2, and hence contain several chiral centers with poor synthetic tractability for the extensive trial‐and‐error process necessary to discover a degrader.

**Figure 1:**
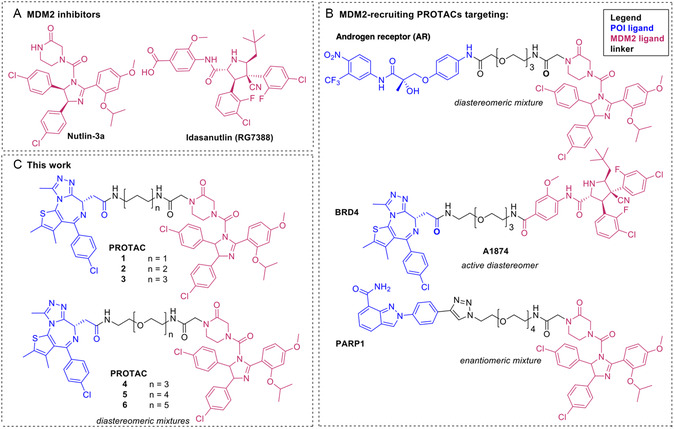
Chemical structures of: A) representative MDM2 inhibitors,^[^ ^26^, ^37^ ^]^ B) previously reported MDM2‐recruiting PROTACs,^[^ ^27^, ^28^, ^29^ ^]^ and C) the library of MDM2‐recruiting PROTACs described in this work.

A small number of MDM2‐based PROTACs have been developed by using either Nutlin‐3a (IC_50_ = 90 nM)^[^ ^25^ ^]^ or Idasanutlin (RG7388, IC_50_ = 6 nM)^[^ ^26^ ^]^ to degrade the androgen receptor (AR) in cervical carcinoma HeLa cells transiently expressing AR,^[^ ^27^ ^]^ bromodomain‐containing protein 4 (BRD4) in colorectal cancer cell line HCT116,^[^ ^28^ ^]^ poly (ADP‐ribose) polymerase 1 (PARP1) in breast cancer cell line MDA‐MB‐231,^[^ ^29^ ^]^ and MDM2 itself in non‐small cell lung cancer cell line A549 (**Figure** **1A,B**).^[^ ^30^ ^]^

The progress of the MDM2 inhibitors in the clinic has been slow due to a lack of efficacy, even with compounds such as Idasanutlin with very promising preclinical data, highlighting the need for a different strategy to rescue p53 in cancer.^[^ ^24^ ^]^

Given that MDA‐MB‐231 was the only cell line with mutant p53 status among the models used in the literature to test MDM2‐recruiting PROTACs, we aimed to expand the application of MDM2‐based degraders to include an *in vitro* pancreatic cancer model with mutant p53 status and to strengthen the evidence behind racemic Nutlin‐3 as an accessible ligand for the generation of PROTAC molecules.

## 2 Results and Discussion

A library of six *rac*‐Nutlin‐3‐based PROTACs with different linkers based on methylene units and polyethylene glycol (PEG) units was designed and synthesized to target BRD4, an extensively studied POI for proof of concept studies in the TPD field (**Figure** **1C**).^[^ ^31^ ^]^ BRD4 inhibition also sensitized the pancreatic cancer cell line MIA PaCa‐2 to gemcitabine, which is the first‐line treatment for pancreatic ductal adenocarcinoma.^[^ ^32^ ^]^ In addition, inhibitors with pan‐affinity against bromodomain and extra‐terminal domain (BET) proteins have shown efficacy in preclinical pancreatic tumor models.^[^ ^33^, ^34^ ^]^ However, side effects due to toxicity were reported in the clinic for other indications demonstrating the need to obtain selective inhibitors.^[^ ^34^ ^]^

BRD family isoform selectivity was achieved with PROTACs, such as MZ1, which degrades BRD4 and is selective over BRD2 and BRD3, two other BET isoforms.^[^ ^35^ ^]^ In addition, a proof of concept study recruiting an E2 enzyme for the development of PROTACs has shown selectivity for BRD4 short isoform (BRD4 S) over BRD4 long isoform (BRD4 L) in breast cancer cells.^[^ ^4^ ^]^ BRD4 isoforms have been reported to have opposing roles in breast cancer development, with the short isoform being oncogenic, whilst the long isoform tumor‐suppressive.^[^ ^36^ ^]^ Whether BRD4 isoforms play the same roles in pancreatic cancer progression remains unknown, and the development and evaluation of BRD4 isoform‐selective PROTACs could be very useful to answer this question.

A study describing Idasanutlin‐based PROTAC A1874 (**Figure** **1B**) identified a synergistic response in HCT116 *via* simultaneous degradation of BRD4 and an increase in p53 and p21 levels, being superior to a VHL‐based counterpart.^[^ ^28^ ^]^ Inspired by this study, the validated well‐characterized VHL‐based PROTAC MZ1, along with its negative control, *cis*MZ1, were used in this work to compare the effect of the E3 ligase in the chosen model.

Nutlin‐3a or (–)‐Nutlin‐3 is the active enantiomer and has a 150‐fold higher binding affinity for MDM2 than the other enantiomer, Nutlin‐3b or (+)‐Nutlin‐3, as determined by surface plasmon resonance (SPR).^[^ ^37^ ^]^ Due to the high cost to isolate the pure enantiomer, the racemic mixture of Nutlin‐3 was used in this study to further validate the applicability of this ligand for PROTAC development (**Figure** **1C**). As indicated by a crystal structure, the imidazoline core replaces the helical backbone of p53 and displaces the Phe19, Leu26, and Trp23 p53 residues with the phenyl rings,^[^ ^37^ ^]^ leaving the piperazinone moiety solvent exposed and suitable for further derivatization and linker attachment.^[^ ^38^ ^]^

**Scheme 1:**
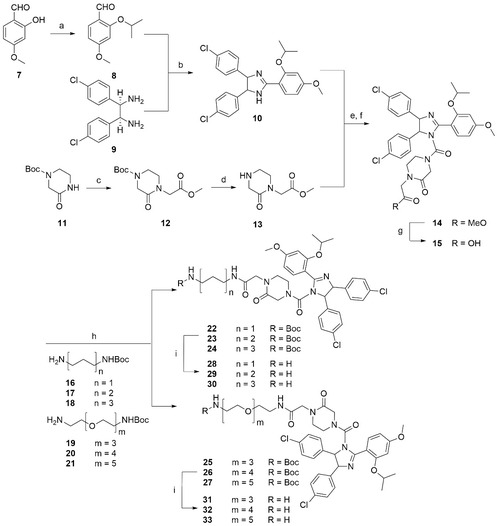
Synthesis of intermediates **28**–**30** and **31**–**33**. Reagents and conditions: a) K_2_CO_3_, ^i^PrBr, Bu_4_NBr, THF, 70°C, 24 h, 69%; b) NBS, DCM, 0°C–rt, 16 h, 44–82%; c) NaH, methyl bromoacetate, THF, 0°C–rt, 18 h, 80–96%; d) 4 M HCl/1,4‐dioxane, rt, 18 h, 65–99%; e) triphosgene, Et_3_N, DCM, 0°C–rt, 18 h; f) **13**, DCM, rt, 2–18 h, 71–88% (over 2 steps); g) LiOH•H_2_O, THF, MeOH, H_2_O, rt, 18 h, 44%–quant.; h) corresponding amine **16**–**21**, HATU, DIPEA, DCM, DMF, rt, 18–60 h, 30–58%; i) 4 M HCl/1,4‐dioxane, 0°C, 1–7 h, 67%–quant.

**Scheme 2:**
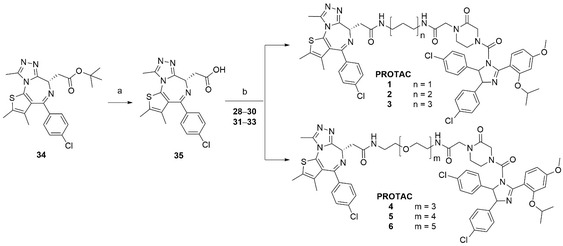
Synthesis of MDM2‐based PROTACs **1**–**6**. Reagents and conditions: a) HCOOH, rt, 48 h, 96%; b) HATU, DIPEA, DMF, rt, 1–16 h, 14–43%.

Several synthetic routes to prepare Nutlin‐3a have been published, particularly for large‐scale synthesis.^[^ ^39^, ^40^ ^]^ Starting from commercially available reagents, the synthesis of the desired six compounds was completed in 10 steps, as described in **Schemes** **1** and **2**.

The *O*‐alkylation of aldehyde **7** was performed in DMF at 40°C in the presence of potassium carbonate, following a literature procedure.^[^ ^41^ ^]^ This route was optimized for scale‐up by replacing DMF with THF and by adding tetrabutylammonium bromide (Bu_4_NBr), which is a phase‐transfer catalyst used to aid solubilization.^[^ ^42^ ^]^ The *cis*‐imidazoline **10** was then synthesized *via* a condensation reaction between aldehyde **8** and the *meso*‐diamine **9**. Then, intermediate **13** was synthesized in 2 steps from the Boc‐protected piperazinone **11**. Next, imidazoline **10** was subjected to a urea formation in the presence of triphosgene, which proceeded by the initial formation of the corresponding carbamoyl chloride *in situ*, followed by the isolation of carbamate **14** upon addition of amine **13**. Following the ester hydrolysis of **14**, carboxylic acid **15** was used to couple the selected linkers (**Scheme** **1**). Then, intermediates **22**–**24** and **25**–**27** were Boc‐deprotected and coupled to the hydrolyzed (+)‐JQ1 reagent **35** to obtain PROTACs **1**–**6** (**Scheme** **2**).

The library of PROTACs was screened at different concentrations after 24‐ and 48‐hour treatment (**Figure S1**, Supporting Information). The PROTACs containing PEG‐based linkers were expected to be more hydrophilic, hence more soluble, than those containing alkyl chains.^[^ ^43^ ^]^ However, both the linker length and the composition influence the PROTAC conformation that allows the ternary complex formation and other cellular degradation kinetics leading to protein degradation.^[^ ^12^, ^44^ ^]^ No trend could be observed after 24‐h treatment with PROTACs **1**–**6** (**Figure S1A**, Supporting Information). After 48‐h treatment, the degradation efficiency increased with the number of methylene units, while all PROTACs incorporating PEG linkers showed a similar pattern (**Figure S1B**, Supporting Information).

**Figure 2:**
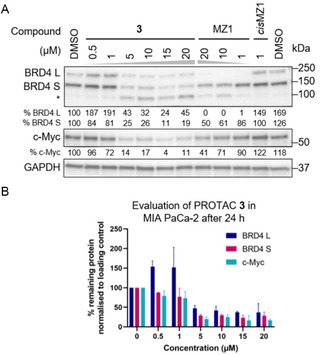
Novel MDM2‐BRD4 PROTACs gave a more pronounced effect on BRD4 S and c‐Myc than MZ1. A) MIA PaCa‐2 cells were treated for 24 h with selected PROTAC **3**, MZ1, and *cis*MZ1 at the indicated concentrations. The increase in BRD4 levels at lower concentrations of PROTAC **3** (0.5–1 μM) could potentially indicate that PROTAC **3** behaves initially as an inhibitor. This feedback is precedented for (+)‐JQ1 and other inhibitors.^[^ ^38^, ^39^, ^40^ ^]^ B) Quantified data are shown as mean ± SD from three independent biological replicates (quantified with ImageJ and analyzed in GraphPad Prism 8.2.0, n = 3, except PROTAC **3** at 0.5 μM, n = 2). The value for each condition was divided by the loading control, then the result was normalized against the left DMSO value to obtain the % remaining of the corresponding protein. *The third band on the BRD4 membrane could indicate proteolytic cleavage of BRD4.

PROTAC **3** emerged as the most potent PROTAC against both BRD4 isoforms (BRD4 L and S) with the most pronounced effect after 48‐hour treatment (**Figure S1**, Supporting Information) and was selected for further investigation (**Figure** **2**). Following a time‐course experiment, the difference in BRD4 S degradation induced by PROTAC **3** and MZ1 became more pronounced after 24‐hour treatment, and this time point was selected for the next experiments (**Figure S2**, Supporting Information).

At concentrations of 5 μM and above of PROTAC **3**, a similar degradation pattern of both BRD4 isoforms could be observed (**Figure** **2A**). PROTAC **3** degraded BRD4 L with a half‐maximal degradation concentration (DC_50_) value of 2.6 μM and BRD4 S with a DC_50_ value of 1.6 μM (**Figure S4**, Supporting Information). The third band appearing at around 100 kDa can be attributed to proteolytic cleavage of BRD4, as it is not present in the DMSO lane or the ones corresponding to the negative controls (**Figure** **2A**).

The levels of c‐Myc, an oncogenic transcription factor whose expression is regulated by BRD4, were also evaluated (**Figure** **2**).^[^ ^45^ ^]^ PROTAC **3** at 15 μM induced ≈77% BRD4 L degradation and ≈83% BRD4 S degradation, leading to ≈86% decrease in c‐Myc levels (**Figure 2B**). While MZ1 appeared to be more effective in degrading BRD4 L compared to PROTAC **3**, it is worth noting that the opposite effect was observed with BRD4 S (**Figure** **2A**). Interestingly, PROTAC **3** was more potent against BRD4 S than MZ1 at the same concentration of 20 μM (**Figure** **2A**). A selectivity screening experiment would determine if the higher decrease in c‐Myc levels observed with PROTAC **3** is due to BRD4 degradation or other off‐target proteins that also regulate c‐Myc transcription.

## 3 Conclusion

To summarize, the design and syntheses of six novel *rac*‐Nutlin‐3‐based PROTACs were reported along with their assessment *in vitro* in a pancreatic cancer model. Selected MDM2‐recruiting PROTAC **3** has shown a higher potency to degrade BRD4 S than MZ1, a validated VHL‐based PROTAC incorporating the same BRD4 ligand, (+)‐JQ1.

To the best of our knowledge, this is the first example of an MDM2‐based PROTAC evaluated in the pancreatic cancer cell line MIA PaCa‐2. The results presented here reinforce the potential of hijacking MDM2 as the E3 ligase for the development of novel PROTACs to degrade key overexpressed proteins in pancreatic cancer.

## Conflict of Interest

The authors declare no conflict of interest.

## Author Contributions

**Mihaela P. Ficu**: Conceptualization (equal); Data curation (lead); Formal analysis (equal); Investigation (lead); Methodology (lead); Validation (lead); Visualization (lead); Writing—original draft (lead); Writing—review & editing (lead). **Dan Niculescu‐Duvaz**: Conceptualization (lead); Methodology (equal); Project administration (supporting); Supervision (equal); Writing—review & editing (lead). **Mohammed Aljarah**: Project administration (supporting); Supervision (supporting); Writing—review & editing (supporting). **Christopher S. Kershaw**: Project administration (supporting); Supervision (supporting); Writing—review & editing (supporting). **Caroline J. Springer**: Conceptualization (supporting); Funding acquisition (lead); Investigation (supporting); Project administration (lead); Resources (lead); Supervision (equal); Writing—review & editing (equal).

## Supporting information

Supplementary Material

## Data Availability

The data that support the findings of this study are available in the supplementary material of this article.
