# Supplementary material for: Hijacking the MDM2 E3 Ligase with Novel BRD4‐Targeting Proteolysis‐Targeting Chimeras in Pancreatic Cancer Cells
Source: Chembiochem. 2025 Jun 23;26(13):e202500133. doi: 10.1002/cbic.202500133 (PMC12247027; doi:10.1002/cbic.202500133)

Hijacking the MDM2 E3 Ligase with novel BRD4-Targeting PROTACs in Pancreatic Cancer Cells

Mihaela P. Ficu,‡ Dan Niculescu-Duvaz,‡,† Mohammed Aljarah,‡,† Christopher S. Kershaw,‡ and Caroline J. Springer*,‡,†

‡Drug Discovery Unit, Cancer Research UK Manchester Institute, University of Manchester, Alderley Park, Macclesfield, SK10 4TG, United Kingdom

†Oncodrug Ltd, Alderley Park, Macclesfield, SK10 4TG, United Kingdom

Contents

1. Additional immunoblotting experiments 2
2. Experimental 4
2.1. Summary of generic conditions 4
2.1.1. Chemicals and solvents 4
2.1.2. Chromatography 4
2.1.3. Analytical techniques 4
2.2. Chemical syntheses 5
3. Biology 18
3.1. Cell line and culture conditions 18
3.2. Immunoblotting 18
4. Characterization data novel compounds 19
5. Full scans of the blots 49

1. Additional immunoblotting experiments

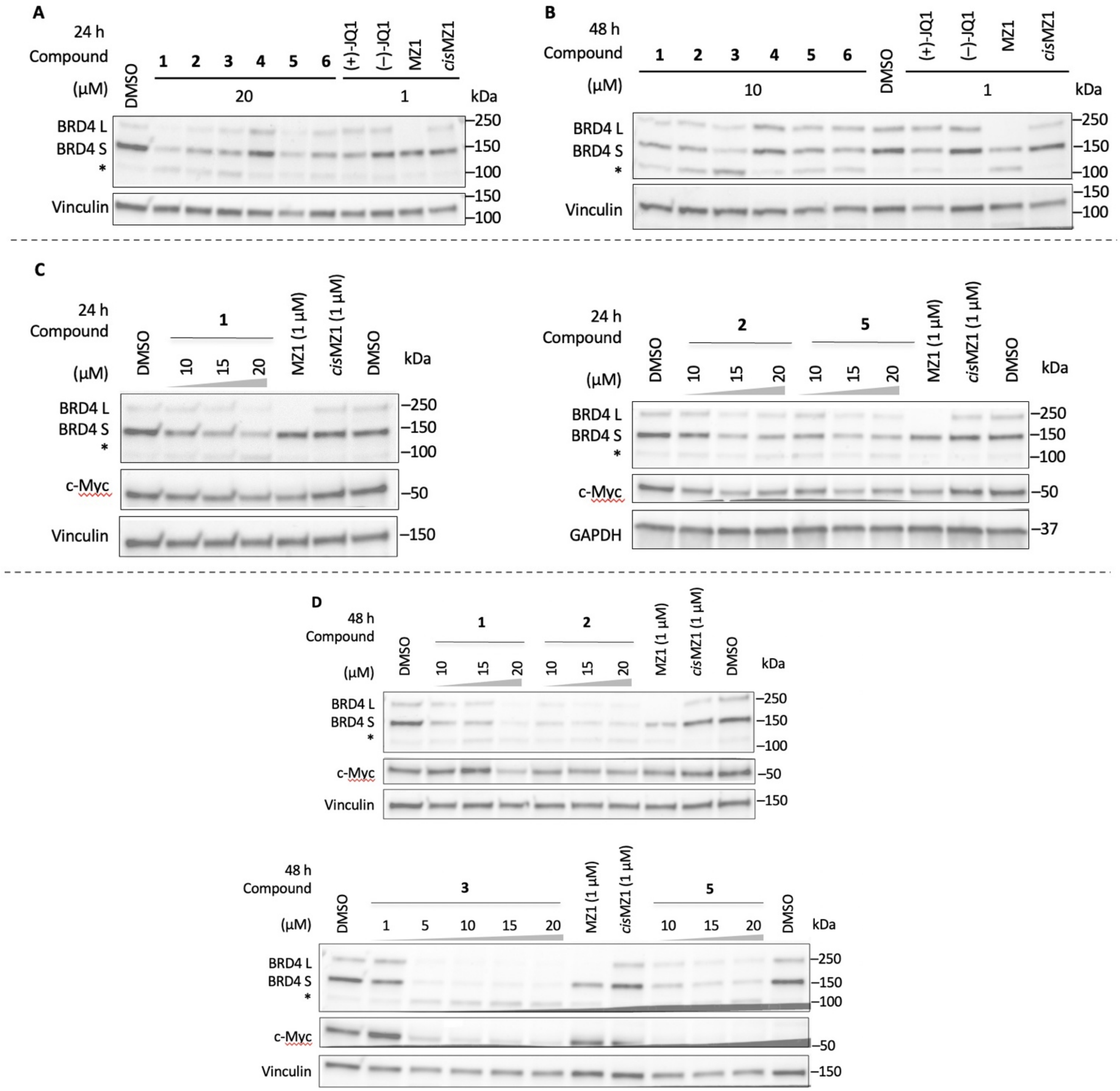

Figure S1. Evaluation of novel MDM2-based PROTACs **1–6** *via* immunoblotting. MIA PaCa-2 cells were treated with the corresponding PROTAC, (+)-JQ1, MZ1 and their negative controls, (−)-JQ1 and *cis*MZ1, at the indicated concentrations for: A) 24 h (n = 2), B) 48 h (n = 2), C) 24 h (n = 1), and D) 48 h (n = 1). *The third band on the BRD4 membrane could indicate proteolytic cleavage of BRD4. The BRD4 membrane was stripped and re-probed with an anti-vinculin antibody.

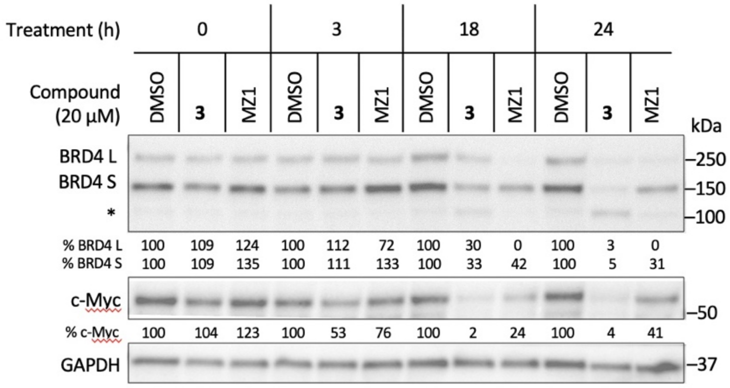

Figure S2. Time course experiment with PROTAC **3**. MIA PaCa-2 cells were treated with PROTAC **3** or MZ1 at 20 µM for the indicated time (n = 1). *The third band on the BRD4 membrane could indicate proteolytic cleavage of BRD4.

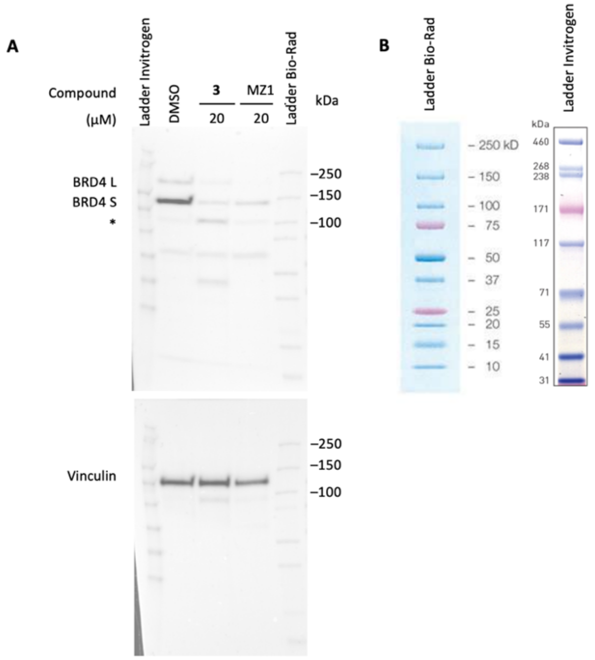

Figure S3. Evaluation of PROTAC **3** and MZ1 *via* immunoblotting. MIA PaCa-2 cells were treated for 24 h with PROTAC **3** and MZ1 at 20 µM (n = 1). A) The entire, uncut membrane was probed with the BRD4 antibody (ab128874), stripped and then re-probed with the Vinculin antibody (V9131). *The third band on the BRD4 membrane could indicate proteolytic cleavage of BRD4. B) Representative images of the molecular weight markers used: Precision Plus Protein™ WesternC™ Blotting Standards (Bio-Rad, 1610376) and HiMark™ Pre-stained Protein Standard (Invitrogen, LC5699).

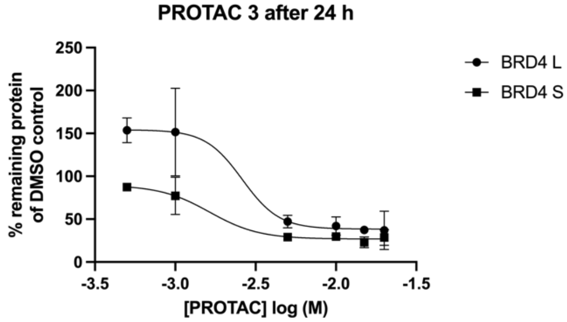

Figure S4. Dose-response curves for the calculation of DC_{50} values for PROTAC **3** evaluated in MIA PACa-2 after 24-h treatment. Quantified data from western blotting experiments are shown as mean ± SD from three independent biological replicates (quantified with ImageJ and analysed in GraphPad Prism 10, n = 3, except PROTAC **3** at 0.5 µM, n = 2). The value for each condition was divided by the loading control, then the result was normalised against the DMSO value to obtain the % remaining of the corresponding protein. The graph was generated using the Dose-response - inhibition function and the Log(inhibitor) vs. response - Variable slope equation in GraphPad Prism 10.

2. Experimental

2.1. Summary of generic conditions

2.1.1. Chemicals and solvents

All commercial reagents were purchased from Advanced ChemBlocks, Fluorochem, Combi-Blocks, Sigma-Aldrich, Alfa Aesar or Apollo Scientific and were used without further purification. (+)-JQ1 was purchased from Advanced ChemBlock for the synthesis of the PROTACs. Anhydrous solvents were obtained from Sigma-Aldrich or Fisher Chemical and were used without further drying. Petroleum ether indicates the fraction with the boiling point 40–60°C. Deuterated solvents were obtained from Sigma-Aldrich, Goss or Fluorochem. Particularly hazardous material: triphosgene (H330: fatal if inhaled)- must be handled in a fume hood.

For biological evaluation: MG132 was purchased from Focus Biomolecules (10-1309, 10-2120). MLN4924 was purchased from Adooq Bioscience (A11260). The (+)-JQ1 and (−)-JQ1 samples used for the western blotting experiments were purchased from Sigma-Aldrich (SML1524, SML1525). MZ1 and *cis*MZ1 were a kind gift Boehringer Ingelheim *via* its open innovation platform opnMe.com.

2.1.2. Chromatography

Flash chromatography was performed using pre-packed silica gel columns (RediSep Rf, Isco) on a Combi*Flash* Rf$^+$ or a Combi*Flash* NextGen. Thin layer chromatography was conducted with 5 × 10 cm plates coated with Merck Type 60 F254 silica gel to a thickness of 0.25 mm.

2.1.3. Analytical techniques

Proton (^{1}H) and carbon (^{13}C) NMR spectra were recorded on a 300 and 75 MHz Bruker spectrometer, respectively. HRMS data were recorded on Agilent LC/MS QTOF 6530 HPLC 1260 Infinity II. Solutions were typically prepared in either deuterated chloroform ($CDCl_3$), deuterated dimethylsulfoxide (DMSO-d_6), deuterated methanol (CD_3OD) or deuterium oxide (D_2O) with chemical shifts referenced to tetramethylsilane (TMS) or deuterated solvent as an internal standard. ^{1}H NMR data were reported indicating the chemical shift (δ) in units of parts per million (ppm), the integration (e.g. 1H), the multiplicity (s, singlet; d, doublet; t, triplet; q, quartet; m, multiplet; br, broad; dd, doublet of doublets etc.) and the coupling constant (*J*) in Hz (app implies apparent coupling on broadened signals). ^{13}C NMR data were reported indicating the chemical shift (δ) in units of parts per million (ppm).

^{1}H and ^{13}C spectra were assigned using 1D and 2D NMR experiments including COSY, HSQC, HMBC and DEPT135.

LC–MS analyses were performed on a Waters Acquity UPLC using BEH C18 1.7 µM columns (2.1 × 50 mm) columns with a diode array detector coupled to a SQD mass spectrometer or a Waters Acquity I-Class UPLC using BEH C18 1.7 µM columns (2.1 × 50 mm) columns with a diode array detector coupled to a QDa mass spectrometer. Analyses were performed with either buffered acidic or basic solvents using gradients as detailed below:

Low pH:

Solvent A – Water + 10 mM ammonium hydrogen carbonate + 0.1% formic acid
Solvent B – MeCN + 5% water + 0.1% formic acid

High pH:

Solvent A – Water + 10 mM ammonium hydrogen carbonate + 0.1% ammonia solution
Solvent B – MeCN + 5% water + 0.1% ammonia solution

Gradient:

Time	Flow rate (mL/min)	% Solvent A	% Solvent B
0	0.6	95	5
1.2	0.6	5	95
1.7	0.6	5	95
1.8	0.6	95	5

All compounds were > 90% purity as determined by examination of both the LC-MS and ^{1}H NMR spectra unless otherwise indicated. Where Cl or Br were present, expected isotopic distribution patterns were observed.

2.2. Chemical syntheses

General methods

Method A: Amide coupling

To a solution of carboxylic acid (1 eq.) in DMF (0.02–0.12 M) were added the amine (1–1.2 eq.), HATU (1.5 eq.) and DIPEA (4 eq.). The mixture was stirred at 0 °C or rt for 1–18 h. Upon completion, the mixture was quenched with H_2O (5 mL) and extracted with Et_2O (6 × 15 mL). The organics were dried over $MgSO_4$ and concentrated *in vacuo*. The crude was purified on a 4.3 g C18 column, eluting with 0–98% MeCN/H_2O. The relevant fractions were concentrated *in vacuo* to afford the title compound.

Method B: Boc deprotection

A solution of Boc-protected amine (1 eq.) in 4 M HCl/1,4-dioxane (0.01–0.1 M) was stirred at rt for 1–3.5 h. Upon completion, the mixture was concentrated *in vacuo* and redissolved in MeOH (5 mL). The solution was loaded on a 5 g SCX HyperSepTM cartridge and eluted with 7 N NH_3/MeOH (2 CV). The resulting material was concentrated *in vacuo* to afford the title compound.

***tert*-butyl 4-(2-methoxy-2-oxoethyl)-3-oxopiperazine-1-carboxylate (12)[1]**

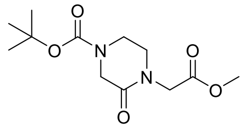

To a solution of *tert*-butyl 3-oxopiperazine-1-carboxylate (2 g, 9.99 mmol, 1 eq.) in THF (40 mL) under an inert atmosphere at 0 °C was added sodium hydride (60% in mineral oil, 459 mg, 11.5 mmol, 1.15 eq.). The reaction mixture was stirred at 0 °C for 1 h, then methyl bromoacetate (1 mL, 11 mmol, 1.1 eq.) was added dropwise *via* syringe. After stirring at 0 °C for 20 min, the reaction mixture was warmed to rt and stirred for 18 h. The mixture was quenched with brine (30 mL) and extracted with EtOAc (3 × 20 mL). The organics were combined, dried over $MgSO_4$ and concentrated *in vacuo*. The crude was washed with petroleum ether and concentrated *in vacuo* to afford the title compound (2.57 g, 9.44 mmol, 94%) as a white solid.

^{1}H NMR (300 MHz, $CDCl_3$)[2] δ 4.17 (s, 2H, NCH_2COO), 4.14 (s, 2H, NCH_2CON), 3.75 (s, 3H, $COOCH_3$), 3.70 (t, *J* = 5.5 Hz, 2H, $CONCH_2$), 3.43 (t, *J* = 5.5 Hz, 2H, $CONCH_2$), 1.47 (s, 9H, 3 × CH_3). LC-MS (high pH) t_R 0.89 min, m/z 273.0 $[M+H]^+$, 96%.

methyl 2-(2-oxopiperazin-1-yl)acetate (13)[3]

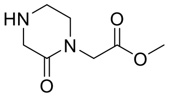

A mixture of **12** (1.42 g, 5.22 mmol, 1 eq.) and 4 M HCl/1,4-dioxane (13 mL, 52.2 mmol, 10 eq.) was stirred at rt for 16 h. The mixture was concentrated *in vacuo*, and the white oil was purified by column chromatography on a 40 g SiO_2 column, eluting with 0-5% [10% NH_4OH/ MeOH]: DCM. The relevant fractions were combined and concentrated *in vacuo* to afford the title compound (796 mg, 4.62 mmol, 89%) as a colourless oil.

^{1}H NMR (300 MHz, $CDCl_3$)[4] δ 4.14 (s, 2H, NCH_2COO), 3.74 (s, 3H, $COOCH_3$), 3.56 (s, 2H, NCH_2CON), 3.40 (t, *J* = 5.5 Hz, 2H, $CONCH_2$), 3.13 (t, *J* = 5.5 Hz, 2H, NCH_2). LC-MS (high pH) t_R 0.44 min, *m/z* 173.1 $[M+H]^+$, 94%.

2-isopropoxy-4-methoxybenzaldehyde (8)[5]

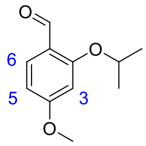

A mixture of 2-hydroxy-4-methoxy-benzaldehyde (120 mg, 0.79 mmol, 1 eq.), tetrabutylammonium bromide (25 mg, 0.08 mmol, 0.1 eq.) and potassium carbonate (166 mg, 1.18 mmol, 1.5 eq.) in THF (5 mL) was stirred at 70 °C for 1.5 h. After cooling to 40 °C, 2-bromopropane (110 µL, 1.18 mmol, 1.5 eq.) was added, and the reaction mixture was stirred at 70 °C for 24 h. The mixture was filtered through a pad of decalite and concentrated *in vacuo*. The mixture was washed with Et_2O and purified by column chromatography on a 12 g SiO_2 column, eluting with 0-30% EtOAc: petroleum ether. The relevant fractions were combined and concentrated *in vacuo* to afford the title compound (106 mg, 0.55 mmol, 69%) as a colourless oil.

^{1}H NMR (300 MHz, $CDCl_3$)[6] δ 10.31 (d, *J* = 0.8 Hz, 1H, CHO), 7.81 (d, *J* = 8.7 Hz, 1H, H-6), 6.52 (ddd, *J* = 8.7, 2.3, 0.8 Hz, 1H, H-5), 6.44 (d, *J* = 2.3 Hz, 1H, H-3), 4.62 (hept, *J* = 6.1 Hz, 1H, C<u>H</u>$(CH_3)_2$), 3.85 (s, 3H, OCH_3), 1.39 (d, *J* = 6.1 Hz, 6H, $CH(\underline{CH_3})_2$). LC-MS (high pH) t_R 1.17 min, *m/z* 195.2 $[M+H]^+$, 97%.

4,5-bis(4-chlorophenyl)-2-(2-isopropoxy-4-methoxyphenyl)-4,5-dihydro-1*H*-imidazole (10)[7]

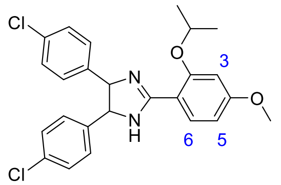

A mixture of **8** (162 mg, 0.83 mmol, 1 eq.) and (1*R*,2*S*)-rel-1,2-bis(4-chlorophenyl)ethane-1,2-diamine (256 mg, 0.91 mmol, 1.09 eq.) in dry DCM (10 mL) was stirred at 0 °C for 30 min under an inert atmosphere. Then, *N*-bromosuccinimide (246 mg, 1.38 mmol, 1.66 eq.) was added, and the resulting mixture was stirred for 16 h at rt under an inert atmosphere. The reaction mixture was quenched with 2 M NaOH (pH 10-11) and extracted with DCM (2 × 10 mL). The organics were combined, washed with brine, passed through a phase separator cartridge, and concentrated *in vacuo*. The crude was purified by column chromatography on a 12 g SiO_2 column, eluting with 0-10% [10% NH_4OH/MeOH]: DCM. The relevant fractions were combined and concentrated *in vacuo* to afford the title compound (254 mg, 0.56 mmol, 67%) as a pale-yellow oil.

^1H NMR (300 MHz, $CDCl_3$) δ 8.53 (d, *J* = 8.8 Hz, 1H, H-6), 7.11 – 6.99 (m, 4H, 4 × H-ArCl), 6.98 – 6.87 (m, 4H, 4 × H-ArCl), 6.68 (dd, *J* = 8.8, 2.3 Hz, 1H, H-5), 6.55 (d, *J* = 2.3 Hz, 1H, H-3), 5.56 (s, 2H, 2 × CHN), 4.74 (hept, *J* = 6.0 Hz, 1H, C<u>H</u>(CH_3)$_2$), 3.89 (s, 3H, OCH_3), 1.39 (d, *J* = 6.0 Hz, 6H, CH(C<u>H_3</u>)$_2$). LC-MS (low pH) t_R 1.20 min, *m/z* 456.9 $[M+H]^+$, 100%.

methyl 2-(4-(4,5-bis(4-chlorophenyl)-2-(2-isopropoxy-4-methoxyphenyl)-4,5-dihydro-1*H*-imidazole-1-carbonyl)-2-oxopiperazin-1-yl)acetate (14)[8]

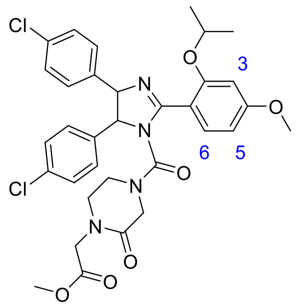

To a stirring mixture of triphosgene (186 mg, 0.62 mmol, 0.8 eq.) in DCM (10 mL) at 0 °C under an inert atmosphere was added dropwise a solution of **10** (356 mg, 0.78 mmol, 1.00 eq.) in anhydrous DCM (10 mL). Then, triethylamine (0.49 mL, 3.52 mmol, 4.5 eq.) was added, and the resulting mixture was stirred for 18 h at rt. A solution of **13** (538 mg, 3.13 mmol, 4 eq.) in anhydrous DCM (5 mL) was added dropwise, and the mixture was stirred under nitrogen for 18 h. The mixture was quenched with MeOH (3 mL), H_2O (10 mL), sat. aq. $NaHCO_3$ (5 mL) (pH 8-9) and extracted with DCM (3 × 20 mL). The organics were combined, washed with brine, dried over $MgSO_4$, filtered, and concentrated *in vacuo.* The crude (860 mg) was purified by column chromatography on a 40 g SiO_2 column, eluting with 0-100% EtOAc:petroleum ether. The relevant fractions were combined and concentrated *in vacuo*, then dissolved in MeOH (2 mL), loaded on a 5 g HyperSep SCX cartridge and eluted with 7 N NH_3/ MeOH to give the title compound (394 mg, 0.6 mmol, 77%) as a white solid.

^1H NMR (300 MHz, CD_3OD) δ 7.60 (d, *J* = 9.0 Hz, 1H, H-6), 7.19 – 7.02 (m, 6H, H-ArCl), 7.00 – 6.92 (m, 2H, H-ArCl), 6.71 (d, *J* = 2.0 Hz, 1H, H-5), 6.69 – 6.66 (m, *J* = 2.0 Hz, 1H, H-3), 5.79 (d, *J* = 10.1 Hz, 1H, CHN), 5.57 (d, *J* = 10.1 Hz, 1H, CHN), 4.81 – 4.68 (m, 1H, C<u>H</u>(CH_3)$_2$), 4.13 (d, *J* = 17.3 Hz, 1H, CH_2), 3.94 – 3.89 (m, 4H, OCH_3, CH_2), 3.88 – 3.81 (m, 2H, CH_2), 3.72 (s, 3H, $COOCH_3$), 3.49 (t, *J* = 5.5 Hz, 2H, CH_2), 3.10 – 3.00 (m, 2H, CH_2), 1.39 (dd, *J* = 9.4, 6.0 Hz, 6H, CH(C<u>H_3</u>)$_2$). LC-MS (high pH) t_R 1.34 min, *m/z* 653.0 $[M+H]^+$, 90%.

2-(4-(4,5-bis(4-chlorophenyl)-2-(2-isopropoxy-4-methoxyphenyl)-4,5-dihydro-1*H*-imidazole-1-carbonyl)-2-oxopiperazin-1-yl)acetic acid (15)[8]

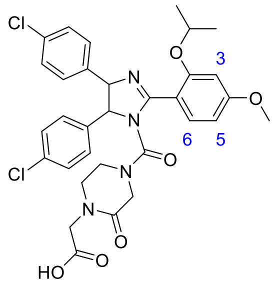

A mixture of **14** (383 mg, 0.58 mmol, 1 eq.) and LiOH•H_2O (39 mg, 0.94 mmol, 1.6 eq.) in THF (12 mL), MeOH (4 mL) and H_2O (4 mL) was stirred at 0 °C for 2 h. The mixture was concentrated *in vacuo* and then partitioned between H_2O (20 mL) and EtOAc

(20 mL). The phases were separated, and the aqueous layer was acidified with 2 M aq. HCl (pH 3–4), then extracted again with EtOAc (6 × 20 mL). The organics were concentrated *in vacuo*, then redissolved in toluene (4 × 10 mL) and concentrated *in vacuo* again to afford the title compound (368 mg, 0.58 mmol, 98%) as a white powder.

^{1}H NMR (300 MHz, CD_3OD) δ 7.66 (d, *J* = 9.3 Hz, 1H, H-6), 7.31 – 7.08 (m, 6H, H-ArCl), 7.00 (d, *J* = 8.4 Hz, 2H, H-ArCl), 6.83 – 6.78 (m, 2H, H-5 and H-3), 6.14 (d, *J* = 10.6 Hz, 1H, CHN), 5.90 (d, *J* = 10.6 Hz, 1H, CHN), 4.95 – 4.89 (m, 1H, C<u>H</u>$(CH_3)_2$), 4.12 (d, *J* = 17.5 Hz, 1H, CH_2), 3.96 (s, 3H, OCH_3), 3.95 – 3.85 (m, 3H, CH_2), 3.58 (t, *J* = 5.5 Hz, 2H, CH_2), 3.13 (t, *J* = 5.5 Hz, 2H, CH_2), 1.44 (d, *J* = 5.6 Hz, 6H, CH(C$\underline{H_3}$)$_2$). LC-MS (high pH) t_R 0.97 min, *m/z* 639.0 $[M+H]^+$, 82%. LC-MS (low pH) t_R 1.13 min, *m/z* 639.0 $[M+H]^+$, 92%.

(*S*)-2-(4-(4-chlorophenyl)-2,3,9-trimethyl-6*H*-thieno[3,2-*f*][1,2,4] triazolo[4,3-*a*][1,4]diazepin-6-yl)acetic acid (35)[9]

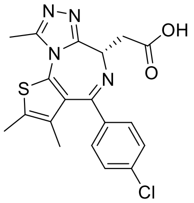

A solution of (*S*)-(+)-*tert*-butyl 2-(4-(4-chlorophenyl)-2,3,9-trimethyl-6*H*-thieno[3,2-*f*][1,2,4]triazolo[4,3-*a*][1,4]diazepin-6-yl)acetate (100 mg, 0.22 mmol, 1 eq.) in formic acid (6 mL) was stirred for 2 days at rt. The mixture was quenched with H_2O (10 mL) and extracted with DCM (5 × 10 mL). The organics were combined, dried over $MgSO_4$, filtered and concentrated *in vacuo* to afford the title compound (85 mg, 0.21 mmol, 96%) as a yellow solid.

^{1}H NMR (300 MHz, $CDCl_3$)[10] δ 7.46 – 7.38 (m, 2H, H-Ar), 7.37 – 7.30 (m, 2H, H-Ar), 4.59 (t, *J* = 6.8 Hz, 1H, NCH), 3.65 (dd, *J* = 13.8, 6.8 Hz, 2H, C$\underline{H_2}$COOH), 2.68 (s, 3H, CH_3), 2.42 (s, 3H, CH_3), 1.70 (s, 3H, CH_3). ^{13}C NMR (75 MHz, $CDCl_3$) δ 172.9 (COOH), 164.5 (q), 155.4 (q), 150.1 (q), 137.2 (q), 136.4 (q), 132.3 (q), 131.3 (q), 131.1 (q), 130.1 (CH-Ar), 129.0 (CH-Ar), 100.1 (q), 53.7 (NCH), 36.8 (<u>C</u>H_2COOH), 14.6 (CH_3), 13.3 (CH_3), 12.0 (CH_3). LC-MS (low pH) t_R 1.10 min, *m/z* 400.9 $[M+H]^+$, 94%.

***tert*-butyl (3-(2-(4-(4,5-bis(4-chlorophenyl)-2-(2-isopropoxy-4-methoxyphenyl)-4,5-dihydro-1*H*-imidazole-1-carbonyl)-2-oxopiperazin-1-yl)acetamido)propyl)carbamate (22)**

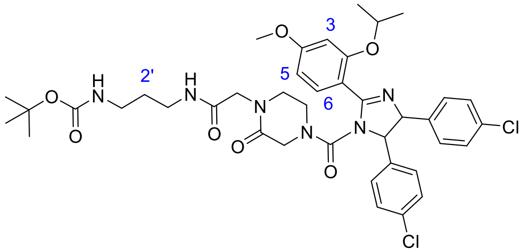

To a solution of **15** (50 mg, 0.08 mmol, 1 eq.) in DCM (3 mL) were added successively *N*,*N*-diisopropylethylamine (30 µL, 0.16 mmol, 2 eq.), 2-(7-aza-1*H*-benzotriazole-1-yl)-1,1,3,3-tetramethyluronium hexafluoro phosphate (HATU) (45 mg, 0.12 mmol, 1.5 eq.) and *tert*-butyl *N*-(3-aminopropyl)carbamate (20 µL, 0.12 mmol, 1.5 eq.). Anhydrous DMF (1 mL) was added, the resulting solution was stirred at rt for 2.5 days, then concentrated *in vacuo*. The residue was dissolved into EtOAc (10 mL) and washed with sat. aq. NH_4Cl (5 mL) and H_2O (10 mL). The aqueous phase was extracted with EtOAc (3 × 10 mL), and the combined organics were dried over $MgSO_4$, filtered and concentrated *in vacuo.* The crude was purified by column chromatography on a 4 g SiO_2 column, eluting with 0-10% MeOH: DCM. The relevant fractions were combined and concentrated *in vacuo*. The mixture was washed with Et_2O, then petroleum ether and the solid filtered to afford the title compound (28 mg, 0.04 mmol, 46%).

^{1}H NMR (300 MHz, CD_3OD) δ 7.61 (d, *J* = 8.3 Hz, 1H, H-6), 7.22 – 7.03 (m, 6H, H-ArCl), 6.97 (d, *J* = 8.5 Hz, 2H, H-ArCl), 6.72 (d, *J* = 2.2 Hz, 1H, H-5), 6.69 (s, 1H, H-3), 5.80 (d, *J* = 10.1 Hz, 1H, CHN), 5.60 (d, *J* = 10.1 Hz, 1H, CHN), 4.77 (app p, *J* = 6.0 Hz, 1H, C<u>H</u>$(CH_3)_2$), 3.97 – 3.75 (m, 7H, CH_2, OCH_3), 3.68 – 3.55 (m, 1H, CH_2), 3.52 – 3.41 (m, 1H, CH_2), 3.21 (t, *J* = 6.6 Hz, 2H, C$\underline{H_2}$NH), 3.17 – 2.98 (m, 4H, CH_2), 1.61 (p, *J* = 6.6 Hz, 2H, H-2'), 1.49 – 1.28 (m, 15H, 3 × CH_3, CH(C$\underline{H_3}$)$_2$). ^{13}C NMR (75 MHz, CD_3OD) δ 169.8 (CO), 167.4 (CO), 165.0 ($COCH_3$), 163.2 (q), 158.7 (CO), 158.5 (<u>C</u>O^{i}Pr), 155.8 (CO), 137.5 (ArCl), 136.4 (ArCl), 134.2 (ArCl), 134.2 (ArCl), 133.0 (C-6), 130.7 (CH-ArCl), 130.0 (CH-ArCl), 129.05 (CH-ArCl), 129.00 (CH-ArCl), 113.7 (q), 106.4 (C-5), 101.2 (C-3), 80.0 (<u>C</u>^{t}Bu), 72.4 (<u>C</u>H$(CH_3)_2$), 72.0 (CHN), 70.0 (CHN), 56.2 (OCH_3), 50.5 (CH_2), 50.5 (CH_2), 48.2 (CH_2), 43.2 (CH_2), 38.3 (CH_2), 37.3 (CH_2), 30.7 (C-2'), 28.8 (^{t}Bu), 22.5 (CH(<u>C</u>H_3)$_2$), 22.4 (CH(<u>C</u>H_3)$_2$). LC-MS (high pH) t_R 1.35 min, *m/z* 794.9 $[M]^+$, 97%.

***tert*-butyl (6-(2-(4-(4,5-bis(4-chlorophenyl)-2-(2-isopropoxy-4-methoxyphenyl)-4,5-dihydro-1*H*-imidazole-1-carbonyl)-2-oxopiperazin-1-yl)acetamido)hexyl)carbamate (23)**

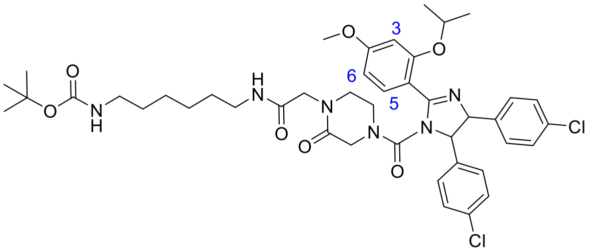

To a solution of **15** (50 mg, 0.08 mmol, 1 eq.) in DCM (3 mL) were added successively *N*,*N*-diisopropylethylamine (30 µL, 0.16 mmol, 2 eq.), HATU (45 mg, 0.12 mmol, 1.5 eq.) and *tert*-butyl *N*-(6-aminohexyl)carbamate (30 µL, 0.12 mmol, 1.5 eq.). Anhydrous DMF (1 mL) was added, the resulting solution was stirred at rt for 2.5 days, then concentrated *in vacuo*. The residue was dissolved into EtOAc (10 mL) and washed with sat. aq. NH_4Cl (5 mL) and H_2O (10 mL). The aqueous phase was extracted with EtOAc (3 × 10 mL), and the combined organics were washed with brine (10 mL), dried over $MgSO_4$, filtered and concentrated *in vacuo*. The crude was purified by column chromatography on a 4 g SiO_2 column, eluting with 0-10% MeOH: DCM. The relevant fractions were combined and concentrated *in vacuo*. The mixture was washed with Et_2O and petroleum ether and purified again by column chromatography on a 4 g SiO_2 column, eluting with 0-10% MeOH: EtOAc. The relevant fractions were combined and concentrated *in vacuo* to afford the title compound (30 mg, 0.03 mmol, 46%) as a white solid.

^{1}H NMR (300 MHz, CD_3OD) δ 7.61 (d, J = 8.8 Hz, 1H, H-6), 7.22 – 7.01 (m, 6H, H-ArCl), 7.01 – 6.91 (m, 2H, H-ArCl), 6.71 (d, J = 2.2 Hz, 1H, H-5), 6.68 (s, 1H, H-3), 5.78 (d, J = 10.0 Hz, 1H, CHN), 5.59 (d, J = 10.0 Hz, 1H, CHN), 4.76 (app p, J = 6.0 Hz, 1H, $C\underline{H}(CH_3)_2$), 3.94 (d, J = 10.9 Hz, 1H, CH_2), 3.90 (s, 3H, OCH_3), 3.88 – 3.76 (m, 3H, CH_2), 3.56 (m, 1H, CH_2), 3.42 (m, 1H, CH_2), 3.17 (t, J = 7.0 Hz, 2H, $C\underline{H_2}NH$), 3.10 – 2.99 (m, 4H, CH_2), 1.55 – 1.43 (m, 13H, 3 × CH_3, CH_2), 1.39 (dd, J = 10.3, 6.0 Hz, 6H, $CH(C\underline{H_3})_2$), 1.35 – 1.29 (m, 4H, CH_2). ^{13}C NMR (75 MHz, CD_3OD) δ 169.6 (CO), 167.3 (CO), 164.8 ($\underline{C}OCH_3$), 163.2 (q), 158.5 ($\underline{C}O^iPr$), 155.8 (CO), 137.6 (ArCl), 136.4 (ArCl), 134.19 (ArCl), 134.18 (ArCl), 133.0 (C-6), 130.7 (CH-ArCl), 129.9 (CH-ArCl), 129.1 (CH-ArCl), 129.0 (CH-ArCl), 113.8 (q), 106.4 (C-5), 101.2 (C-3), 79.8 ($\underline{C}^tBu$), 72.4 ($\underline{C}H(CH_3)_2$), 72.0 (CHN), 70.1 (CHN), 56.2 (OCH_3), 50.5 (CH_2), 50.2 (CH_2), 48.2 (CH_2), 43.2 (CH_2), 41.2 (CH_2), 40.3 (CH_2), 30.8 (CH_2), 30.3 (CH_2), 28.8 (^tBu), 27.5 (CH_2), 27.4 (CH_2), 22.5 ($CH(\underline{C}H_3)_2$), 22.4 ($CH(\underline{C}H_3)_2$). LC-MS (high pH) t_R 1.41 min, *m/z* 837.5 $[M+H]^+$, 94%.

***tert*-butyl (9-amino-9-oxononyl)carbamate (36)**

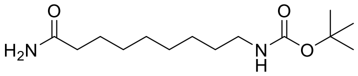

Method adapted from the literature.[11] A solution of 9-((*tert*-butoxycarbonyl)amino)nonanoic acid (334 mg, 1.22 mmol, 1.0 eq.) in anhydrous THF (10 mL) was stirred under nitrogen at −10 °C in a calcium chloride hexahydrate- ice cooling bath (approx. 1:2.5 mass ratio). Triethylamine (0.2 mL, 1.47 mmol, 1.2 eq.) and isobutyl chloroformate (0.21 mL, 1.59 mmol, 1.3 eq.) were added, and the resulting mixture was stirred at −10 °C for 30 min. A solution of aqueous ammonia (35%, 0.7 mL, 6.11 mmol, 5.0 eq.) was added, and the mixture was stirred for 1 h at rt., left to stand for 48 h, then concentrated *in vacuo*. The crude was washed with H_2O (20 mL) and filtered. The white precipitate was redissolved in MeOH (10 mL) upon heating with a heat gun, then concentrated *in vacuo* to give the title compound (264 mg, 0.97 mmol, 79%) as a white powder.

R_f 0.5 (DCM: [2 N NH_3/MeOH] = 9.5: 0.5). ^{1}H NMR (300 MHz, DMSO-d_6) δ 7.20 (s, 1H, 1 × NH_2), 6.74 (t, J = 6.0 Hz, 1H, NH), 6.65 (s, 1H, 1 × NH_2), 2.88 (q, J = 6.8 Hz, 2H, CH_2N), 2.01 (t, J = 7.3 Hz, 2H, CH_2CO), 1.46 (t, J = 7.3 Hz, 2H, $\underline{CH_2}CH_2CO$), 1.37 (s, 10H, CH_2), 1.23 (s, 9H, 3 × CH_3).

***tert*-butyl (9-aminononyl)carbamate (18)**

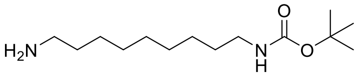

Method adapted from the literature.[12] To a solution of **36** (344 mg, 1.26 mmol, 1.0 eq.) in anhydrous THF (30 mL) at 0 °C under nitrogen, a borane tetrahydrofuran complex solution (1 M in THF, 3.8 mL, 3.79 mmol, 3.0 eq.) was added dropwise. The mixture was heated to 70 °C under nitrogen for 48 h. Additional borane tetrahydrofuran complex solution (1 M/THF, 1.3 mL, 1.26 mmol, 1.0 eq.) was added, and the mixture was stirred at 70 °C under nitrogen for 72 h. The mixture was quenched with MeOH (40 mL), concentrated *in vacuo*, then purified by normal phase chromatography (SiO_2) eluting with 0-20% [10% NH_4OH/MeOH]: DCM. The relevant fractions were combined, concentrated *in vacuo*, dissolved in MeOH (1 mL), loaded on a 5 g HyperSep SCX cartridge, eluted with 7 N NH_3/MeOH and concentrated *in vacuo* to give a colourless oil (139 mg). 20 mg of this material were used in a test reaction. The rest of the material (112 mg) was dissolved in EtOH (15 mL) and heated under reflux to 80 °C for 2 h, then left to stand for 48 h at rt. The mixture was heated again to 80 °C for 18 h, then concentrated *in vacuo*. The mixture was partitioned between H_2O (20 mL)

and DCM (20 mL). The phases were separated and the aqueous phase was extracted with DCM (3 × 20 mL). The organics were combined, washed with brine (10 m), filtered through an Isolute phase separator and concentrated *in vacuo.* The material was purified again by reversed-phase chromatography (C18) eluting with 0-98% $MeCN/H_2O$ to afford the title compound (46 mg, 0.18 mmol, 14%) as a white powder.

^{1}H NMR (300 MHz, $CDCl_3$) δ 4.52 (s, 1H, NH), 3.08 (q, *J* = 6.8 Hz, 2H, C$\underline{H_2}$NH), 2.66 (t, *J* = 6.9 Hz, 2H, C$\underline{H_2}$$NH_2$), 1.58 (app d, *J* = 33.4 Hz, 4H, CH_2), 1.43 (s, 10H, H-3, CH_2), 1.27 (s, 9H, 3 × CH_3). LC-MS (low pH) ELSD t_R 0.95 min, *m/z* 259.06 $[M+H]^+$.

tert-butyl (9-(2-(4-(4,5-bis(4-chlorophenyl)-2-(2-isopropoxy-4-methoxyphenyl)-4,5-dihydro-1*H*-imidazole-1-carbonyl)-2-oxopiperazin-1-yl)acetamido)nonyl)carbamate (24)

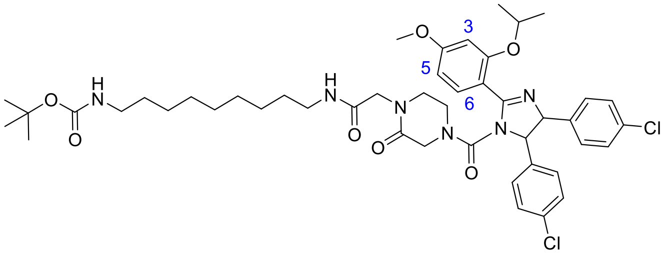

The title compound (40 mg, 0.04 mmol, 58%) was isolated as a white powder by using Method A from **15** (50 mg, 0.08 mmol, 1 eq.), HATU (45 mg, 0.12 mmol, 1.5 eq.), *tert*-butyl (9-aminononyl)carbamate (24 mg, 0.09 mmol, 1.2 eq.) and DIPEA (48 µL, 0.27 mmol, 3.5 eq.) in DMF (5 mL) at rt.

^{1}H NMR (300 MHz, CD_3OD) δ 7.96 (t, *J* = 5.7 Hz, 1H, NH), 7.62 (d, *J* = 9.0 Hz, 1H, H-6), 7.23 – 7.02 (m, 6H, H-Ar), 6.97 (d, *J* = 8.5 Hz, 2H, H-Ar), 6.73 (d, *J* = 2.2 Hz, 1H, H-5), 6.70 (s, 1H, H-3), 5.83 (d, *J* = 10.1 Hz, 1H, NC$\underline{H}$Ph), 5.63 (d, *J* = 10.1 Hz, 1H, NC$\underline{H}$Ph), 4.78 (app p, *J* = 6.0 Hz, 1H, OC$\underline{H}$$(CH_3)_2$), 3.97 (app s, 1H, CH_2), 3.91 (s, 3H, OCH_3), 3.89 – 3.75 (m, 3H, CH_2), 3.64 – 3.50 (m, 1H, CH_2), 3.50 – 3.37 (m, 1H, CH_2), 3.17 (q, *J* = 6.8 Hz, 2H, CH_2), 3.11 – 2.99 (m, 4H, CH_2), 1.45 (s, 9H, 3 × CH_3), 1.39 (dd, *J* = 6.0, 3.9 Hz, 6H, OCH($\underline{CH_3}$)$_2$), 1.33 (s, 14H, CH_2). LC-MS (high pH) t_R 1.52 min, *m/z* 879.3 $[M+H]^+$, 79%.

tert-butyl (1-(4-(4,5-bis(4-chlorophenyl)-2-(2-isopropoxy-4-methoxyphenyl)-4,5-dihydro-1*H*-imidazole-1-carbonyl)-2-oxopiperazin-1-yl)-2-oxo-6,9,12-trioxa-3-azatetradecan-14-yl)carbamate (25)[13]

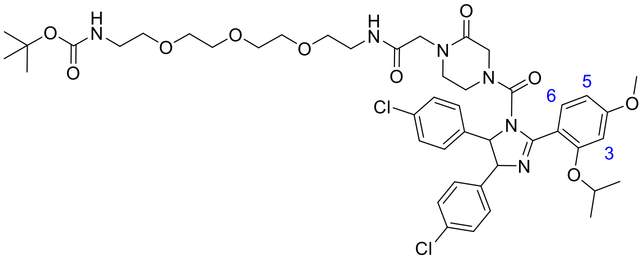

To a solution of **15** (50 mg, 0.08 mmol, 1 eq.) in DCM (3 mL) were added successively *N*,*N*-diisopropylethylamine (30 µL, 0.16 mmol, 2 eq.), HATU (45 mg, 0.12 mmol, 1.5 eq.) and *tert*-butyl *N*-[2-[2-[2-(2-aminoethoxy)ethoxy]ethoxy]ethyl]carbamate (30 µL, 0.12 mmol, 1.5 eq.). Anhydrous DMF (1 mL) was added, the resulting solution was stirred at rt for 2.5 days, then concentrated *in vacuo*. The residue was dissolved into EtOAc (10 mL) and washed with sat. aq. NH_4Cl (5 mL) and H_2O (10 mL). The aqueous phase was extracted with EtOAc (3 × 10 mL), and the combined organics were dried over $MgSO_4$, filtered and concentrated *in vacuo*. The crude was purified by column chromatography on a 4 g SiO_2 column, eluting with 0-10% MeOH: DCM. The relevant fractions were combined and concentrated *in vacuo*. The mixture was washed with Et_2O and petroleum ether and purified again by column chromatography on a 4 g SiO_2 column, eluting with 0-10% MeOH: EtOAc. The relevant fractions were combined and concentrated *in vacuo* to afford the title compound (22 mg, 0.02 mmol, 30%) as a white solid.

^{1}H NMR (300 MHz, CD_3OD) δ 7.61 (d, *J* = 8.8 Hz, 1H, H-6), 7.20 – 7.02 (m, 6H, H-ArCl), 7.00 – 6.94 (m, 2H, H-ArCl), 6.71 (d, *J* = 2.2 Hz, 1H, H-5), 6.68 (s, 1H, H-3), 5.79 (d, *J* = 10.0 Hz, 1H, CHN), 5.59 (d, *J* = 10.0 Hz, 1H, CHN), 4.76 (app p, *J* = 6.0 Hz, 1H, C$\underline{H}$$(CH_3)_2$), 3.98 (d, *J* = 16.3 Hz, 1H, CH_2), 3.91 (s, 3H, OCH_3), 3.88 – 3.78 (m, 3H, CH_2), 3.68 – 3.59 (m, 8H, OCH_2), 3.53 (m, 5H, CH_2), 3.46 (t, *J* = 5.6 Hz, 1H, CH_2), 3.38 (t, *J* = 5.6 Hz, 2H, C$\underline{H_2}$NH), 3.23 (t, *J* = 5.6 Hz, 2H, C$\underline{H_2}$NH), 3.07 (app dd, *J* = 6.3, 5.6 Hz, 2H, CH_2), 1.45 (s, 9H, 3 × CH_3), 1.39 (dd, *J* = 10.3, 6.0 Hz, 6H, CH(C$\underline{H_3}$)$_2$). ^{13}C NMR (75 MHz, CD_3OD) δ 169.8 (CO), 167.3 (CO), 165.0 (CO), 163.2 (q), 158.5 ($\underline{C}$O^{i}Pr), 155.8 (CO), 137.6 (ArCl), 136.4 (ArCl), 134.21 (ArCl), 134.19 (ArCl), 133.1 (C-6), 130.7 (CH-ArCl), 130.0 (CH-ArCl), 129.1 (CH-ArCl), 129.0 (CH-ArCl), 113.8 (q), 106.5 (C-5), 101.4 (CO), 101.2 (C-3), 80.1 ($\underline{C}$^{t}Bu), 72.4 ($\underline{C}$H$(CH_3)_2$), 72.0 (CHN), 71.6 (OCH_2), 71.5 (OCH_2), 71.2 (OCH_2), 71.0 (OCH_2), 70.4 (OCH_2), 70.1 (CHN), 56.2 (OCH_3), 50.5 (CH_2), 50.1 (CH_2), 48.1 (CH_2), 43.2 (CH_2), 41.3 (CH_2NH), 40.4 (CH_2NH), 28.8 (^{t}Bu), 22.5 (CH($\underline{C}H_3$)$_2$), 22.4 (CH($\underline{C}H_3$)$_2$). LC-MS (high pH) t_R 1.34 min, *m/z* 913.5 $[M+H]^+$, 100%.

***tert*-butyl (1-(4-(4,5-bis(4-chlorophenyl)-2-(2-isopropoxy-4-methoxyphenyl)-4,5-dihydro-1*H*-imidazole-1-carbonyl)-2-oxopiperazin-1-yl)-2-oxo-6,9,12,15-tetraoxa-3-azaheptadecan-17-yl)carbamate (26)**

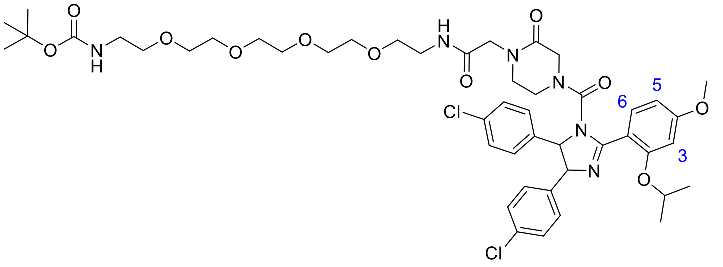

To a solution of **15** (50 mg, 0.08 mmol, 1 eq.) in DCM (3 mL) were added successively *N*,*N*-diisopropylethylamine (30 µL, 0.16 mmol, 2 eq.), HATU (45 mg, 0.12 mmol, 1.5 eq.) and *tert*-butyl *N*-[2-[2-[2-[2-(2-aminoethoxy)ethoxy]ethoxy] ethoxy]ethyl]carbamate (39 mg, 0.12 mmol, 1.5 eq.). Anhydrous DMF (1 mL) was added, the resulting solution was stirred at rt for 2.5 days, then concentrated *in vacuo*. The residue was dissolved into EtOAc (10 mL) and washed with sat. aq. NH_4Cl (5 mL) and H_2O (10 mL). The aqueous phase was extracted with EtOAc (3 × 10 mL), and the combined organics were washed with brine (10 mL), dried over $MgSO_4$, filtered and concentrated *in vacuo*. The crude was purified by column chromatography on a 4 g SiO_2 column, eluting with 0-10% MeOH: DCM. The relevant fractions were combined and concentrated *in vacuo*. The mixture was washed with Et_2O and petroleum ether and purified again by column chromatography on a 4 g SiO_2 column, eluting with 0-10% MeOH: EtOAc. The relevant fractions were combined and concentrated *in vacuo* to afford the title compound (28 mg, 0.03 mmol, 38%) as a white solid.

^{1}H NMR (300 MHz, CD_3OD) δ 7.62 (d, *J* = 8.7 Hz, 1H, H-6), 7.22 – 7.02 (m, 6H, H-ArCl), 7.01 – 6.93 (m, 2H, H-ArCl), 6.72 (d, *J* = 2.2 Hz, 1H, H-5), 6.69 (s, 1H, H-3), 5.79 (d, *J* = 10.0 Hz, 1H, CHN), 5.59 (d, *J* = 10.0 Hz, 1H, CHN), 4.76 (app p, *J* = 6.0 Hz, 1H, C<u>H</u>$(CH_3)_2$), 3.98 (d, *J* = 16.3 Hz, 1H, CH_2), 3.91 (s, 3H, OCH_3), 3.89 – 3.78 (m, 3H, CH_2), 3.68 – 3.61 (m, 12H, OCH_2), 3.59 – 3.49 (m, 5H, CH_2), 3.49 – 3.41 (m, 1H, CH_2), 3.38 (t, *J* = 5.4 Hz, 2H, $C\underline{H_2}NH$), 3.23 (t, *J* = 5.6 Hz, 2H, $C\underline{H_2}NH$), 3.14 – 3.01 (m, 2H, CH_2), 1.46 (s, 9H, 3 × CH_3), 1.40 (dd, *J* = 10.2, 6.0 Hz, 6H, $CH(C\underline{H_3})_2$). ^{13}C NMR (75 MHz, CD_3OD) δ 169.8 (CO), 167.3 (CO), 165.0 (CO), 163.2 (q), 158.5 (CO), 155.8 (CO), 137.6 (ArCl), 136.4 (ArCl), 134.2 (ArCl), 133.0 (C-6), 130.7 (CH-ArCl), 130.0 (CH-ArCl), 129.1 (CH-ArCl), 129.0 (CH-ArCl), 113.8 (q), 106.5 (C-5), 101.2 (C-3), 80.1 ($\underline{C}^t$Bu), 72.4 ($\underline{C}H(CH_3)_2$), 72.0 (CHN), 71.53 (OCH_2), 71.50 (OCH_2), 71.48 (OCH_2), 71.2 (OCH_2), 71.0 (OCH_2), 70.4 (OCH_2), 70.1 (CHN), 56.2 (OCH_3), 50.5 (CH_2), 50.1 (CH_2), 48.1 (CH_2), 43.2 (CH_2), 41.3 (CH_2NH), 40.4 (CH_2NH), 28.8 (^{t}Bu), 22.5 ($CH(\underline{C}H_3)_2$), 22.4 ($CH(\underline{C}H_3)_2$). LC-MS (high pH) t_R 1.34 min, *m/z* 957.5 $[M+H]^+$, 89%.

***tert*-butyl (1-(4-(4,5-bis(4-chlorophenyl)-2-(2-isopropoxy-4-methoxyphenyl)-4,5-dihydro-1*H*-imidazole-1-carbonyl)-2-oxopiperazin-1-yl)-2-oxo-6,9,12,15,18-pentaoxa-3-azaicosan-20-yl)carbamate (27)**

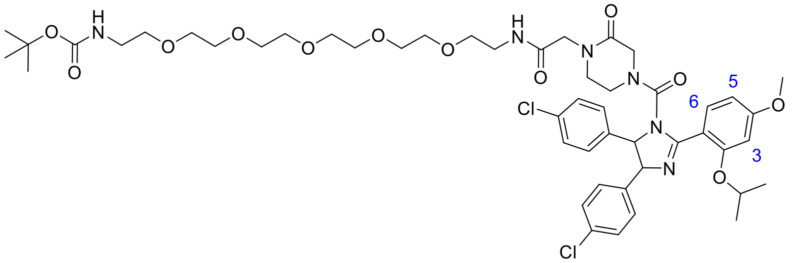

The title compound (42 mg, 0.04 mmol, 53%) was isolated as a white powder by using Method A from **15** (50 mg, 0.08 mmol, 1 eq.) in DMF (5 mL), HATU (45 mg, 0.12 mmol, 1.5 eq.), *tert*-butyl (17-amino-3,6,9,12,15-pentaoxaheptadecyl)carbamate (36 mg, 0.09 mmol, 1.2 eq.) in DCM (1 mL), DIPEA (48 µL, 0.27 mmol, 3.5 eq.) at rt.

^{1}H NMR (300 MHz, CD_3OD) δ 8.03 (t, *J* = 5.5 Hz, 1H, NH), 7.62 (d, *J* = 9.1 Hz, 1H, H-6), 7.22 – 7.03 (m, 6H, H-ArCl), 6.97 (d, *J* = 8.5 Hz, 2H, H-ArCl), 6.74 (d, *J* = 2.2 Hz, 1H, H-5), 6.71 (s, 1H, H-3), 5.90 (d, *J* = 10.3 Hz, 1H, CHN), 5.69 (d, *J* = 10.3 Hz, 1H, CHN), 4.79 (app q, *J* = 6.0 Hz, 1H, $C\underline{H}(CH_3)_2$), 3.98 (d, *J* = 16.3 Hz, 1H, CH_2), 3.91 (s, 3H, OCH_3), 3.89 – 3.79 (m, 3H, CH_2), 3.64 (s, 8H, OCH_2), 3.63 – 3.56 (m, 8H, OCH_2), 3.56 – 3.47 (m, 6H, OCH_2, CH_2), 3.37 (q, *J* = 5.4 Hz, 2H, $C\underline{H_2}NH$), 3.21 (t, *J* = 5.6 Hz, 2H, $C\underline{H_2}NH$), 3.09 (t, *J* = 6.6 Hz, 2H, CH_2), 1.44 (s, 9H, 3 × CH_3), 1.39 (dd, *J* = 6.0, 3.0 Hz, 6H, $CH(C\underline{H_3})_2$). LC–MS (high pH) t_R 1.34 min, *m/z* 1003.2 $[M+H]^+$, 86%. LC-MS (low pH) t_R 1.26 min, *m/z* 1003.3 $[M+H]^+$, 92%.

N-(3-aminopropyl)-2-(4-(4,5-bis(4-chlorophenyl)-2-(2-isopropoxy-4-methoxyphenyl)-4,5-dihydro-1*H*-imidazole-1-carbonyl)-2-oxopiperazin-1-yl)acetamide (28)

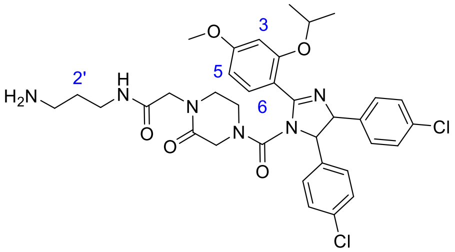

A solution of **22** (28 mg, 0.04 mmol, 1 eq.) in 4 M HCl/1,4-dioxane (0.5 mL, 2 mmol, 56 eq.) was stirred at 0 °C for 5 h. The mixture was concentrated *in vacuo*. Then, the crude was washed with Et_2O, dissolved in MeOH and concentrated *in vacuo*. The mixture was purified by column chromatography on a 4 g SiO_2 column, eluting with 0-20% [10% NH_4OH/ MeOH]: DCM. The relevant fractions were combined and concentrated *in vacuo* to afford the title compound (16 mg, 0.02 mmol, 63%) as a white solid.

^{1}H NMR (300 MHz, CD_3OD) δ 7.61 (d, *J* = 8.7 Hz, 1H, H-6), 7.22 – 7.01 (m, 6H, H-ArCl), 7.07 – 6.91 (m, 2H, H-ArCl), 6.71 (d, *J* = 2.3 Hz, 1H, H-5), 6.68 (s, 1H, H-3), 5.79 (d, *J* = 10.0 Hz, 1H, CHN), 5.59 (d, *J* = 10.0 Hz, 1H, CHN), 4.76 (app p, *J* = 6.0 Hz, 1H, C$\underline{H}$(CH$_3$)$_2$), 4.00 (d, *J* = 17.4 Hz, 1H, CH$_2$), 3.90 (s, 3H, OCH$_3$), 3.89 – 3.76 (m, 3H, CH$_2$), 3.63 – 3.49 (m, 1H, CH$_2$), 3.49 – 3.36 (m, 1H, CH$_2$), 3.26 (t, *J* = 7.0 Hz, 2H, C$\underline{H_2}$NH), 3.18 – 2.98 (m, 2H, CH$_2$), 2.71 (t, *J* = 7.0 Hz, 2H, C$\underline{H_2}$NH$_2$), 1.66 (p, *J* = 7.0 Hz, 2H, H-2'), 1.39 (dd, *J* = 10.5, 6.0 Hz, 6H, CH(C$\underline{H_3}$)$_2$). ^{13}C NMR (75 MHz, CD_3OD) δ 170.1 (CO), 167.4 (CO), 165.0 (CO), 163.2 (q), 158.5 (CO), 155.8 (CO), 137.6 (ArCl), 136.4 (ArCl), 134.2 (ArCl), 133.1 (C-6), 130.7 (CH-ArCl), 130.0 (CH-ArCl), 129.1 (CH-ArCl), 129.0 (CH-ArCl), 113.8 (q), 106.4 (C-5), 101.3 (C-3), 72.5 ($\underline{C}$H(CH$_3$)$_2$), 72.0 (CHN), 70.0 (CHN), 56.2 (OCH$_3$), 50.5 (CH$_2$), 50.4 (CH$_2$), 48.3 (CH$_2$), 43.2 (CH$_2$), 39.3 (CH$_2$), 37.6 (CH$_2$), 32.2 (CH$_2$, C-2'), 22.5 (CH($\underline{C}$H$_3$)$_2$), 22.4 (CH($\underline{C}$H$_3$)$_2$). LC-MS (high pH) t_R 1.14 min, *m/z* 695.3 [M+H]$^+$, 100%.

N-(6-aminohexyl)-2-(4-(4,5-bis(4-chlorophenyl)-2-(2-isopropoxy-4-methoxyphenyl)-4,5-dihydro-1*H*-imidazole-1-carbonyl)-2-oxopiperazin-1-yl)acetamide (29)

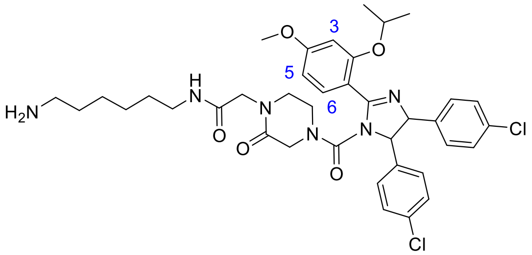

A solution of **23** (30 mg, 0.04 mmol, 1 eq.) in 4 M HCl/1,4-dioxane (0.5 mL, 2 mmol, 55.9 eq.) was stirred at 0 °C for 4 h. The mixture was concentrated *in vacuo* to afford the title compound (31 mg, 0.04 mmol, quant.) as a white solid.

^{1}H NMR (300 MHz, CD_3OD) δ 7.73 (d, *J* = 8.9 Hz, 1H, H-6), 7.31 – 7.12 (m, 6H, H-ArCl), 7.06 (d, *J* = 8.1 Hz, 2H, H-ArCl), 6.90 – 6.77 (m, 2H, H-5, H-3), 6.32 (d, *J* = 10.0 Hz, 1H, CHN), 6.12 (d, *J* = 10.0 Hz, 1H, CHN), 5.03 – 4.92 (m, 1H, C$\underline{H}$(CH$_3$)$_2$), 4.08 – 4.00 (m, 1H, CH$_2$), 3.97 (s, 3H, OCH$_3$), 3.96 – 3.81 (m, 3H, CH$_2$), 3.80 – 3.68 (m, 1H, CH$_2$), 3.64 – 3.53 (m, 1H, CH$_2$), 3.26 – 3.10 (m, 4H, CH$_2$), 2.93 (t, *J* = 7.6 Hz, 2H, CH$_2$), 1.66 (p, *J* = 7.2 Hz, 2H, CH$_2$), 1.54 (p, *J* = 6.7 Hz, 2H, CH$_2$), 1.49 – 1.34 (m, 10H, CH$_2$, CH(C$\underline{H_3}$)$_2$). ^{13}C NMR (75 MHz, CD_3OD) δ 169.6 (CO), 168.6 (CO), 167.0 (CO), 166.4 (q), 160.0 (CO), 151.3 (CO), 136.0 (ArCl), 134.0 (C-6), 132.9 (ArCl), 132.4 (ArCl), 130.6 (CH-ArCl), 130.4 (CH-ArCl), 129.8 (CH-ArCl), 129.6 (CH-ArCl), 108.2 (C-5), 105.0 (q), 101.6 (C-3), 73.9 ($\underline{C}$H(CH$_3$)$_2$), 70.9 (CHN), 64.9 (CHN), 56.8 (OCH$_3$), 50.4 (CH$_2$), 48.0 (CH$_2$), 43.8 (CH$_2$), 40.6 (CH$_2$), 40.2 (CH$_2$,), 30.0 (CH$_2$), 28.4 (CH$_2$), 27.3 (CH$_2$), 27.0 (CH$_2$), 22.31 (CH($\underline{C}$H$_3$)$_2$), 22.26 (CH($\underline{C}$H$_3$)$_2$). LC-MS (high pH) t_R 1.15 min, *m/z* 737.4 [M+H]$^+$, 100%.

N-(9-aminononyl)-2-(4-(4,5-bis(4-chlorophenyl)-2-(2-isopropoxy-4-methoxyphenyl)-4,5-dihydro-1*H*-imidazole-1-carbonyl)-2-oxopiperazin-1-yl)acetamide (30)

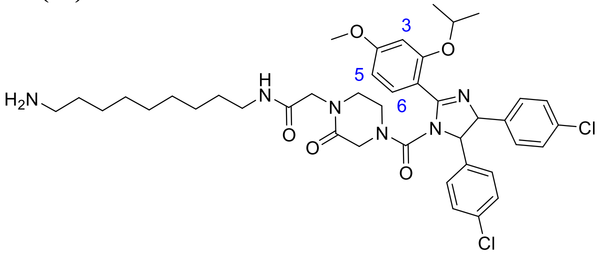

The title compound (30 mg, 0.04 mmol, 87%) was isolated as a white powder by using Method B from **24** (39 mg, 0.04 mmol, 1 eq.) and 4 M HCl/1,4-dioxane (4 mL, 16 mmol, 363 eq.).

^{1}H NMR (300 MHz, CD_3OD) δ δ 7.59 (d, *J* = 8.7 Hz, 1H, H-6), 7.17 – 7.01 (m, 6H, H-Ar), 6.95 (d, *J* = 8.5 Hz, 2H, H-Ar), 6.69 (d, *J* = 2.3 Hz, 1H, H-5), 6.66 (s, 1H, H-3), 5.77 (d, *J* = 10.0 Hz, 1H, $NC\underline{H}Ph$), 5.57 (d, *J* = 10.0 Hz, 1H, $NC\underline{H}Ph$), 4.74 (app p, *J* = 6.0 Hz, 1H, $OC\underline{H}(CH_3)_2$), 3.88 (app s, 3H, OCH_3), 3.96 – 3.75 (m, 4H, CH_2), 3.57 – 3.48 (m, 1H, CH_2), 3.47 – 3.37 (m, 1H, CH_2), 3.15 (t, *J* = 7.1 Hz, 2H, CH_2), 3.09 – 2.98 (m, 2H, CH_2), 2.72 (t, *J* = 7.4 Hz, 2H, CH_2), 1.59 – 1.44 (m, 4H, CH_2), 1.37 (dd, *J* = 10.6, 6.0 Hz, 6H, $OCH(\underline{CH_3})_2$), 1.32 (s, 10H, CH_2). ^{13}C NMR (75 MHz, CD_3OD) δ 169.6, 167.3, 165.0, 163.2, 158.5, 155.8, 137.6, 136.4, 134.2, 133.1, 130.7, 129.9, 129.1, 129.0, 113.8, 106.5, 101.2, 72.4, 72.1, 70.1, 56.2, 50.5, 50.2, 43.2, 41.8, 40.4, 31.5, 30.5, 30.4, 30.3, 27.9, 27.7, 22.5, 22.4. LC-MS (high pH) t_R 1.08 min, *m/z* 781.1 $[M+H]^+$, 83%. LC-MS (low pH) t_R 1.28 min, *m/z* 781.1 $[M+H]^+$, 77%.

***N*-(2-(2-(2-(2-aminoethoxy)ethoxy)ethoxy)ethyl)-2-(4-(4,5-bis(4-chlorophenyl)-2-(2-isopropoxy-4-methoxyphenyl)-4,5-dihydro-1*H*-imidazole-1-carbonyl)-2-oxopiperazin-1-yl)acetamide (31)[13]**

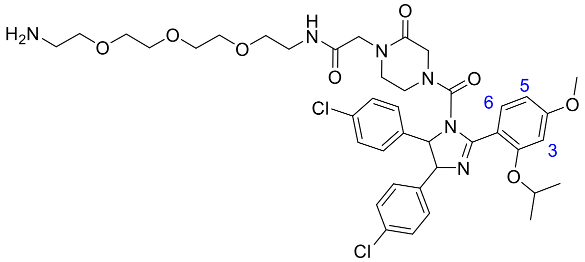

A solution of **25** (22 mg, 0.02 mmol, 1 eq.) in 4 M HCl/1,4-dioxane (1 mL, 4 mmol, 169 eq.) was stirred at 0 °C for 7 h. The mixture was concentrated *in vacuo* and purified by column chromatography on a 4 g SiO_2 column, eluting with 0–20% [10% NH_4OH/MeOH]/DCM. The relevant fractions were concentrated *in vacuo* to afford the title compound (13 mg, 0.02 mmol, 67%) as a white solid.

^{1}H NMR (300 MHz, CD_3OD) δ 7.61 (d, *J* = 8.2 Hz, 1H, H-6), 7.21 – 7.03 (m, 6H, H-ArCl), 6.97 (d, *J* = 8.3 Hz, 2H, H-ArCl), 6.72 (d, *J* = 2.2 Hz, 1H, H-5), 6.69 (s, 1H, H-3), 5.79 (d, *J* = 10.0 Hz, 1H, CHN), 5.59 (d, *J* = 10.0 Hz, 1H, CHN), 4.76 (app p, *J* = 6.0 Hz, 1H, $C\underline{H}(CH_3)_2$), 3.97 (d, *J* = 16.3 Hz, 1H, CH_2), 3.91 (s, 3H, OCH_3), 3.88 – 3.80 (m, 3H, CH_2), 3.69 – 3.61 (m, 8H, OCH_3), 3.55 (app q, *J* = 5.0 Hz, 5H, CH_2), 3.50 – 3.42 (m, 1H, CH_2), 3.38 (t, *J* = 5.4 Hz, 2H, $C\underline{H_2}NH$), 3.07 (app dd, *J* = 5.6, 4.9 Hz, 2H, CH_2), 2.85 (t, *J* = 5.0 Hz, 2H, $C\underline{H_2}NH_2$), 1.39 (dd, *J* = 10.5, 6.0 Hz, 6H, $CH(C\underline{H_3})_2$). ^{13}C NMR (75 MHz, CD_3OD) δ 169.8 (CO), 167.3 (CO), 165.0 (CO), 163.2 (q), 158.5 (CO), 155.8 (CO), 137.6 (ArCl), 136.4 (ArCl), 134.2 (ArCl), 133.0 (C-6), 130.7 (CH-ArCl), 130.0 (CH-ArCl), 129.1 (CH-ArCl), 129.0 (CH-ArCl), 113.8 (q), 106.5 (C-5), 101.3 (C-3), 72.6 (CH_2), 72.4 ($\underline{C}H(CH_3)_2$), 72.0 (CHN), 71.53 (OCH_2) , 71.51 (OCH_2), 71.22 (OCH_2), 71.19 (OCH_2), 70.4 (OCH_2), 70.1 (CHN), 56.2 (OCH_3), 50.5 (CH_2), 50.2 (CH_2), 48.2 (CH_2), 43.2 (CH_2), 41.9 (CH_2), 40.4 (CH_2), 22.5 ($CH(\underline{C}H_3)_2$), 22.4 ($CH(\underline{C}H_3)_2$). LC-MS (high pH) t_R 1.14 min, *m/z* 813.4 $[M+H]^+$, 100%.

***N*-(14-amino-3,6,9,12-tetraoxatetradecyl)-2-(4-(4,5-bis(4-chlorophenyl)-2-(2-isopropoxy-4-methoxyphenyl)-4,5-dihydro-1*H*-imidazole-1-carbonyl)-2-oxopiperazin-1-yl)acetamide (32)**

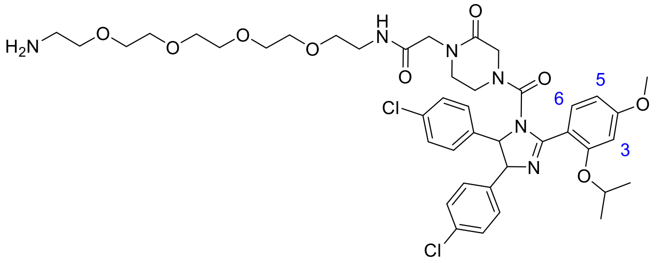

A solution of **26** (28 mg, 0.03 mmol, 1 eq.) in 4 M HCl/1,4-dioxane (0.5 mL, 2 mmol, 67.9 eq.) was stirred at 0 °C for 5 h. The mixture was concentrated *in vacuo*, washed with Et_2O, then concentrated *in vacuo* again to afford the title compound (28 mg, 0.03 mmol, quant.) as a white solid.

^{1}H NMR (300 MHz, CD_3OD) δ (300 MHz, CD_3OD) δ 7.72 (d, *J* = 9.3 Hz, 1H, H-6), 7.34 – 7.11 (m, 6H, H-ArCl), 7.05 (d, *J* = 8.3 Hz, 2H, H-ArCl), 6.88 – 6.85 (m, 1H, H-5), 6.84 (d, *J* = 2.5 Hz, 1H, H-3), 6.27 (d, *J* = 10.6 Hz, 1H, CHN), 6.05 (d, *J* = 10.6 Hz, 1H, CHN), 5.04 – 4.92 (m, 1H, $C\underline{H}(CH_3)_2$), 4.11 – 3.84 (m, 7H, CH_2, OCH_3), 3.76 – 3.64 (m, 16H, CH_2), 3.57 (t, *J* = 5.6 Hz, 2H, OCH_2), 3.44 – 3.36 (m, 2H, $C\underline{H_2}NH$), 3.22 – 3.10 (m, 4H, $C\underline{H_2}NH_2$), 1.48 (app t, *J* = 6.4 Hz, 6H, $CH(C\underline{H_3})_2$). ^{13}C NMR (75 MHz, CD_3OD) δ 169.8 (CO), 168.6 (CO), 167.0 (CO), 166.4 (q), 160.0 (CO), 151.3 (CO), 136.0 (ArCl), 134.0 (C-6), 133.0 (ArCl), 132.4 (ArCl), 130.7 (CH-ArCl), 130.4 (CH-ArCl), 129.8 (CH-ArCl), 129.6 (CH-ArCl), 108.3 (C-5b), 105.0 (q), 101.6 (C-3), 73.9 ($\underline{C}H(CH_3)_2$), 71.42 (OCH_2), 71.38 (OCH_2), 71.3 (OCH_2), 71.2 (OCH_2), 71.0 (OCH_2), 70.9 (CHN), 70.5 (OCH_2), 68.1 (OCH_2), 67.8 (OCH_2), 64.9

(CHN), 56.8 (OCH_3), 50.4 (CH_2), 48.0 (CH_2), 43.8 (CH_2), 40.6 (CH_2), 40.3 (CH_2), 22.33 ($CH(\underline{C}H_3)_2$), 22.27 ($CH(\underline{C}H_3)_2$). LC-MS (high pH) t_R 1.14 min, *m/z* 857.5 $[M+H]^+$, 100%.

_N_-(17-amino-3,6,9,12,15-pentaoxaheptadecyl)-2-(4-(4,5-bis(4-chlorophenyl)-2-(2-isopropoxy-4-methoxyphenyl)-4,5-dihydro-1_H_-imidazole-1-carbonyl)-2-oxopiperazin-1-yl)acetamide (33)

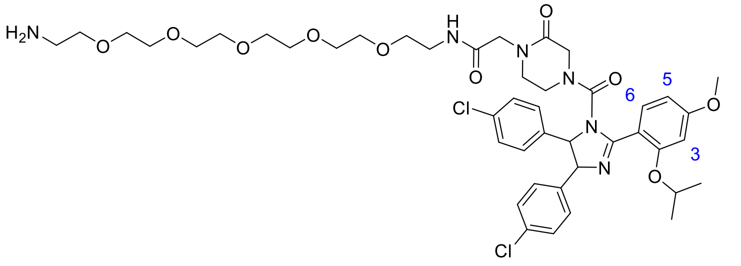

The title compound (34 mg, 0.04 mmol, 99%) was isolated as a white powder by using Method B from **27** (39 mg, 0.04 mmol, 1 eq.) and 4 M HCl/1,4-dioxane (4 mL, 16 mmol, 416 eq.).

^{1}H NMR (300 MHz, CD_3OD) δ 7.59 (d, *J* = 8.8 Hz, 1H, H-6), 7.17 – 7.01 (m, 6H, H-Ar), 6.98 – 6.92 (m, 2H, H-Ar), 6.69 (d, *J* = 2.2 Hz, 1H, H-5), 6.67 (d, *J* = 1.2 Hz, 1H, H-3), 5.77 (d, *J* = 10.0 Hz, 1H, NC$\underline{H}$Ph), 5.57 (d, *J* = 10.0 Hz, 1H, NC$\underline{H}$Ph), 4.74 (app p, *J* = 6.0 Hz, 1H, $OC\underline{H}(CH_3)_2$), 3.95 (d, *J* = 16.3 Hz, 1H, CH_2), 3.88 (s, 3H, OCH_3), 3.87 – 3.79 (m, 3H, CH_2), 3.68 – 3.59 (m, 16H, 8 × OCH_2), 3.59 – 3.49 (m, 5H, CH_2), 3.48 – 3.39 (m, 1H, CH_2), 3.38 – 3.35 (m, 2H, CH_2), 3.11 – 3.00 (m, 2H, CH_2), 2.86 (t, *J* = 5.3 Hz, 2H, CH_2), 1.37 (dd, *J* = 10.4, 6.0 Hz, 6H, $OCH(\underline{CH_3})_2$). ^{13}C NMR (75 MHz, CD_3OD) δ 169.9, 167.3, 165.0, 163.2, 158.5, 155.8, 137.6, 136.5, 134.2, 133.0, 130.7, 130.0, 129.1, 129.0, 113.8, 106.5, 101.2, 72.4, 72.1, 71.9, 71.5, 71.4, 71.19, 71.16, 70.4, 70.1, 56.2, 50.5, 50.2, 43.2, 41.7, 40.4, 22.5, 22.4. LC-MS (high pH) t_R 1.21 min, *m/z* 903.4 $[M+H]^+$, 93%. LC-MS (low pH) t_R 1.00 min, *m/z* 903.4 $[M+H]^+$, 92%.

2-(4-((4_S_,5_R_)-4,5-bis(4-chlorophenyl)-2-(2-isopropoxy-4-methoxyphenyl)-4,5-dihydro-1_H_-imidazole-1-carbonyl)-2-oxopiperazin-1-yl)-_N_-(3-(2-((_S_)-4-(4-chlorophenyl)-2,3,9-trimethyl-6_H_-thieno[3,2-f][1,2,4]triazolo[4,3-a][1,4]diazepin-6-yl)acetamido)propyl) acetamide (1)

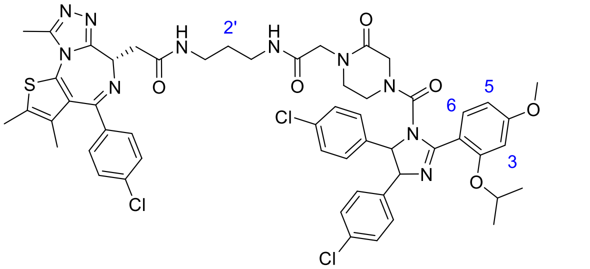

To a solution of **35** (9 mg, 0.02 mmol, 1 eq.) in DMF (0.5 mL) was added HATU (10 mg, 0.03 mmol, 1.2 eq.) and *N*,*N*-diisopropylethylamine (14 µL, 0.08 mmol, 3.5 eq.). After stirring for 10 min at rt, **28** (16 mg, 0.02 mmol, 1 eq.) was added, and the reaction mixture was stirred for 16 h. The mixture was cooled in an ice bath, quenched with H_2O (5 mL) and extracted with a solution of 10% MeOH in DCM (3 × 10 mL). The combined organics were washed with brine (10 mL), dried over $MgSO_4$, filtered and concentrated *in vacuo*. The crude was purified by column chromatography on a 4 g SiO_2 column, eluting with 0-10% [MeOH + 10% 2 M NH_3/MeOH]: DCM. The relevant fractions were combined and concentrated *in vacuo*. The oil was further purified by reverse phase chromatography (4 g C18 column), eluting with 5-95% MeCN: H_2O. The appropriate fractions were combined and concentrated *in vacuo* to afford the title compound (10 mg, 0.01 mmol, 42%) as a white solid (mixture of diastereoisomers).

^{1}H NMR (300 MHz, CD_3OD) δ 7.56 (dd, *J* = 9.1, 1.4 Hz, 1H, H-6), 7.50 – 7.37 (m, 4H, H-Ar), 7.19 – 7.12 (m, 2H, H-Ar), 7.12 – 6.99 (m, 4H, H-Ar), 6.99 – 6.89 (m, 2H, H-Ar), 6.70 – 6.60 (m, 2H, H-5, H-3), 5.75 (dd, *J* = 10.0, 4.0 Hz, 1H, CHN), 5.57 (dd, *J* = 10.0, 1.3 Hz, 1H, CHN), 4.73 (app p, *J* = 6.0 Hz, 1H, $C\underline{H}(CH_3)_2$), 4.67 – 4.58 (m, 1H, $NC\underline{H}CH_2$), 3.96 – 3.71 (m, 7H, CH_2, OCH_3), 3.61 – 3.50 (m, 1H, CH_2), 3.46 – 3.35 (m, 3H, CH_2), 3.31 – 3.18 (m, 4H, $C\underline{H_2}NH$), 3.10 – 3.01 (m, 2H, CH_2), 2.70 (d, *J* = 2.4 Hz, 3H, CH_3), 2.45 (s, 3H, CH_3), 1.79 – 1.65 (m, 5H, CH_3, H-2'), 1.37 (dd, *J* = 9.1, 6.0 Hz, 6H, $CH(C\underline{H_3})_2$). ^{13}C NMR (75 MHz, CD_3OD) δ 172.9 (CO), 169.8 (CO), 167.4 (CO), 166.3 (q), 164.9 (CO), 163.2 (q), 158.5 (CO), 157.0 (CO), 155.7 (q), 152.2 (q), 138.1 (ArCl), 138.0 (ArCl), 137.6 (ArCl), 136.4 (ArCl), 134.2 (ArCl), 133.0 (C-6), 132.0 (q), 131.9 (q), 131.3 (q), 130.7 (CH-ArCl), 130.1 (CH-ArCl), 129.8 (CH-ArCl), 129.05 (CH-ArCl), 129.01 (CH-ArCl), 128.99 (CH-ArCl), 113.8 (C), 106.4 (C-5), 101.4 (q), 101.2 (C-3), 72.4 ($\underline{C}H(CH_3)_2$), 72.1 (CHN), 70.0 (CHN), 56.2 (OCH_3), 55.2 ($N\underline{C}HCH_2$), 50.7 (CH_2), 50.5 (CH_2), 48.3 (CH_2), 42.9 (CH_2), 38.8 (CH_2), 37.6 (CH_2), 37.4 (CH_2), 30.1 (C-2'), 22.5 ($CH(\underline{C}H_3)_2$), 22.4 ($CH(\underline{C}H_3)_2$), 14.5 (CH_3), 13.0 (CH_3), 11.6 (CH_3). LC-MS (high pH) t_R 1.41 min, *m/z* 1078.8 $[M+H]^+$, 91%. LC-MS (low pH) t_R 1.33 min, *m/z* 1078.7 $[M+H]^+$, 95%. HRMS (ESI^+) calculated for $C_{54}H_{55}Cl_3N_{10}O_6S$ 1076.3092, found 1076.3084.

2-(4-((4*R*,5*S*)-4,5-bis(4-chlorophenyl)-2-(2-isopropoxy-4-methoxyphenyl)-4,5-dihydro-1*H*-imidazole-1-carbonyl)-2-oxopiperazin-1-yl)-*N*-(6-(2-((*S*)-4-(4-chlorophenyl)-3,9-dimethyl-6*H*-thieno[3,2-f][1,2,4]triazolo[4,3-a][1,4]diazepin-6-yl)acetamido)hexyl) acetamide (2)

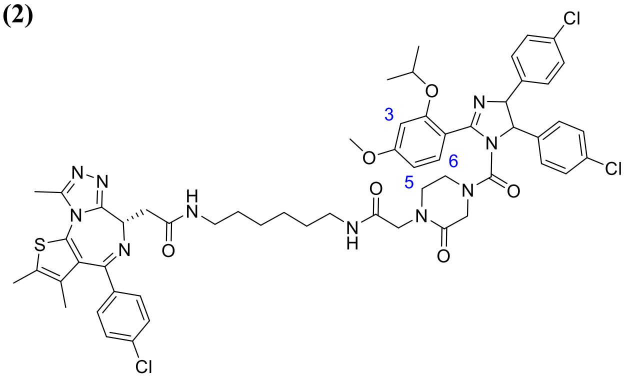

To a solution of **35** (16 mg, 0.04 mmol, 1 eq.) in DMF (0.5 mL) was added HATU (18 mg, 0.05 mmol, 1.2 eq.) and *N*,*N*-diisopropylethylamine (24 µL, 0.14 mmol, 3.5 eq.). After stirring for 1 h at rt, **29** (31 mg, 0.04 mmol, 1 eq.) in DMF (0.5 mL) was added, and the reaction mixture was stirred for 16 h. The mixture was cooled in an ice bath, quenched with H_2O (5 mL) and extracted with EtOAc (2 × 10 mL), then with a solution of 10% MeOH in DCM (2 × 10 mL). The combined organics were washed with brine (10 mL), dried over $MgSO_4$, filtered and concentrated *in vacuo*. The crude was purified by column chromatography on a 4 g SiO_2 column, eluting with 0-20% [10% NH_4OH/ MeOH]: DCM. The relevant fractions were combined, concentrated *in vacuo*, washed with Et_2O and petroleum ether and concentrated *in vacuo* again. The residue was further purified by column chromatography on a 4 g SiO_2 column, eluting with 0-10% [MeOH + 10% 2 M NH_3/ MeOH]: DCM. The relevant fractions were combined and concentrated *in vacuo*. The mixture was further purified by reverse phase chromatography (4 g C18 column), eluting with 5-95% MeCN: H_2O. The appropriate fractions were combined and concentrated *in vacuo* to afford the title compound (7.8 mg, 0.01 mmol, 17%) as a white solid (mixture of diastereoisomers).

^{1}H NMR (300 MHz, CD_3OD) δ 7.58 (d, *J* = 8.3 Hz, 1H, H-6), 7.48 – 7.37 (m, 4H, H-Ar), 7.18 – 6.99 (m, 6H, H-Ar), 6.94 (d, *J* = 8.3 Hz, 2H, H-Ar), 6.68 (d, *J* = 2.2 Hz, 1H, H-5), 6.65 (s, 1H, H-3), 5.76 (d, *J* = 10.0 Hz, 1H, CHN), 5.56 (d, *J* = 10.0 Hz, 1H, CHN), 4.76 – 4.68 (m, 1H, $C\underline{H}(CH_3)_2$), 4.63 (dd, *J* = 8.6, 5.7 Hz, 1H, $NC\underline{H}CH_2$), 3.93 (s, 1H, CH_2), 3.88 (s, 3H, OCH_3), 3.85 – 3.74 (m, 3H, OCH_3), 3.60 – 3.44 (m, 1H, CH_2), 3.43 – 3.37 (m, 3H, CH_2), 3.25 (t, *J* = 6.7 Hz, 2H, CH_2), 3.15 (t, *J* = 6.7 Hz, 2H, CH_2), 3.10 – 2.96 (m, 2H, CH_2), 2.69 (s, 3H, CH_3), 2.44 (s, 3H, CH_3), 1.70 (s, 3H, CH_3), 1.62 – 1.44 (m, 4H, CH_2), 1.41 – 1.27 (m, 10H, CH_2, $CH(\underline{C}H_3)_2$). ^{13}C NMR (75 MHz, CD_3OD) δ 172.6 (CO), 169.6 (CO), 167.3 (CO), 166.2 (CO), 165.0 (q), 163.2 (q), 158.5 (CO), 157.0 (CO), 155.8 (q), 152.2 (q), 138.1 (ArCl), 138.0 (ArCl), 137.6 (ArCl), 136.4 (ArCl), 134.18 (ArCl), 133.5 (q), 133.3 (q), 133.1 (C-6), 132.01 (q), 131.98 (ArCl), 131.3 (CH-ArCl), 130.7 (CH-ArCl), 130.0 (CH-ArCl), 129.8 (CH-ArCl), 129.1 (CH-ArCl), 129.0 (CH-ArCl), 113.8 (q), 106.4 (C-5), 101.4 (q), 101.2 (C-3), 72.4 ($\underline{C}H(CH_3)_2$), 72.1 (CHN), 70.1 (CHN), 56.2 (OCH_3), 55.3 ($N\underline{C}HCH_2$), 50.6 (CH_2), 50.2 (CH_2), 48.2 (CH_2), 43.1 (CH_2), 40.19 (CH_2), 40.17 ($NCHC\underline{H_2}$), 38.8 (CH_2), 30.4 (CH_2), 30.2 (CH_2), 27.4 (CH_2), 22.5 ($CH(\underline{C}H_3)_2$), 22.4 ($CH(\underline{C}H_3)_2$), 14.4 (CH_3), 13.0 (CH_3), 11.6 (CH_3). LC-MS (high pH) t_R 1.43 min, *m/z* 560.9 $[M/2+H]^+$, 93%. LC-MS (low pH) t_R 1.36 min, *m/z* 561.1 $[M/2+H]^+$, 96%. HRMS (ESI^+) calculated for $C_{57}H_{61}Cl_3N_{10}O_6S$ 1118.3562, found 1118.3555.

2-(4-(4,5-bis(4-chlorophenyl)-2-(2-isopropoxy-4-methoxyphenyl)-4,5-dihydro-1*H*-imidazole-1-carbonyl)-2-oxopiperazin-1-yl)-*N*-(9-(2-((*S*)-4-(4-chlorophenyl)-2,3,9-trimethyl-6*H*-thieno[3,2-*f*][1,2,4]triazolo[4,3-*a*][1,4]diazepin-6-yl)acetamido) nonyl)acetamide (3)

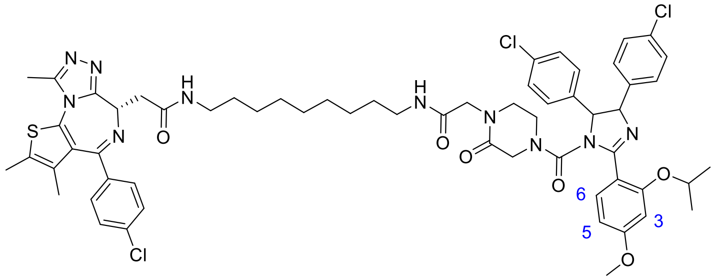

To a solution of **30** (30 mg, 0.04 mmol, 1.0 eq.) in DMF (5 mL) was added **35** (17 mg, 0.04 mmol, 1.1 eq.), HATU (22 mg, 0.06 mmol, 1.5 eq.) and *N*,*N*-diisopropylethylamine (23 µL, 0.14 mmol, 3.5 eq.). The mixture was stirred at rt for 4 h, then quenched with H_2O (20 mL) and extracted with Et_2O (5 × 20 mL). The organics were combined, washed with brine (5 mL), dried over Na_2SO_4, filtered and concentrated *in vacuo*. The crude was purified by reversed-phase chromatography (C18) eluting with 0-98% MeCN/H_2O. The relevant fractions were combined and concentrated *in vacuo*. The material was dissolved in MeOH (3 mL), loaded on a 5 g SCX

HyperSep cartridge, eluted with 7 N NH_3/MeOH and concentrated *in vacuo*. The mixture was further purified by normal phase chromatography (SiO_2) eluting with 0-5% [10% 2N NH_3/MeOH]: DCM. The relevant fractions were combined, concentrated *in vacuo* and purified by reversed-phase chromatography (C18) eluting with 0-98% (MeCN + 0.1% NH_4OH)/H_2O to afford the title compound (14 mg, 0.01 mmol, 32%) as a white powder (mixture of diastereoisomers).

^{1}H NMR (300 MHz, CD_3OD) δ 7.58 (d, J = 8.2 Hz, 1H, H-6), 7.48 – 7.37 (m, 4H, H-Ar), 7.17 – 7.00 (m, 6H, H-Ar), 6.94 (d, J = 8.3 Hz, 2H, H-Ar), 6.69 (d, J = 2.2 Hz, 1H, H-5), 6.66 (s, 1H, H-3), 5.76 (d, J = 10.0 Hz, 1H, CHN), 5.56 (d, J = 10.0 Hz, 1H, CHN), 4.73 (app p, J = 6.0 Hz, 1H, C$\underline{H}$(CH_3)$_2$), 4.63 (dd, J = 9.0, 5.3 Hz, 1H, NC$\underline{H}$$CH_2$), 3.91 (d, J = 16.3 Hz, 1H, CH_2), 3.88 (s, 3H, OCH_3), 3.85 – 3.73 (m, 3H, CH_2), 3.59 – 3.47 (m, 1H, CH_2), 3.48 – 3.32 (m, 3H, CH_2), 3.28 – 3.19 (m, 2H, CH_2), 3.15 (t, J = 7.0 Hz, 2H, CH_2), 3.10 – 2.96 (m, 2H, CH_2), 2.69 (s, 3H, CH_3), 2.44 (s, 3H, CH_3), 1.70 (s, 3H, CH_3), 1.56 (m, 2H, CH_2), 1.48 (m, 2H, CH_2), 1.41 – 1.22 (m, 16H, CH_2, CH(C$\underline{H_3}$)$_2$). ^{13}C NMR (75 MHz, CD_3OD) δ 172.6, 169.6, 167.3, 166.2, 165.0, 163.2, 158.5, 157.0, 155.8, 152.2, 138.1, 138.0, 137.6, 136.4, 134.2, 133.5, 133.2, 133.0, 132.01, 131.99, 131.3, 130.7, 130.0, 129.8, 129.1, 129.0, 113.8, 106.5, 101.4, 101.2, 72.4, 72.1, 70.1, 56.2, 55.3, 50.2, 46.7(under solvent peak), 43.2, 40.5, 38.9, 30.6, 30.5, 30.39, 30.36, 30.3, 28.0, 27.9, 22.5, 22.4, 14.4, 12.9, 11.6. LC-MS (high pH) t_R 1.50 min, *m/z* 1163.2 $[M+H]^+$, 98%. LC-MS (low pH) t_R 1.43 min, *m/z* 1163.4 $[M+H]^+$, 100%. HRMS (ESI^+) calculated for $C_{60}H_{67}Cl_3N_{10}O_6S$ 1160.4031, found 1160.4033.

2-(4-((4*S*,5*R*)-4,5-bis(4-chlorophenyl)-2-(2-isopropoxy-4-methoxyphenyl)-4,5-dihydro-1*H*-imidazole-1-carbonyl)-2-oxopiperazin-1-yl)-*N*-(1-((*S*)-4-(4-chlorophenyl)-2,3,9-trimethyl-6*H*-thieno[3,2-f][1,2,4]triazolo[4,3-a][1,4]diazepin-6-yl)-2-oxo-6,9,12-trioxa-3-azatetradecan-14-yl)acetamide (4)

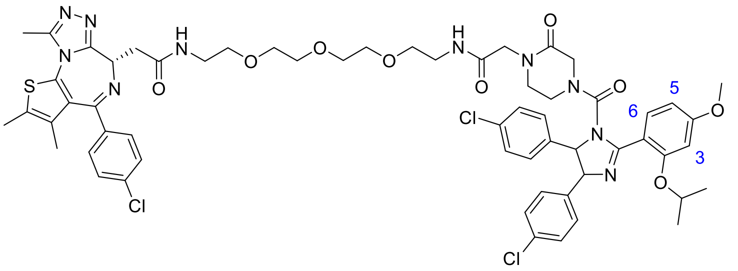

To a solution of **35** (6.4 mg, 0.02 mmol, 1 eq.) in DMF (0.5 mL) was added HATU (7.2 mg, 0.02 mmol, 1.2 eq.) and *N*,*N*-diisopropylethylamine (9.7 µL, 0.06 mmol, 3.5 eq.). After stirring for 10 min at rt, **31** (13 mg, 0.02 mmol, 1 eq.) in DMF (0.5 mL) was added, and the reaction mixture was stirred for 16 h. The mixture was cooled in an ice bath, quenched with H_2O (5 mL) and then extracted with a solution of 10% MeOH in DCM (3 × 10 mL). The combined organics were washed with brine (10 mL), dried over $MgSO_4$, filtered and concentrated *in vacuo*. The crude was purified by column chromatography on a 4 g SiO_2 column, eluting with 0-10% [MeOH + 10% 2 M NH_3/ MeOH]: DCM. The relevant fractions were combined and concentrated *in vacuo*. The residue was further purified by reverse phase chromatography (4 g C18 column), eluting with 5-95% MeCN: H_2O. The appropriate fractions were combined and concentrated *in vacuo* to afford the title compound (2.7 mg, 0.002 mmol, 14%) as a white solid (mixture of diastereoisomers).

^{1}H NMR (300 MHz, CD_3OD) δ 7.57 (d, J = 8.7 Hz, 1H, H-6), 7.51 – 7.36 (m, 4H, H-Ar), 7.18 – 6.99 (m, 6H, H-Ar), 6.94 (d, J = 8.4 Hz, 2H, H-Ar), 6.68 (d, J = 1.9 Hz, 1H, H-5), 6.65 (s, 1H, H-3), 5.76 (d, J = 10.1 Hz, 1H, CHN), 5.56 (d, J = 10.1 Hz, 1H, CHN), 4.73 (app p, J = 6.0 Hz, 1H, C$\underline{H}$(CH_3)$_2$), 4.63 (dd, J = 8.8, 5.3 Hz, 1H, NC$\underline{H}$$CH_2$), 3.94 (d, J = 16.2 Hz, 1H, CH_2), 3.87 (s, 3H, OCH_3), 3.85 – 3.76 (m, 3H, CH_2), 3.68 – 3.57 (m, 10H, OCH_2), 3.51 (app t, J = 5.4 Hz, 3H, CH_2), 3.48 – 3.39 (m, 4H, CH_2), 3.37 – 3.34 (m, 3H, CH_2), 3.02 (app q, J = 5.2 Hz, 2H, CH_2), 2.69 (d, J = 1.5 Hz, 3H, CH_3), 2.44 (s, 3H, CH_3), 1.70 (s, 3H, CH_3), 1.36 (dd, J = 10.1, 6.0 Hz, 6H, CH(C$\underline{H_3}$)$_2$). ^{13}C NMR (75 MHz, CD_3OD) δ 172.9 (CO), 169.8 (CO), 167.3 (CO), 165.0 (CO), 162.2 (q), 159.8 (q), 158.5 (CO), 157.0 (CO), 140.1 (q), 138.2 (ArCl), 138.0 (ArCl), 137.7 (ArCl), 137.6 (ArCl), 136.5 (ArCl), 134.2 (ArCl), 133.5 (q), 133.2 (q), 133.0 (C-6), 132.0 (q), 131.4 (CH-ArCl), 130.7 (CH-ArCl), 130.0 (CH-ArCl), 129.8 (CH-ArCl), 129.1 (CH-ArCl), 129.0 (CH-ArCl), 113.8 (q), 105.2 (C-5), 101.4 (q), 101.2 (C-3), 72.1 ($\underline{C}$H(CH_3)$_2$), 71.7 (OCH_2), 71.6 (OCH_2), 71.3 (OCH_2), 71.2 (OCH_2), 70.6 (OCH_2), 70.4 (OCH_2), 70.1 (CHN), 56.2 (OCH_3), 55.2 (CH, NC$\underline{H}$$CH_2$), 50.5 ($CH_2$), 50.2 ($CH_2$), 50.1 ($CH_2$, NCHC$\underline{H_2}$), 48.3 ($CH_2$), 40.6 ($CH_2$), 40.4 ($CH_2$), 38.8 ($CH_2$), 22.5 ($CH_3$, CH($\underline{C}H_3$)$_2$), 22.4 ($CH_3$, CH($\underline{C}H_3$)$_2$), 14.4 ($CH_3$), 12.9 ($CH_3$), 11.6 ($CH_3$). LC-MS (high pH) t_R 1.39 min, *m/z* 599.5 $[M/2+H]^+$, 96%. LC-MS (low pH) t_R 1.31 min, *m/z* 598.9 $[M/2+H]^+$, 97%. HRMS (ESI^+) calculated for $C_{59}H_{65}Cl_3N_{10}O_9S$ 1194.3722, found 1194.3715.

2-(4-((4*S*,5*R*)-4,5-bis(4-chlorophenyl)-2-(2-isopropoxy-4-methoxyphenyl)-4,5-dihydro-1*H*-imidazole-1-carbonyl)-2-oxopiperazin-1-yl)-*N*-(1-((*S*)-4-(4-chlorophenyl)-2,3,9-trimethyl-6*H*-thieno[3,2-f][1,2,4]triazolo[4,3-a][1,4]diazepin-6-yl)-2-oxo-6,9,12,15-tetraoxa-3-azaheptadecan-17-yl)acetamide (5)

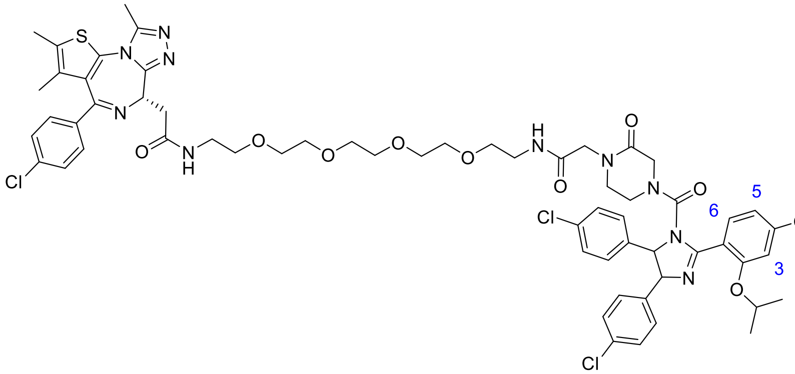

To a solution of **35** (12 mg, 0.03 mmol, 1 eq.) in DMF (0.5 mL) was added HATU (14 mg, 0.04 mmol, 1.2 eq.) and *N*,*N*-diisopropylethylamine (19 µL, 0.11 mmol, 3.5 eq.). After stirring for 1 h at rt, **32** (28 mg, 0.03 mmol, 1 eq.) in DMF (0.5 mL) was added, and the reaction mixture was stirred for 16 h. The mixture was cooled in an ice bath, quenched with H_2O (5 mL) and then extracted subsequently with EtOAc (2 × 10 mL) and a solution of 10% MeOH in DCM (2 × 10 mL). The combined organics were washed with brine (10 mL), dried over $MgSO_4$, filtered and concentrated *in vacuo*. The crude was purified by column chromatography on a 4 g SiO_2 column, eluting with 0-20% [10% NH_4OH/ MeOH]: DCM. The relevant fractions were combined and concentrated *in vacuo*. The residue was then purified by column chromatography on a 4 g SiO_2 column, eluting with 0-10% [MeOH + 10% 2 M NH_3/ MeOH]: DCM. The relevant fractions were combined, concentrated *in vacuo* and further purified by reverse phase chromatography (4 g C18 column), eluting with 5-95% MeCN: H_2O. The appropriate fractions were combined and the solvent removed *in vacuo* to afford the title compound (13 mg, 0.01 mmol, 34%) as a white solid (mixture of diastereoisomers).

^{1}H NMR (300 MHz, CD_3OD) δ 7.58 (d, *J* = 8.3 Hz, 1H, H-6), 7.43 (app q, *J* = 8.5 Hz, 4H, H-Ar), 7.18 – 6.98 (m, 6H, H-Ar), 6.94 (d, *J* = 8.2 Hz, 2H, H-Ar), 6.68 (d, *J* = 2.2 Hz, 1H, H-5), 6.65 (s, 1H, H-3), 5.76 (d, *J* = 10.0 Hz, 1H, CHN), 5.56 (d, *J* = 10.0 Hz, 1H, CHN), 4.72 (q, *J* = 6.0 Hz, 1H, C<u>H</u>(CH_3)$_2$), 4.63 (dd, *J* = 8.9, 5.2 Hz, 1H, NC<u>H</u>CH_2), 3.94 (d, *J* = 16.3 Hz, 1H, CH_2), 3.87 (s, 3H, OCH_3), 3.85 – 3.76 (m, 3H, CH_2), 3.68 – 3.53 (m, 14H, OCH_2), 3.52 – 3.39 (m, 6H, OCH_2), 3.38 – 3.33 (m, 4H, CH_2), 3.07 – 2.99 (m, 2H, CH_2), 2.69 (s, 3H, CH_3), 2.44 (s, 3H, CH_3), 1.70 (s, 3H, CH_3), 1.36 (dd, *J* = 9.8, 6.0 Hz, 6H, CH(C<u>H</u>$_3$)$_2$). ^{13}C NMR (75 MHz, CD_3OD) δ 172.9 (CO), 169.8 (CO), 167.3 (CO), 166.1 (CO), 165.0 (q), 163.2 (q), 158.5 (CO), 157.0 (CO), 155.8 (q), 152.2 (q), 138.1 (ArCl), 138.0 (ArCl), 137.6 (ArCl), 136.5 (ArCl), 134.2 (ArCl), 133.5 (ArCl), 133.2 (q), 133.0 (C-6), 132.02 (q), 131.97 (q), 131.4 (CH-ArCl), 130.7 (CH-ArCl), 130.0 (CH-ArCl), 129.8 (CH-ArCl), 129.1 (CH-ArCl), 129.0 (CH-ArCl), 113.8 (q), 106.5 (C-5), 101.4 (q), 101.2 (C-3), 72.4 (C<u>H</u>(CH_3)$_2$), 71.6 (OCH_2), 71.54 (OCH_2), 71.50 (OCH_2), 71.3 (OCH_2), 71.2 (OCH_2), 70.6 (OCH_2), 70.4 (OCH_2), 70.1 (CHN), 56.2 (OCH_3), 55.2 (N<u>C</u>HCH_2), 50.5 (CH_2), 50.1 (CH_2), 48.1 (CH_2), 43.2 (CH_2), 40.6 (CH_2), 40.4 (CH_2), 38.8 (CH_2), 22.5 (CH(<u>C</u>H$_3$)$_2$), 22.4 (CH(<u>C</u>H$_3$)$_2$), 14.4 (CH_3), 13.0 (CH_3), 11.6 (CH_3). LC-MS (high pH) t_R 1.39 min, *m/z* 621.5 $[M/2+H]^+$, 97%. LC-MS (low pH) t_R 1.31 min, *m/z* 620.9 $[M/2+H]^+$, 96%. HRMS (ESI^+) calculated for $C_{61}H_{69}Cl_3N_{10}O_{10}S$ 1238.3984, found 1238.3977.

2-(4-(4,5-bis(4-chlorophenyl)-2-(2-isopropoxy-4-methoxyphenyl)-4,5-dihydro-1*H*-imidazole-1-carbonyl)-2-oxopiperazin-1-yl)-*N*-(1-((*S*)-4-(4-chlorophenyl)-2,3,9-trimethyl-6*H*-thieno[3,2-*f*][1,2,4]triazolo[4,3-*a*][1,4]diazepin-6-yl)-2-oxo-6,9,12,15,18-pentaoxa-3-azaicosan-20-yl)acetamide (6)

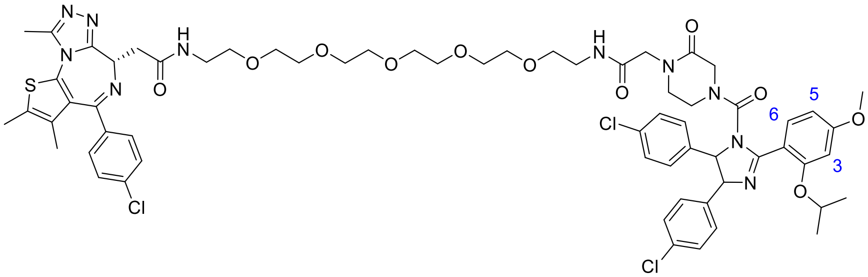

To a solution of **33** (34 mg, 0.04 mmol, 1.0 eq.) in DMF (5 mL) were added **35** (17 mg, 0.04 mmol, 1.1 eq.), HATU (22 mg, 0.06 mmol, 1.5 eq.) and DIPEA (23 µL, 0.14 mmol, 3.5 eq.). After stirring at rt for 18 h, the mixture was quenched with H_2O (20 mL) and extracted with Et_2O (5 × 20 mL). The organics were washed with brine (5 mL), dried over Na_2SO_4, concentrated *in vacuo* and purified by column chromatography on a 4.3 g C18 column, eluting with 0–98% MeCN/H_2O. The relevant fractions were concentrated *in vacuo*, redissolved in MeOH (3 mL), loaded on a 5 g SCX HyperSep™ cartridge, eluted with 7 N NH_3/MeOH and

concentrated *in vacuo* again. The mixture was purified on a 4 g SiO_2 column, eluting with 0–5% [10% 2 N NH_3/MeOH]/DCM, then again on a 4.3 g C18 column, eluting with 0–98% (MeCN + 0.1% NH_4OH)/H_2O. The relevant fractions were concentrated *in vacuo* to afford the title compound (21 mg, 0.02 mmol, 43%) as a white powder (mixture of diastereoisomers).

^{1}H NMR (300 MHz, CD_3OD) δ 7.58 (d, *J* = 8.8 Hz, 1H, H-6), 7.50 – 7.38 (m, 4H, H-Ar), 7.17 – 7.00 (m, 6H, H-Ar), 6.94 (d, *J* = 8.4 Hz, 2H, H-Ar), 6.68 (d, *J* = 2.3 Hz, 1H, H-5), 6.65 (s, 1H, H-3), 5.76 (d, *J* = 10.0 Hz, 1H, CHN), 5.56 (d, *J* = 10.0 Hz, 1H, CHN), 4.72 (app q, *J* = 6.0 Hz, 1H, C$\underline{H}$(CH_3)$_2$), 4.63 (dd, *J* = 9.0, 5.2 Hz, 1H, NC$\underline{H}$$CH_2$), 3.95 (d, *J* = 16.3 Hz, 1H, CH_2), 3.87 (s, 3H, OCH_3), 3.86 – 3.76 (m, 3H, CH_2), 3.69 – 3.54 (m, 18H, OCH_2), 3.54 – 3.39 (m, 6H, CH_2), 3.38 – 3.33 (m, 4H, CH_2), 3.04 (q, *J* = 5.0 Hz, 2H, CH_2), 2.69 (s, 3H, CH_3), 2.44 (s, 3H, CH_3), 1.70 (s, 3H, CH_3), 1.36 (dd, *J* = 9.9, 6.0 Hz, 6H, CH($\underline{CH_3}$)$_2$). ^{13}C NMR (75 MHz, CD_3OD) δ 172.9, 169.8, 167.3, 166.1, 165.0, 163.2, 158.5, 157.0, 155.8, 152.1, 138.2, 137.9, 137.6, 136.5, 134.2, 133.5, 133.2, 133.0, 132.0, 132.0, 131.4, 130.7, 130.0, 129.8, 129.1, 129.0, 113.8, 106.5, 101.4, 101.2, 72.4, 72.1, 71.6, 71.5, 71.4, 71.2, 70.6, 70.4, 70.1, 56.2, 55.2, 50.5, 50.1, 48.2, 43.2, 40.6, 40.4, 38.8, 22.5, 22.4, 14.4, 12.9, 11.6. LC-MS (high pH) t_R 1.39 min, *m/z* 1285.0 [M]$^+$, 92%. LC-MS (low pH) t_R 1.32 min, *m/z* 1285.0 [M]$^+$, 94%. HRMS (ESI$^+$) calculated for $C_{63}H_{73}Cl_3N_{10}O_{11}S$ 1282.4247, found 1282.4247.

References

1. Freysoldt, T. H. E. Cyclic nitrones - synthesis and applications. Technical University of Berlin, 2006.
2. Frank, W.; Karola, R.-B., Introduction of Substituents on the 2-Oxo-piperazine Skeleton by [3+2] Cycloaddition and Subsequent Transformation. *Z. Naturforsch. B* **2006**, 61 (4), 431-436.
3. *BE Pat.*, WO2015/150555, 2015.
4. Kaluđerović, G. N.; Pantelić, N.; Eichhorn, T.; Bette, M.; Wagner, C.; Zmejkovski, B. B.; Schmidt, H., Platinum(II) complexes with R2edda ligands (R=Me, Et, n-Pr; edda=ethylenediamine-N,N′-diacetate): Synthesis and characterization. *Polyhedron* **2014**, 80, 53-59.
5. Wilk, B. K.; Mwisiya, N.; Helom, J. L., Solving a Scale-Up Problem in the O-Alkylation of Isovanillin Under Phase-Transfer Catalysis Conditions. *Org. Process Res. Dev.* **2008**, 12 (4), 785-786.
6. Van Veldhuizen, J. J.; Gillingham, D. G.; Garber, S. B.; Kataoka, O.; Hoveyda, A. H., Chiral Ru-Based Complexes for Asymmetric Olefin Metathesis: Enhancement of Catalyst Activity through Steric and Electronic Modifications. *J. Am. Chem. Soc.* **2003**, 125 (41), 12502-12508.
7. Hu, C.; Li, X.; Wang, W.; Zhang, L.; Tao, L.; Dong, X.; Sheng, R.; Yang, B.; Hu, Y., Design, synthesis, and biological evaluation of imidazoline derivatives as p53-MDM2 binding inhibitors. *Bioorg. Med. Chem.* **2011**, 19 (18), 5454-61.
8. *CN Pat.*, CN108610333A, 2018.
9. Zengerle, M.; Chan, K.-H.; Ciulli, A., Selective Small Molecule Induced Degradation of the BET Bromodomain Protein BRD4. *ACS Chem. Biol.* **2015**, 10 (8), 1770-1777.
10. Kim, S. A.; Go, A.; Jo, S. H.; Park, S. J.; Jeon, Y. U.; Kim, J. E.; Lee, H. K.; Park, C. H.; Lee, C. O.; Park, S. G.; Kim, P.; Park, B. C.; Cho, S. Y.; Kim, S.; Ha, J. D.; Kim, J. H.; Hwang, J. Y., A novel cereblon modulator for targeted protein degradation. Eur. J. Med. Chem. **2019**, 166, 65-74.
11. *CH Pat.*, WO2006/090244, 2006.
12. *US Pat.,* WO2016210215 (A1), 2016.
13. *US Pat.*, WO2017/181061 A1, 2017.

3. Biology

3.1. Cell line and culture conditions

MIA PaCa-2 cells (RRID: CVCL_0428) were obtained from ATCC (CRL-1420) and cultured in Dulbecco's Modified Eagle Medium (DMEM, Sigma, D6546) supplemented with 4 mM L-Glutamine, 10% Fetal Bovine Serum (FBS, Sigma, F7524), 2.5% horse serum (HS, ATCC, 30-2040), 1% GlutaMAX (ThermoFisher, 35050), 2.5% 25 mM HEPES (Sigma, H0887). The cells were maintained at 37˚C, 5% CO_2, 85% RH, routinely tested for mycoplasma contamination and authenticated by short tandem repeat (STR) profiling.

3.2. Immunoblotting

MIA PaCa-2 cells were plated at 1.8×10^5 cells/mL in 24 well plates (1 mL/well) and allowed to attach overnight. The cells were treated with the corresponding compound and incubated for 20–48 h.

After the corresponding incubation period, the plates were then placed on ice and the media was taken out in a 2 mL Eppendorf tube. The cells were washed with 2 × 0.5 mL phosphate buffered saline (PBS), and the PBS washes were collected in the same 2 mL Eppendorf tube. The tubes were centrifuged at 1000 rpm for 5 min. The media was removed, and the pellet was resuspended in 50 µL lysis buffer containing RIPA-buffer (Sigma, R0278) supplemented with Halt^{TM} protease inhibitor cocktail (100×) (Thermo Scientific, 1862209), Halt^{TM} phosphatase inhibitor cocktail (100×) (Thermo Scientific, 1861277) and 0.5 M EDTA solution (100×) (Thermo Scientific, 1861274) and transferred to the cells in the corresponding wells. The plate was placed on ice for 15 min. The cells were scraped with cell scrapers, and the mixture was transferred into cold 1.5 mL Eppendorf tubes. The lysates were centrifuged at 14000 rpm for 10 min at 4°C. The supernatant was transferred to clean tubes and stored at −20 °C until needed. Protein concentrations of the supernatants were quantified using a Pierce BCA Protein Assay (23225). The protein concentration was normalized, and the samples were diluted in NuPage LSD Sample buffer (4×) (Invitrogen, 1887691) and NuPage Sample reducing agent (10×) (Invitrogen, 1895560) and denatured at 95°C for 5 min. An equal amount of protein samples (20- 33 µg) were separated using a gradient (4–20%) Criterion TGX precast gel (Bio-Rad, 5671094) and transferred onto a 0.2 µM pore size polyvinylidene difluoride (PVDF) blotting membrane (Trans- Blot Turbo Bio-Rad, 1704157) using trans-blot electrophoretic transfer cell (Bio-Rad). The membranes were then blocked in 5% w/v bovine serum albumin (BSA, Sigma, A4503) in PBST (PBS + 0.1% Tween 20) for 30-60 min before probing with the indicated primary antibody in BSA (5% w/v BSA in PBST) overnight at 4 °C under smooth agitation. The membranes were washed with PBST (3 × 5 min), then probed with the corresponding secondary antibody in BSA (5% w/v BSA in PBST) for 1 h at rt under smooth agitation, then washed again with PBST (3 × 5 min). The membranes were imaged using GeneSys (Syngene) using Western Lightning Plus-ECL (PerkinElmer, NEL10300IEA).

Antibodies used and their concentrations: BRD4 (Abcam, ab128874, 1:1000, RRID: AB_11145462), Vinculin (Sigma, V9131, 1:1000, RRID: AB_477629), c-Myc (Abcam, ab32072, 1:1000, RRID: AB_731658), GAPDH (Abcam, ab9485, 1:2500, RRID: AB_307275), anti-rabbit HRP-linked antibody (Cell Signaling, 7074S, 1:2000, RRID: AB_2099233), anti-mouse HRP-linked antibody (Cell Signaling, 7076S, 1:2000, RRID: AB_330924). Switched from anti-GAPDH to the anti-Vinculin antibody intermittently for better and clearer data.

4. Characterization data novel compounds

^{1}H NMR Compound **3**

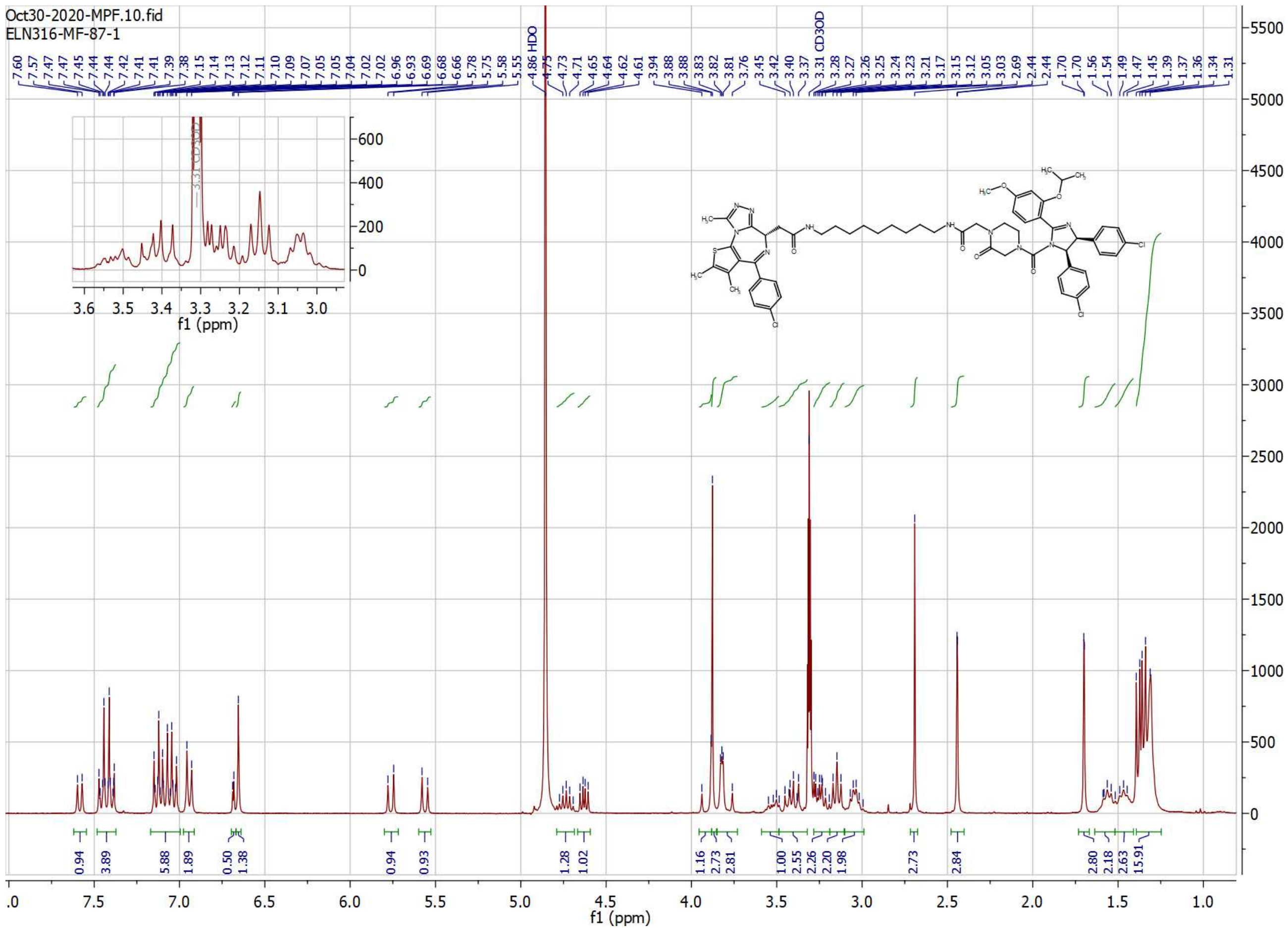

^{13}C NMR Compound **3**:

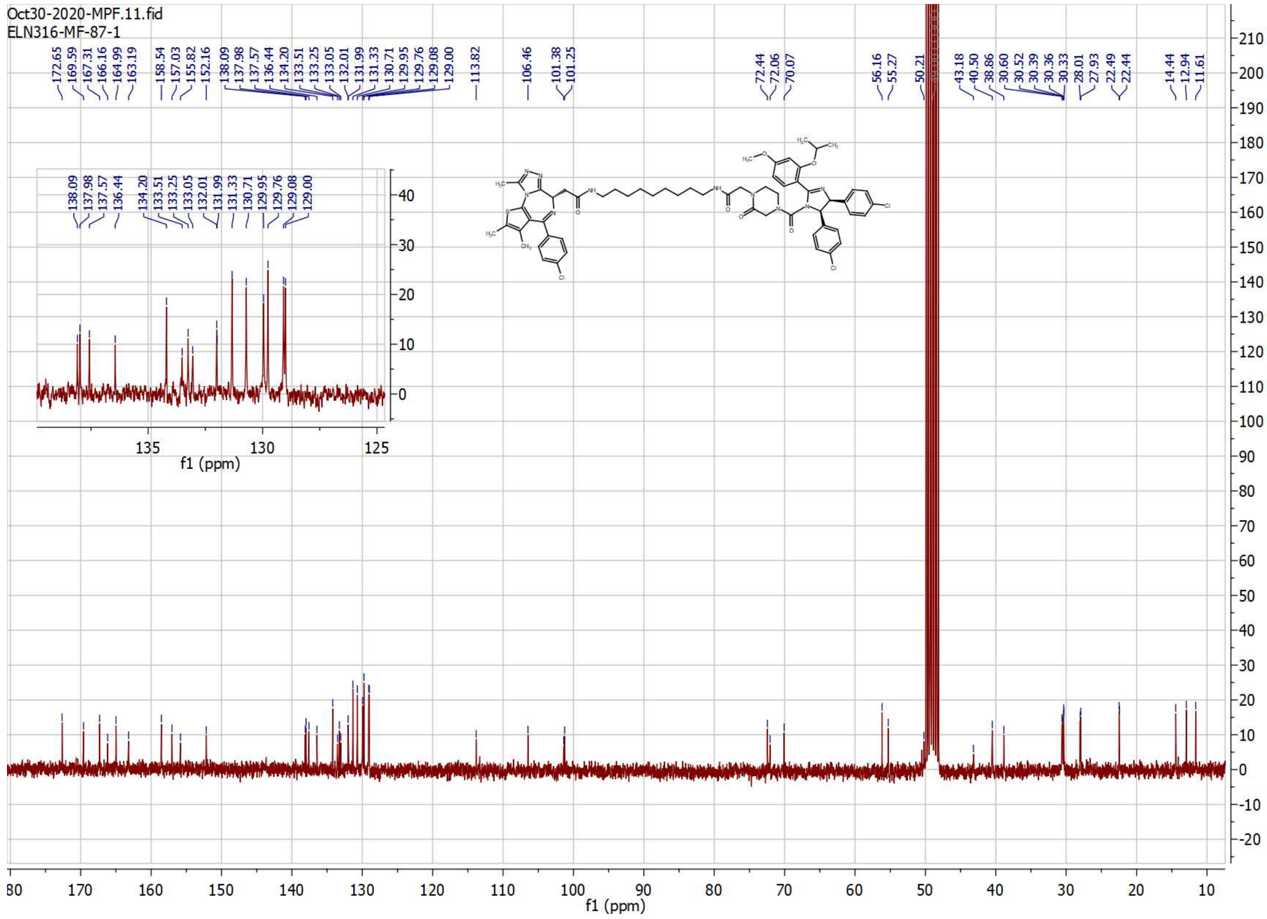

LC–MS (high pH) Compound **3**:

Openlynx Report - mficu-A- Page 1

Sample: 1 Vial:1:30
SampleID and Description:ELN316-MF-87-1-1 File:mficu-A-473-1
Date:30-Oct-2020 Time:15:53:19
Method:C:\MassLynx\High_pH_HIGH_mass_2min.olp

Printed: Fri Oct 30 15:57:14 2020

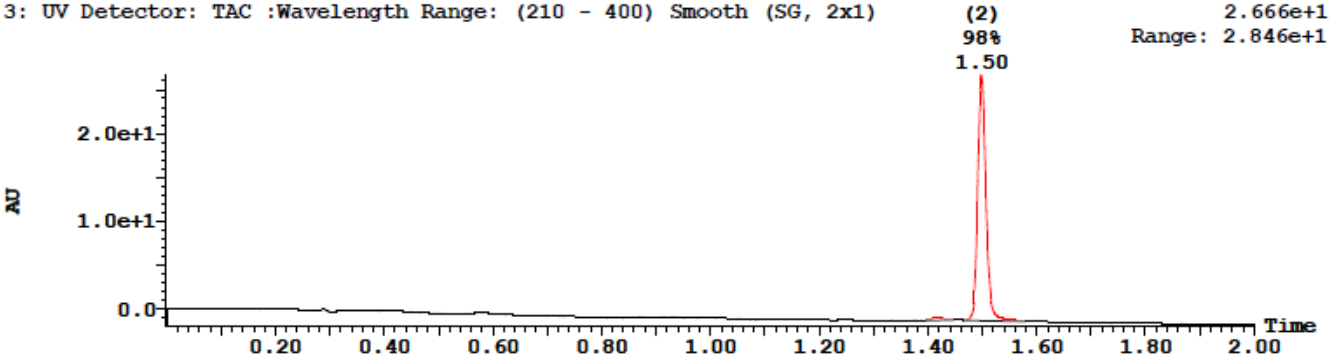

Peak Number	Compound	Time	Area %Total	Mass Found
1		1.42	1.96	Not Found
2		1.50	98.04	Not Found

Peak ID	Compound	Time	Mass Found
1		1.42	Not Found

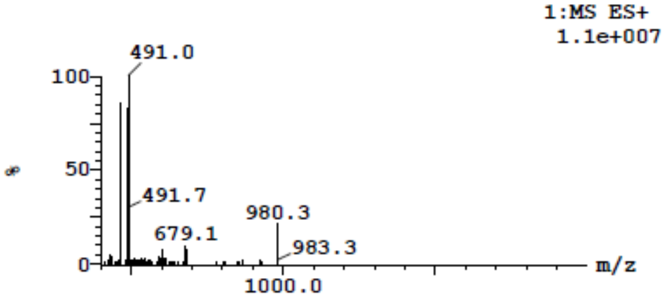

Peak ID	Compound	Time	Mass Found
1		1.42	Not Found

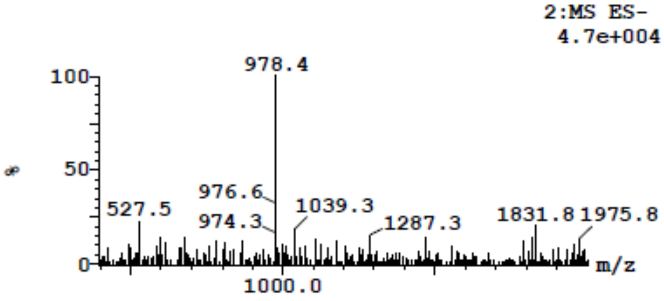

Peak ID	Compound	Time	Mass Found
2		1.50	Not Found

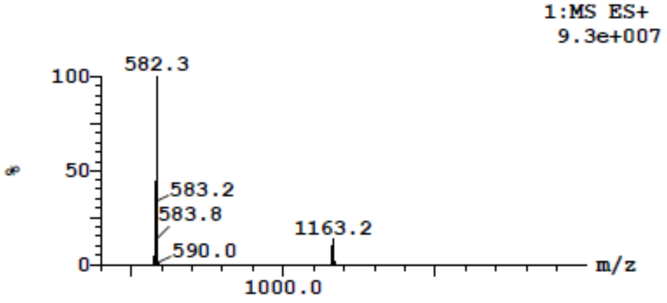

Peak ID	Compound	Time	Mass Found
2		1.50	Not Found

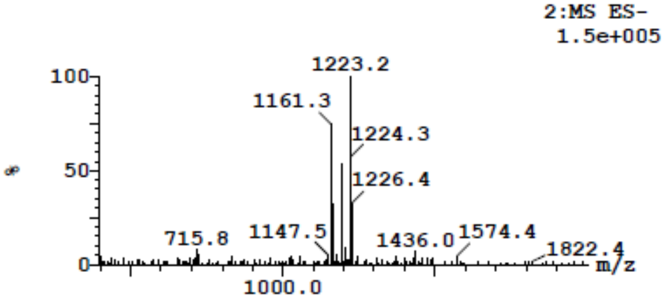

LC–MS (low pH) Compound **3**:

Openlynx Report - mficu-I- Page 1

Sample: 1 Vial:1:12 SampleID and Description:ELN316-MF-87-1

File:mficu-I-212-1 Date:30-Oct-2020 Time:16:27:32

Method:C:\MassLynx\Low_pH_HIGH_mass_2min.olp

Printed: Fri Oct 30 16:33:51 2020

3: UV Detector: TAC: Wavelength Range: (210 - 400) Smooth (SG, 2x1) 4.614e+1

Range: 5.163e+1

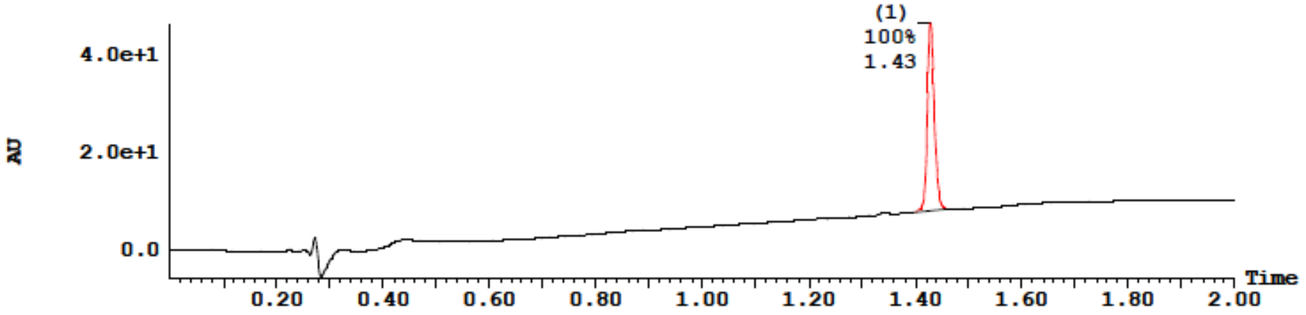

Peak Number	Compound	Time	Area %Total	Mass Found
1		1.43	100.00	Not Found

Peak ID	Compound	Time	Mass Found
1		1.43	Not Found

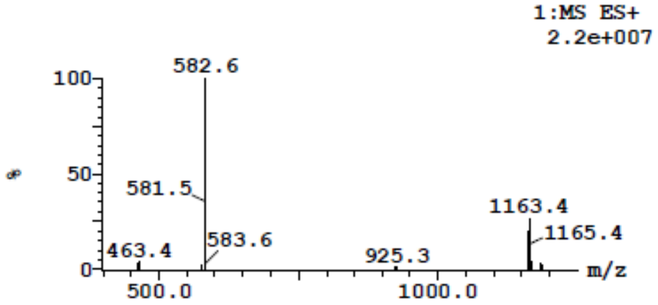

Peak ID	Compound	Time	Mass Found
1		1.43	Not Found

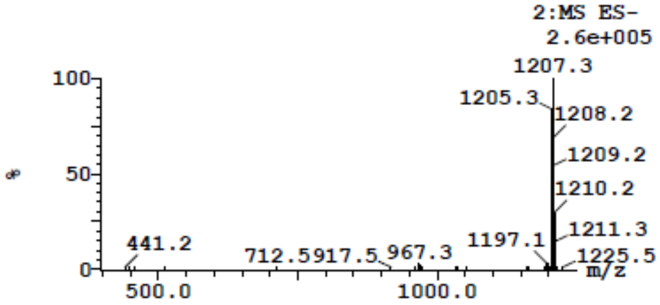

HRMS Compound **3**:

Spectrum Plot Report

Agilent Trusted Answers

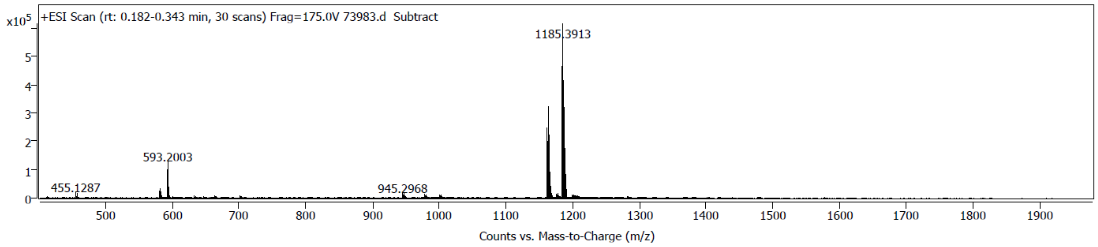

Spectrum Plot Report

Agilent Trusted Answers

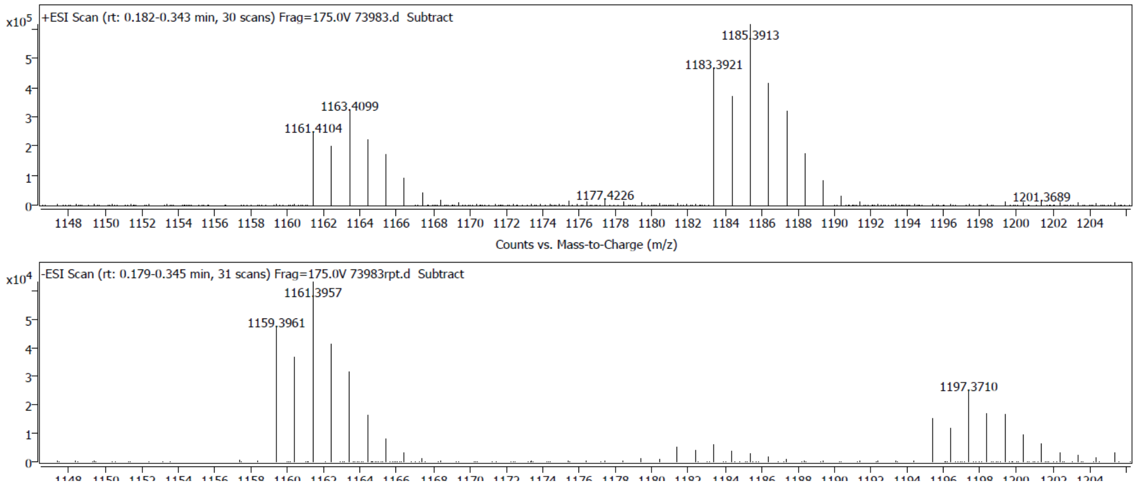

Target Screening Report

Agilent Trusted Answers

Compound Spectra (overlaid)

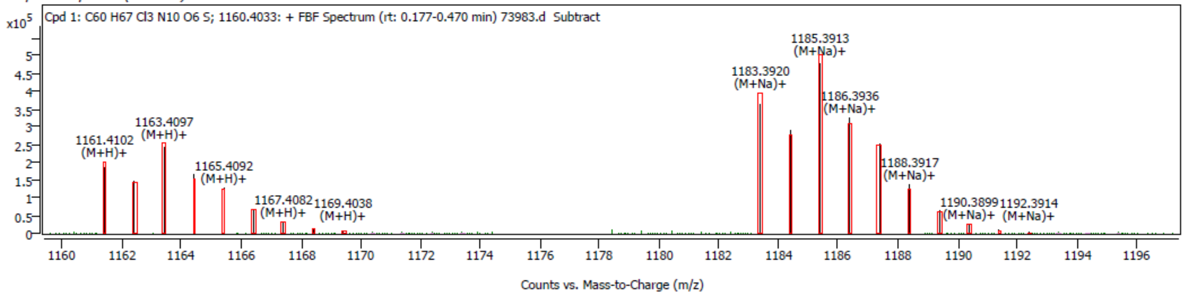

Compound ID Table

Name	Formula	Species	RT	RT Diff	Mass	CAS	ID Source	Score	Score (Lib)	Score (Tgt)
	C60 H67 Cl3 N10 O6 S	(M+H)+ (M+Na)+	0.254		1160.4033		FBF	97.67		97.67

MassHunter Qual 10.0
(End of Report)

^{1}H NMR Compound **1**:

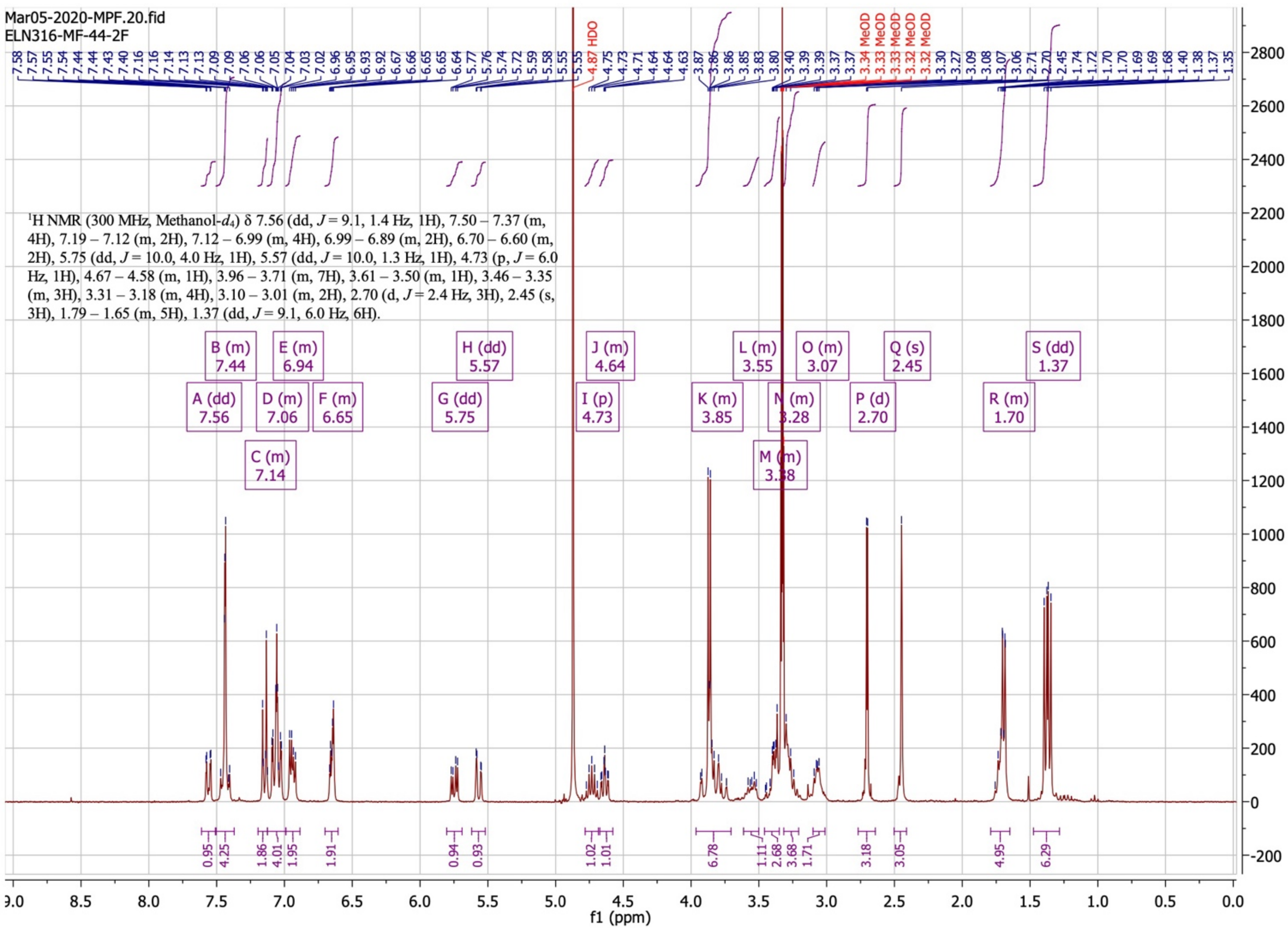

^{13}C NMR Compound **1**:

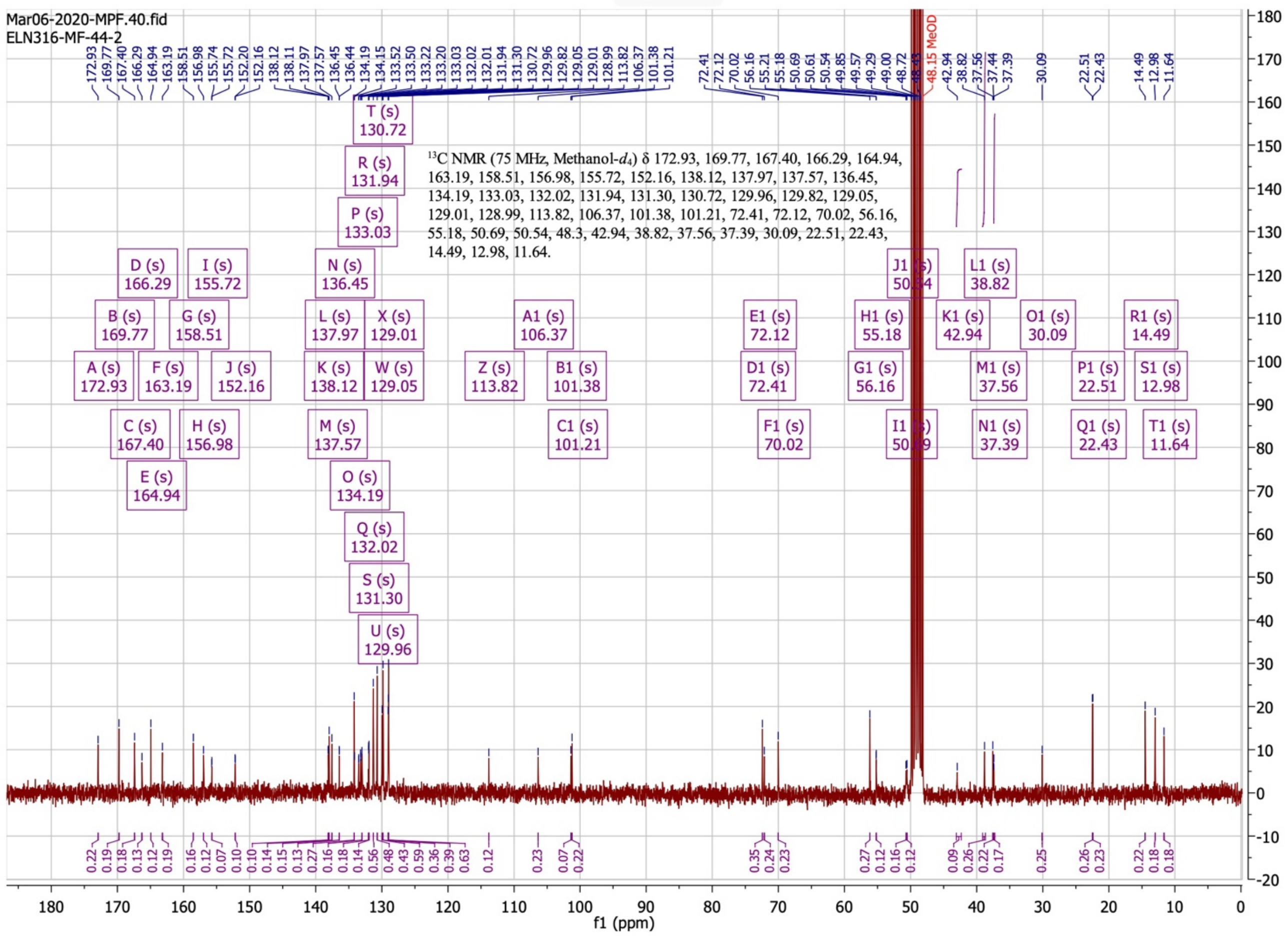

LC–MS (high pH) Compound **1**:

Openlynx Report - mficu-A- Page 1

Sample: 1 Vial:1:39

SampleID and Description:ELN316-MF-44-2-1 File:mficu-A-228-1

Date:10-Mar-2020 Time:17:14:23 Method:C:\MassLynx\High_pH_2min.olp

Printed: Tue Mar 10 17:21:00 2020

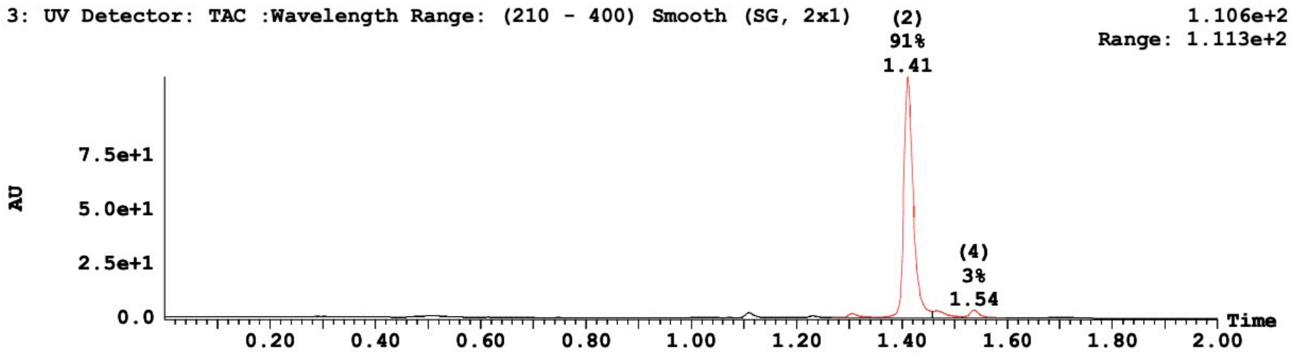

Peak Number	Compound	Time	Area %Total	Mass Found
1		1.31	1.85	Not Found
2		1.41	91.36	Not Found
3		1.47	3.76	Not Found
4		1.54	3.03	Not Found

Peak ID	Compound	Time	Mass Found
1		1.31	Not Found

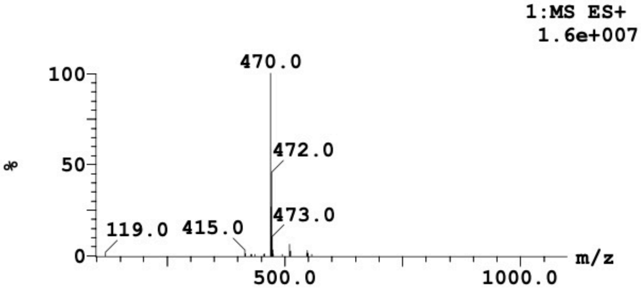

Peak ID	Compound	Time	Mass Found
1		1.31	Not Found

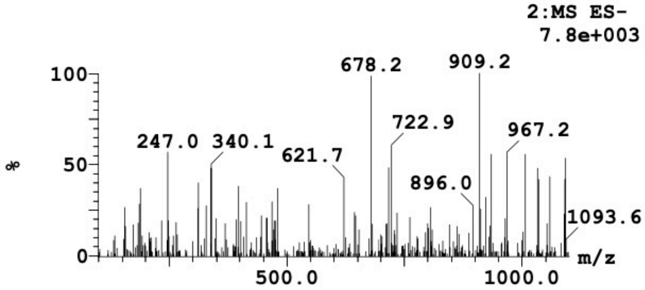

Peak ID	Compound	Time	Mass Found
2		1.41	Not Found

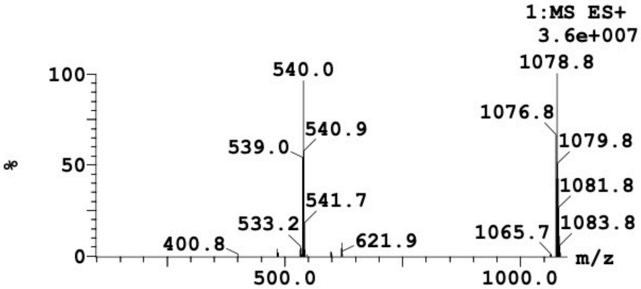

Peak ID	Compound	Time	Mass Found
2		1.41	Not Found

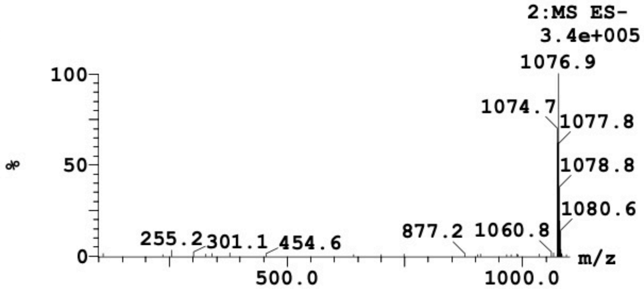

LC–MS (low pH) Compound **1**:

Openlynx Report - mficu-A- Page 1

Sample: 1 Vial:1:39

SampleID and Description:ELN316-MF-44-2-2 File:mficu-A-229-1

Date:10-Mar-2020 Time:17:21:01 Method:C:\MassLynx\Low_pH_2min.olp

Printed: Tue Mar 10 17:27:07 2020

3: UV Detector: TAC :Wavelength Range: (210 - 400) Smooth (SG, 2x1)

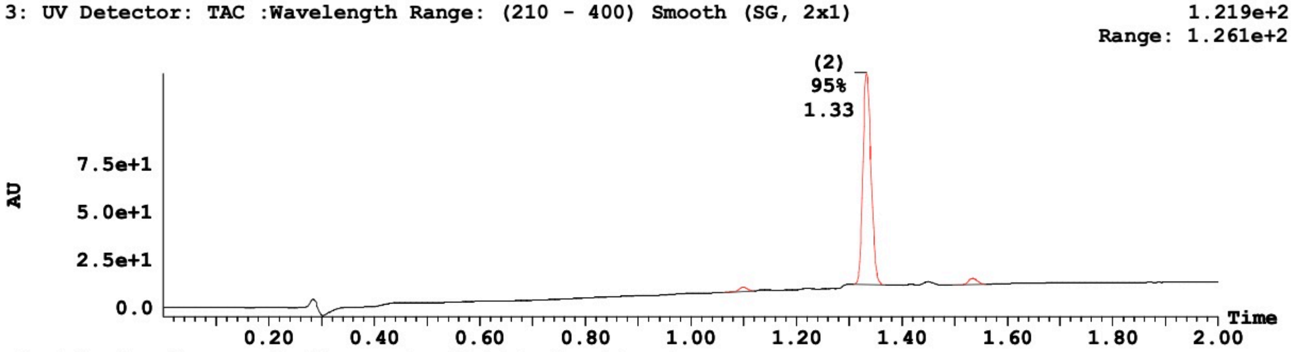

Peak Number	Compound	Time	Area %Total	Mass Found
1		1.10	2.17	Not Found
2		1.33	94.91	Not Found
3		1.53	2.92	Not Found

Peak ID	Compound	Time	Mass Found
1		1.10	Not Found

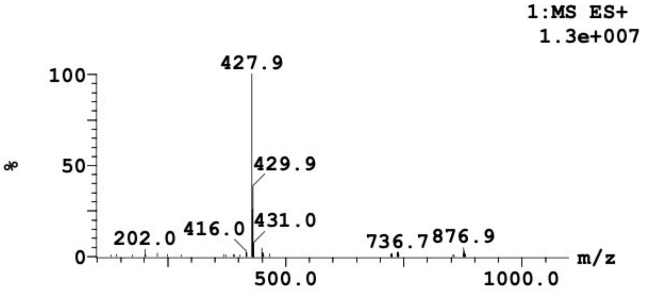

Peak ID	Compound	Time	Mass Found
1		1.10	Not Found

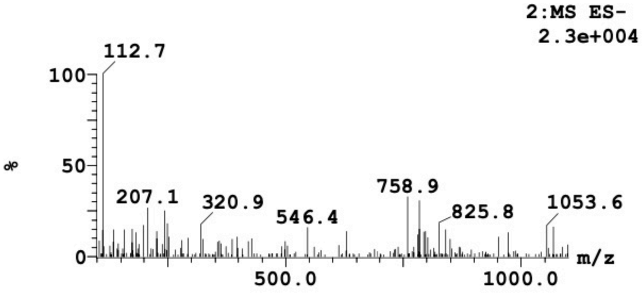

Peak ID	Compound	Time	Mass Found
2		1.33	Not Found

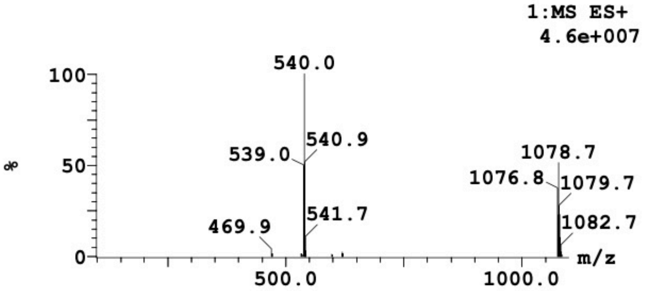

Peak ID	Compound	Time	Mass Found
2		1.33	Not Found

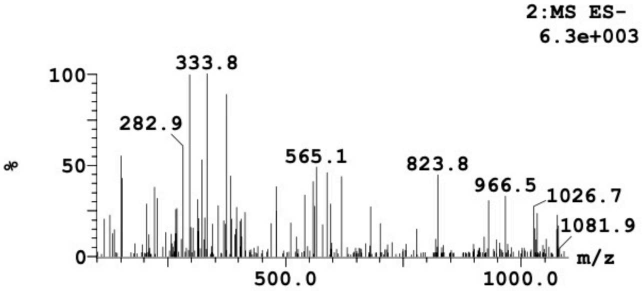

HRMS Compound **1**:

Spectrum Plot Report

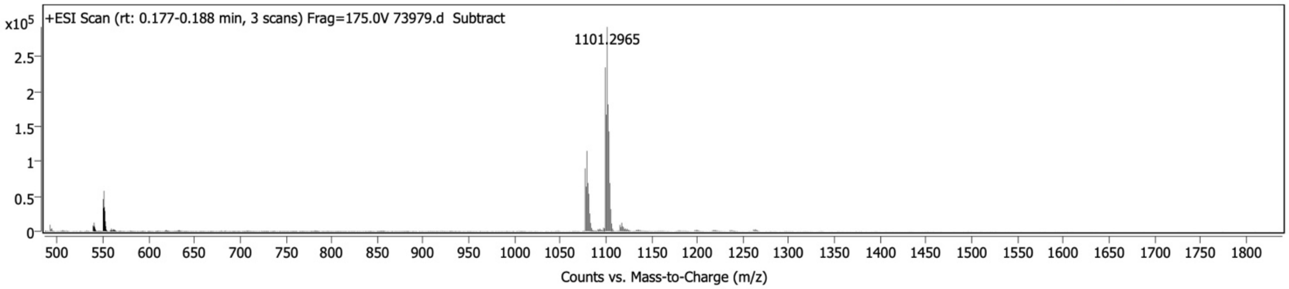

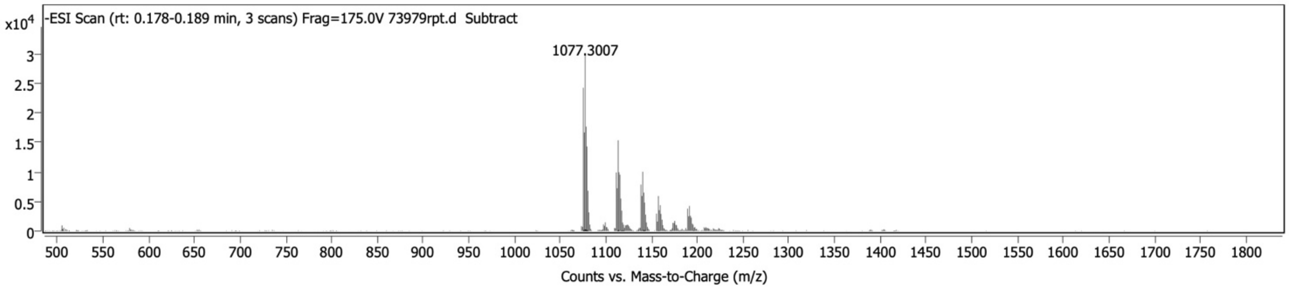

Spectrum Plot Report

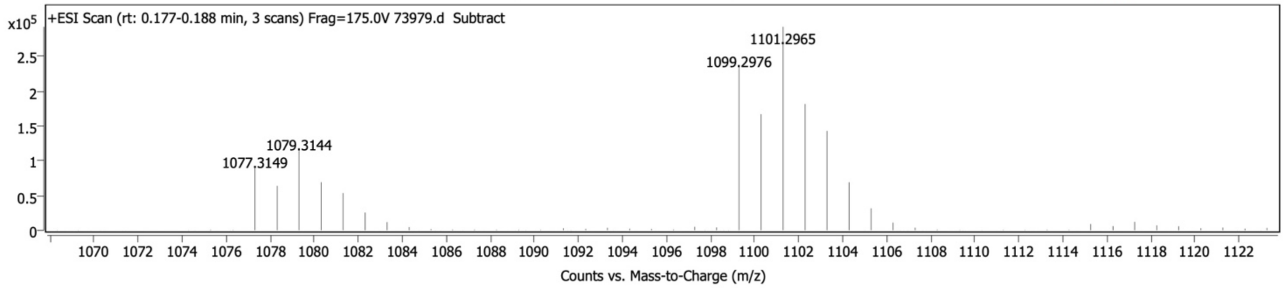

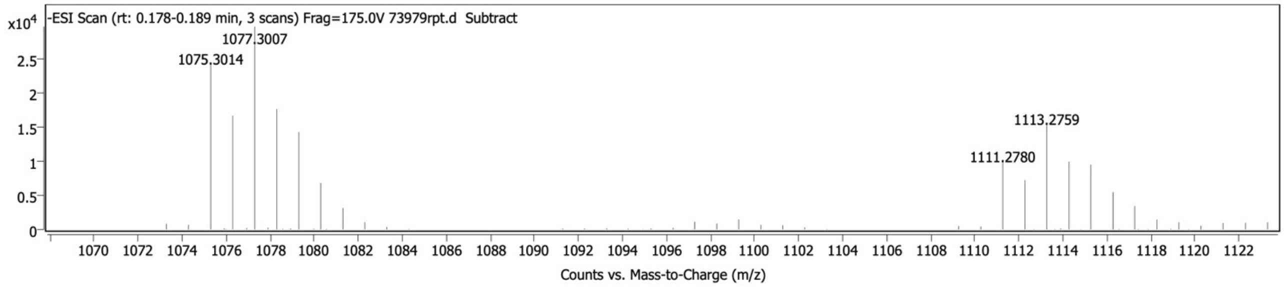

Target Screening Report

Compound Spectra (overlaid)

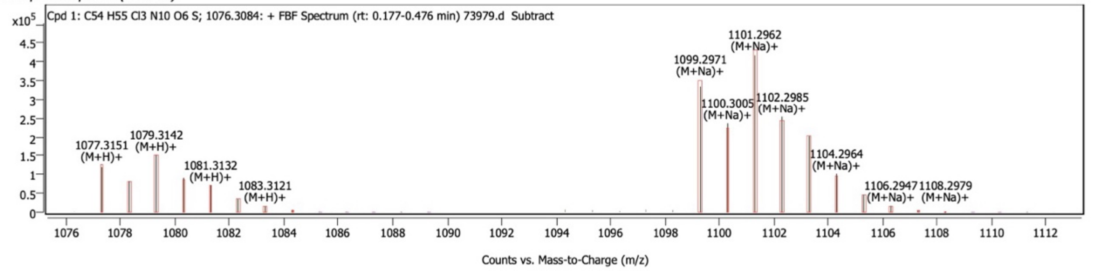

Compound ID Table

Name	Formula	Species	RT	RT Diff	Mass	CAS	ID Source	Score	Score (Lib)	Score (Tgt)
	C54 H55 Cl3 N10 O6 S	(M+H)+ (M+Na)+	0.260		1076.3084		FBF	98.24		98.24

MassHunter Qual 10.0
(End of Report)

^{1}H NMR Compound **2**:

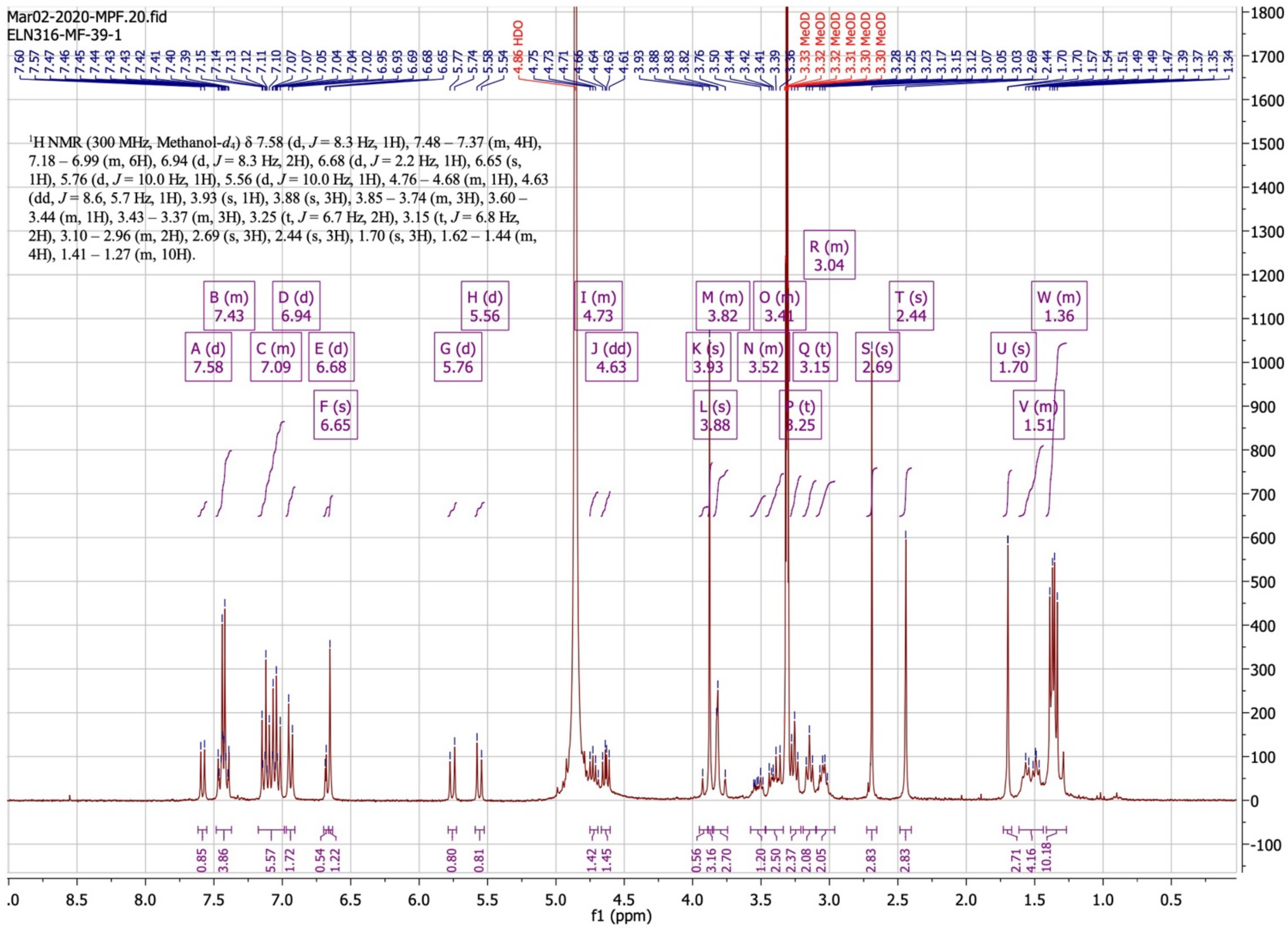

^{13}C NMR Compound **2**:

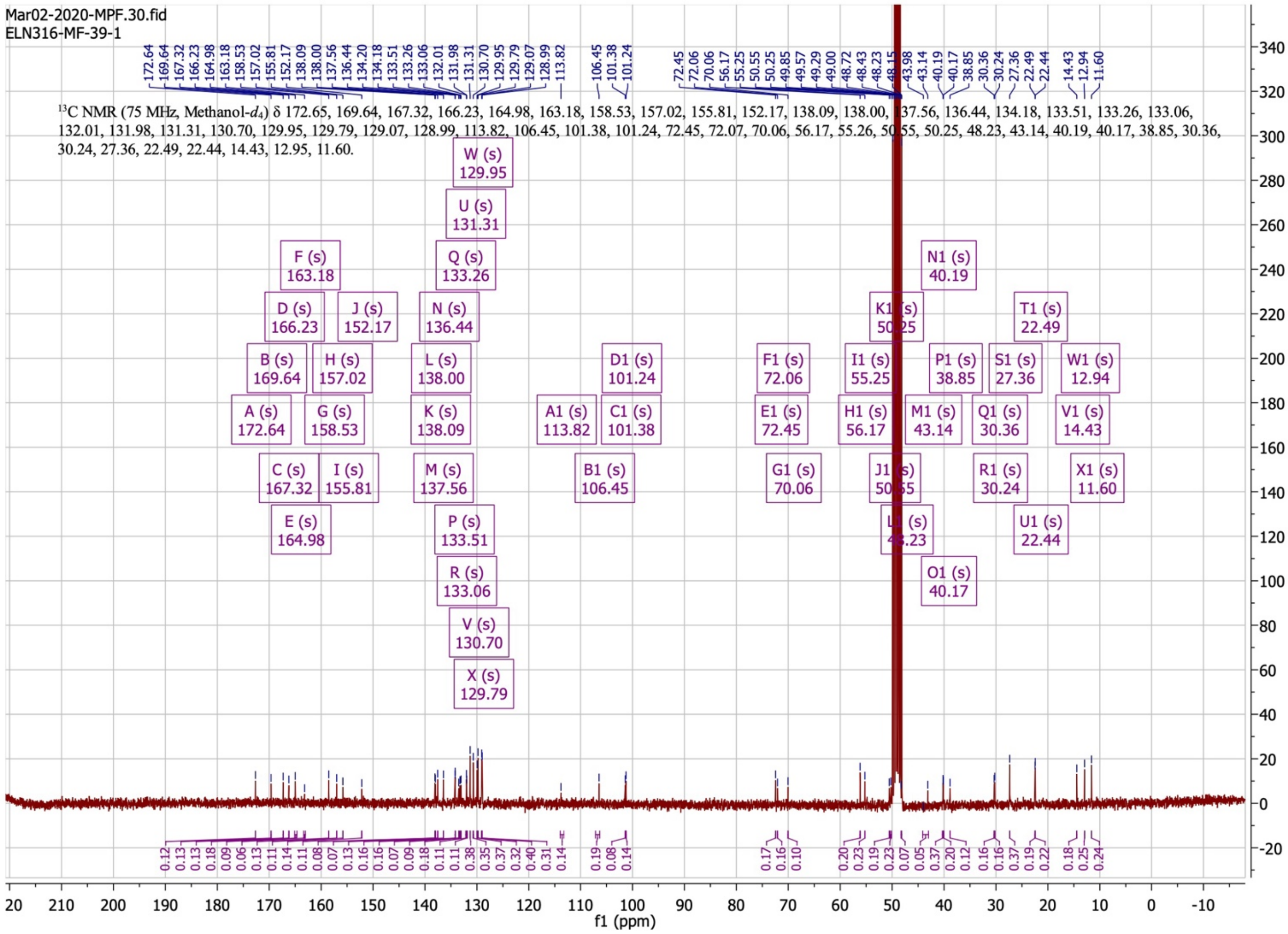

LC–MS (high pH) Compound **2**:

Openlynx Report - mficu-A- Page 1

Sample: 1 Vial:1:21
SampleID and Description:ELN316-MF-39-GV-1 File:mficu-A-226-1
Date:04-Mar-2020 Time:12:28:22 Method:C:\MassLynx\High_pH_2min.olp

Printed: Wed Mar 04 12:34:56 2020

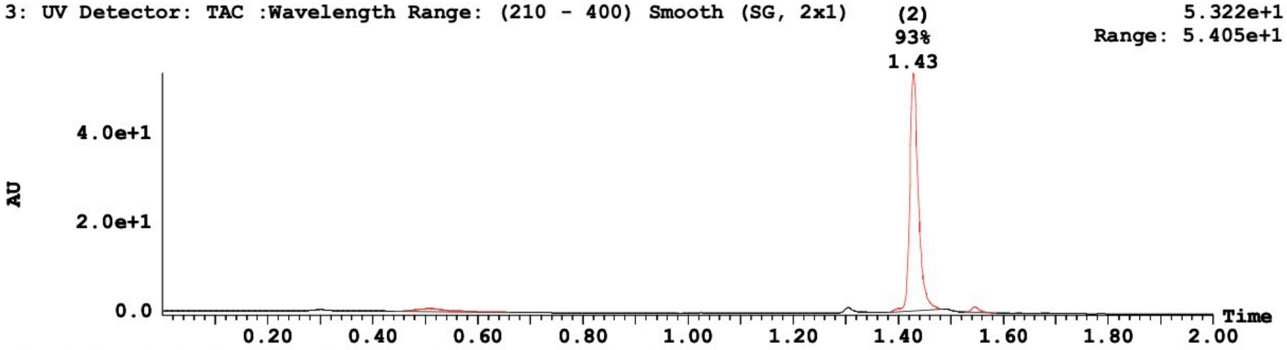

Peak Number	Compound	Time	Area %Total	Mass Found
1		0.51	5.31	Not Found
2		1.43	92.71	Not Found
3		1.55	1.98	Not Found

Peak ID	Compound	Time	Mass Found
1		0.51	Not Found

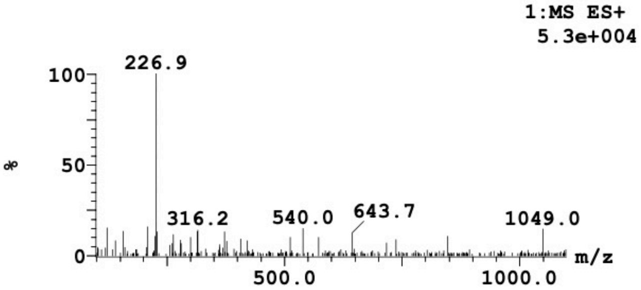

Peak ID	Compound	Time	Mass Found
1		0.51	Not Found

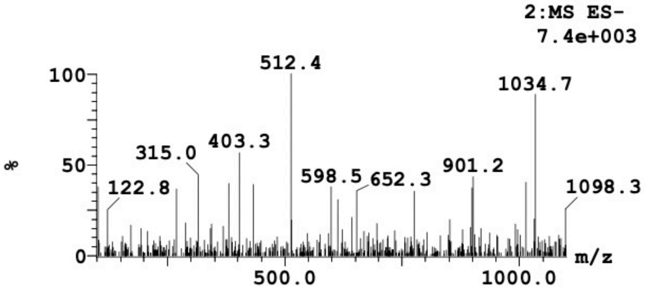

Peak ID	Compound	Time	Mass Found
2		1.43	Not Found

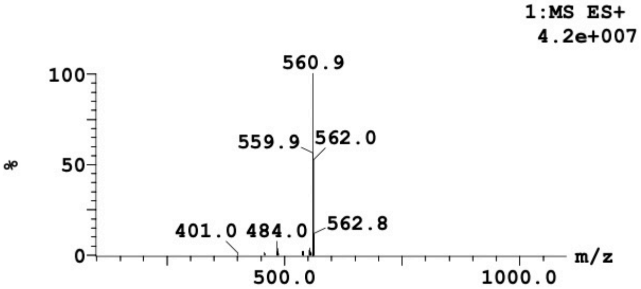

Peak ID	Compound	Time	Mass Found
2		1.43	Not Found

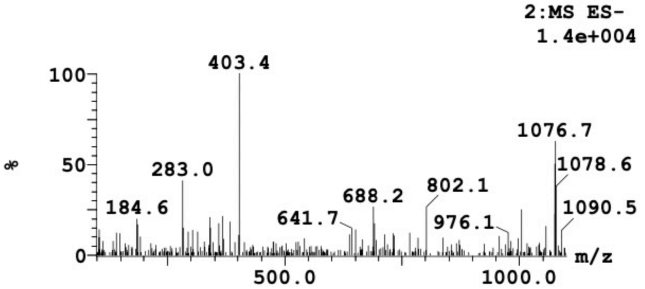

LC–MS (low pH) Compound **2**:

Openlynx Report - mficu-A- Page 1

Sample: 1 Vial:1:21

SampleID and Description:ELN316-MF-39-GV-2 File:mficu-A-227-1

Date:04-Mar-2020 Time:12:34:57 Method:C:\MassLynx\Low_pH_2min.olp

Printed: Wed Mar 04 12:41:01 2020

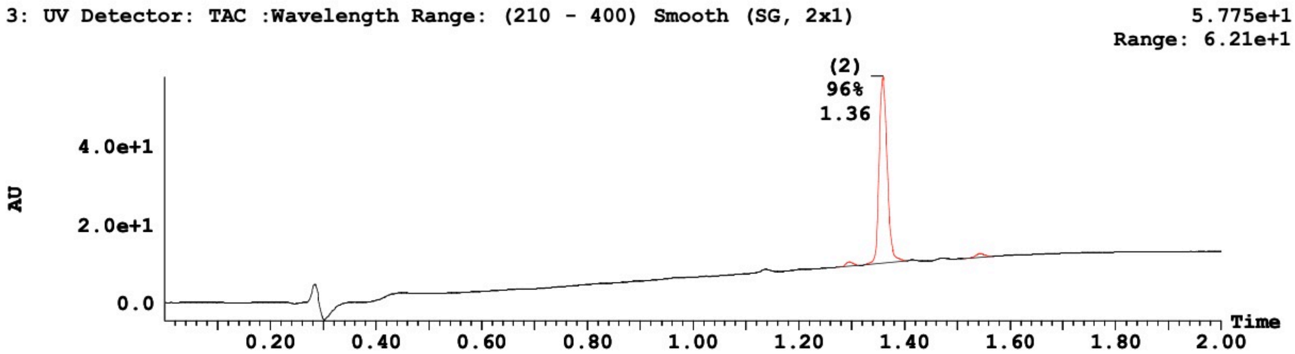

Peak Number	Compound	Time	Area %Total	Mass Found
1		1.30	1.98	Not Found
2		1.36	95.85	Not Found
3		1.54	2.17	Not Found

Peak ID	Compound	Time	Mass Found
1		1.30	Not Found

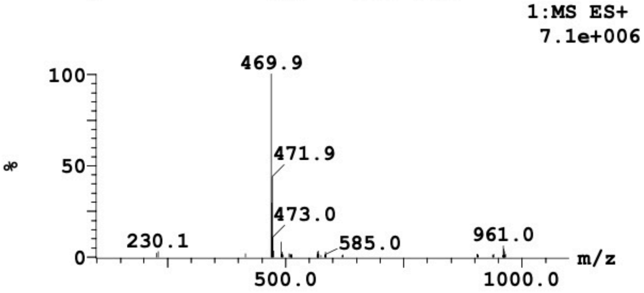

Peak ID	Compound	Time	Mass Found
1		1.30	Not Found

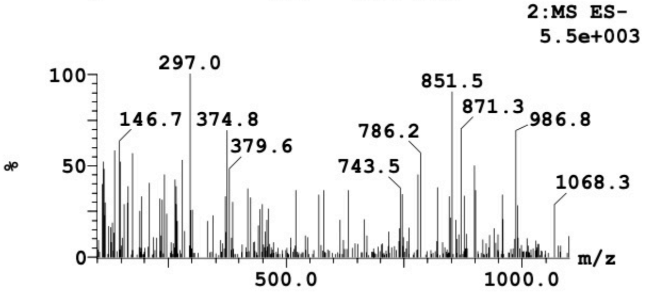

Peak ID	Compound	Time	Mass Found
2		1.36	Not Found

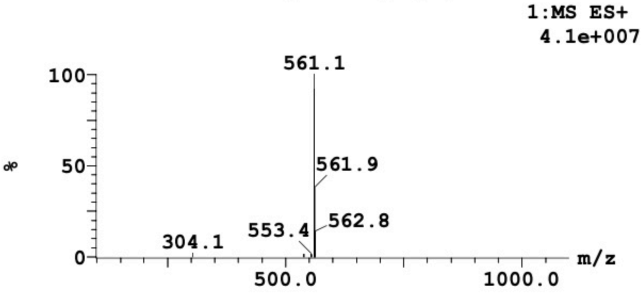

Peak ID	Compound	Time	Mass Found
2		1.36	Not Found

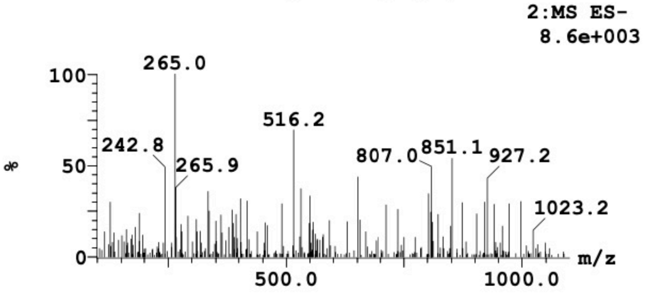

HRMS Compound **2**:

Spectrum Plot Report

Agilent | Trusted Answers

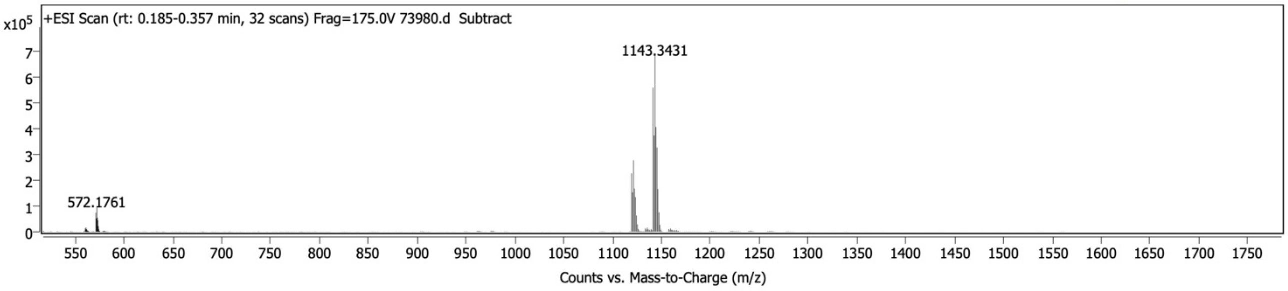

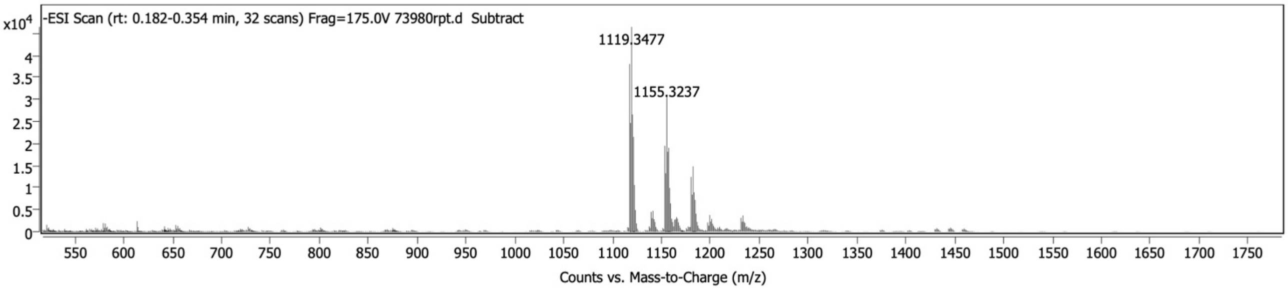

Spectrum Plot Report

Agilent | Trusted Answers

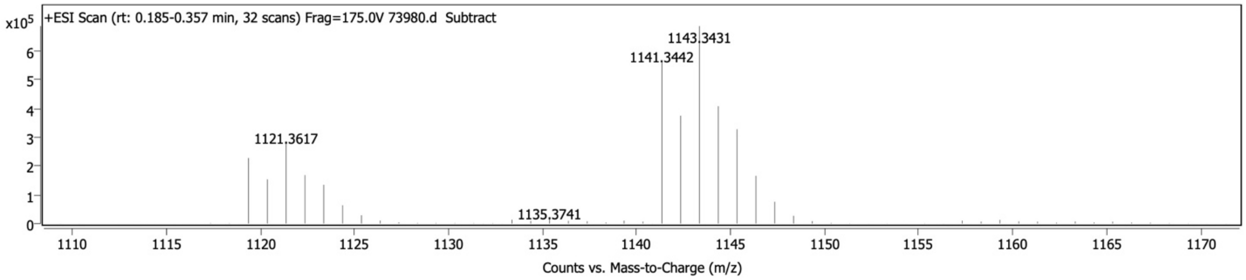

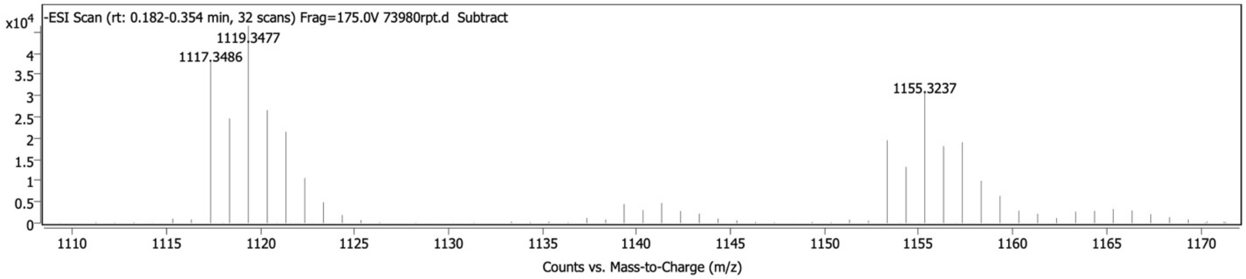

Target Screening Report

Agilent | Trusted Answers

Compound Spectra (overlaid)

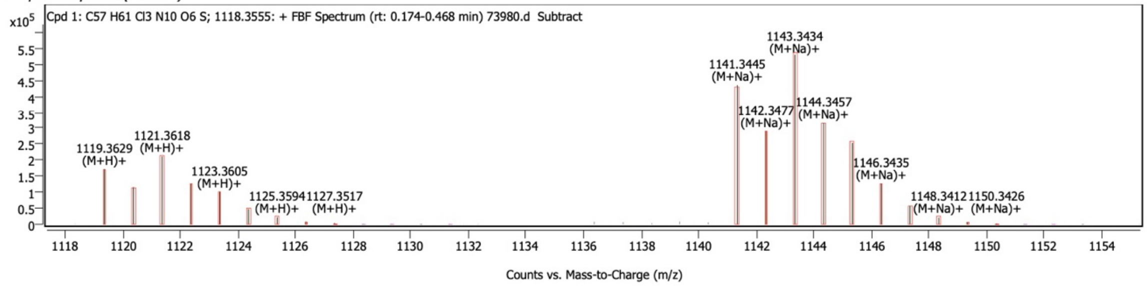

Compound ID Table

Name	Formula	Species	RT	RT Diff	Mass	CAS	ID Source	Score	Score (Lib)	Score (Tgt)
	C57 H61 Cl3 N10 O6 S	(M+H)+ (M+Na)+	0.241		1118.3555		FBF	99.63		99.63

MassHunter Qual 10.0
(End of Report)

^{1}H NMR Compound **4**:

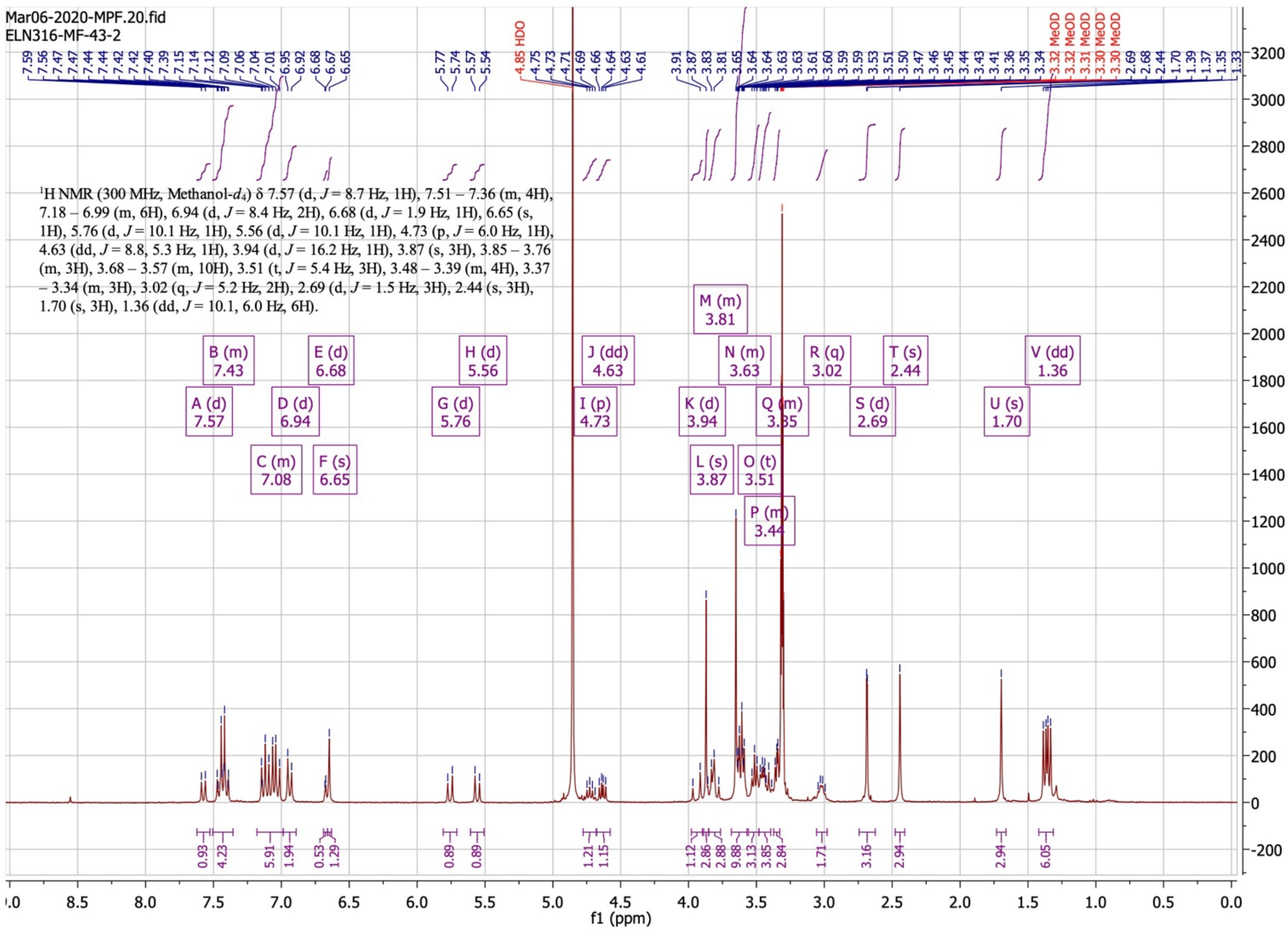

^{13}C NMR Compound **4**:

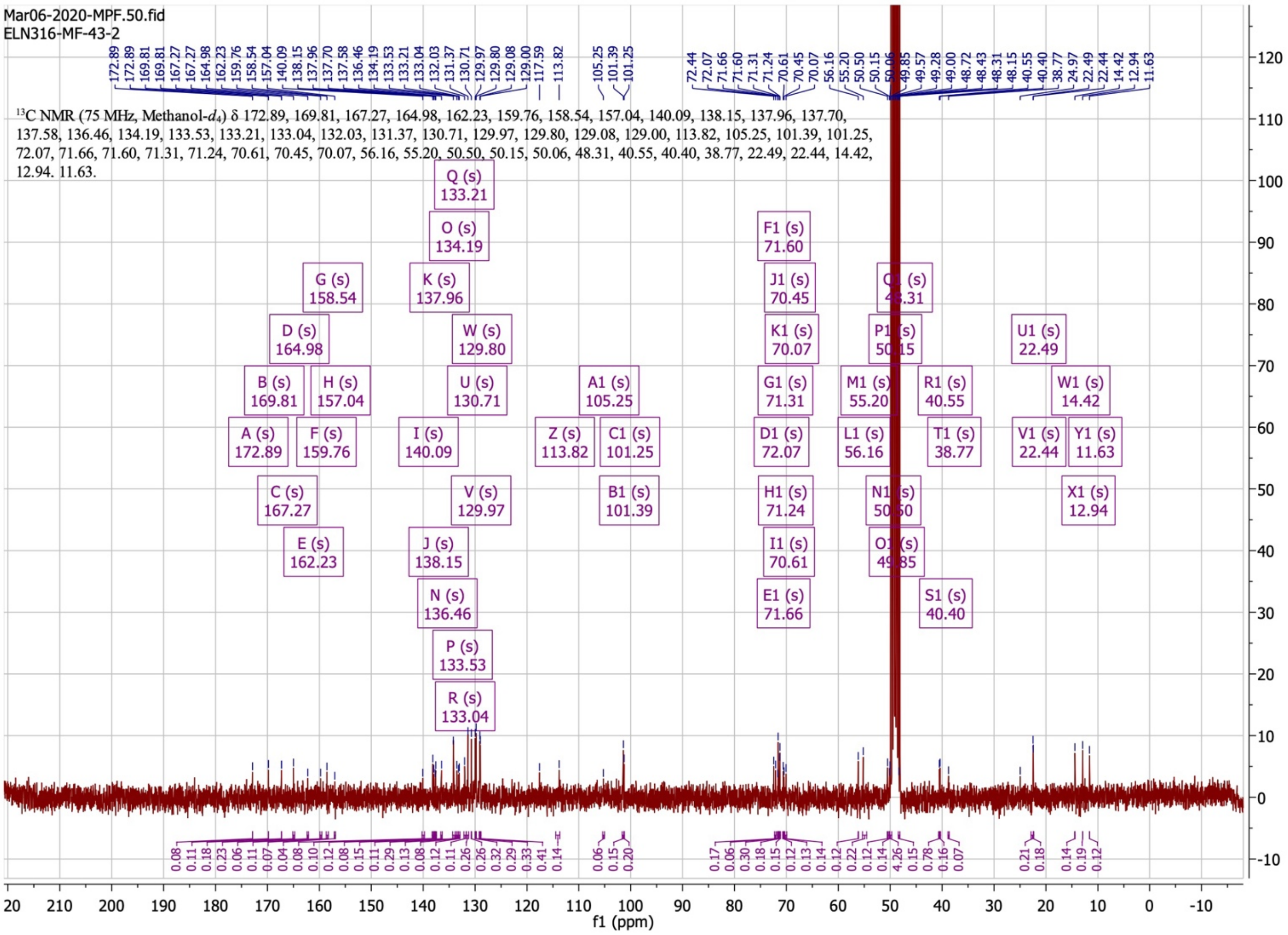

LC–MS (high pH) Compound **4**:

Openlynx Report - mficu-I- Page 1

Sample: 1	Vial:1:42	SampleID and Description:ELN316-MF-43-2
File:mficu-I-131-1	Date:10-Mar-2020	Time:15:59:24
Method:C:\MassLynx\High_pH2min.olp		

Printed: Tue Mar 10 16:02:08 2020

3: UV Detector: TAC: Wavelength Range: (210 - 400) Smooth (SG, 2x1) 4.374e+1
Range: 4.476e+1

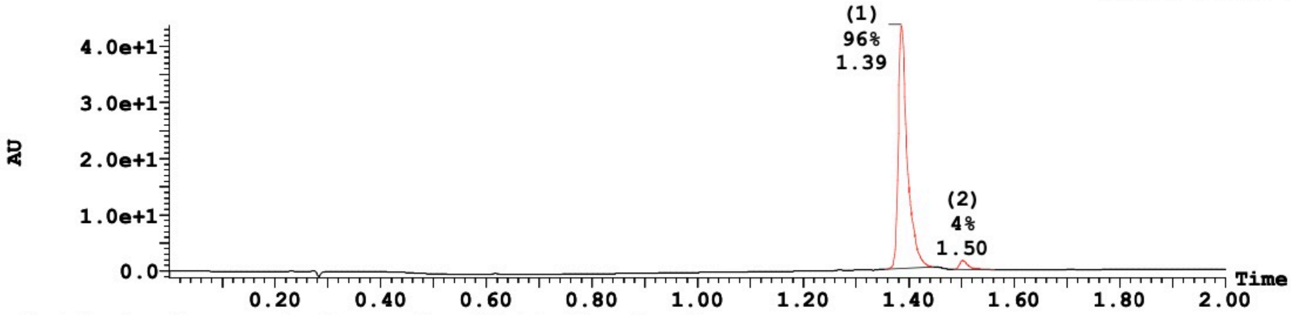

Peak Number	Compound	Time	Area %Total	Mass Found
1		1.39	96.43	Not Found
2		1.50	3.57	Not Found

Peak ID	Compound	Time	Mass Found
1		1.39	Not Found

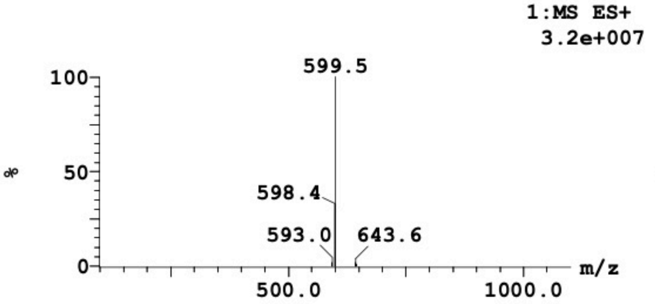

Peak ID	Compound	Time	Mass Found
1		1.39	Not Found

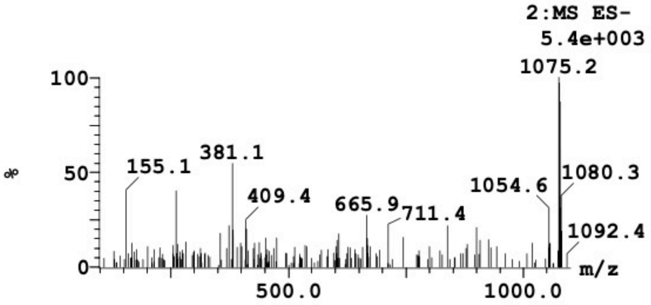

Peak ID	Compound	Time	Mass Found
2		1.50	Not Found

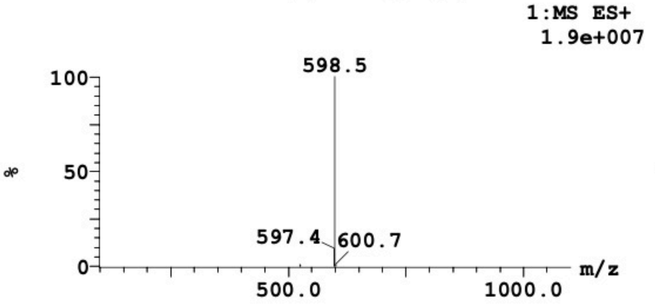

Peak ID	Compound	Time	Mass Found
2		1.50	Not Found

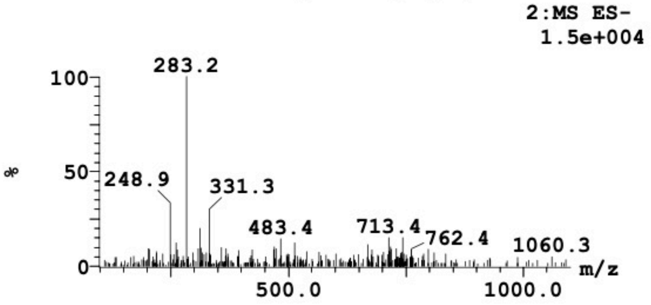

LC–MS (low pH) Compound **4**:

Openlynx Report - mficu-A- Page 1

Sample: 1	Vial:1:42	SampleID and Description:ELN316-MF-43-2
File:mficu-A-232-1	Date:10-Mar-2020	Time:17:33:26
Method:C:\MassLynx\Low_pH_2min.olp		

Printed: Tue Mar 10 17:36:30 2020

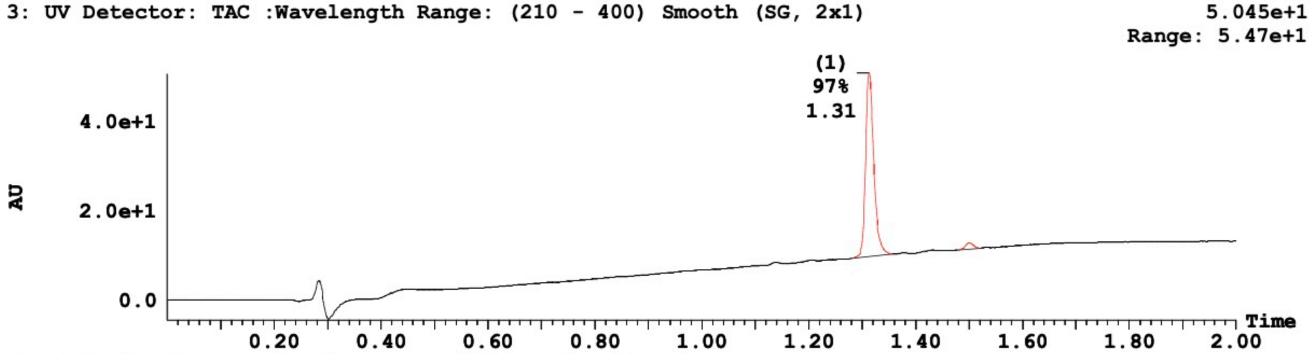

Peak Number	Compound	Time	Area %Total	Mass Found
1		1.31	96.93	Not Found
2		1.50	3.07	Not Found

Peak ID	Compound	Time	Mass Found
1		1.31	Not Found

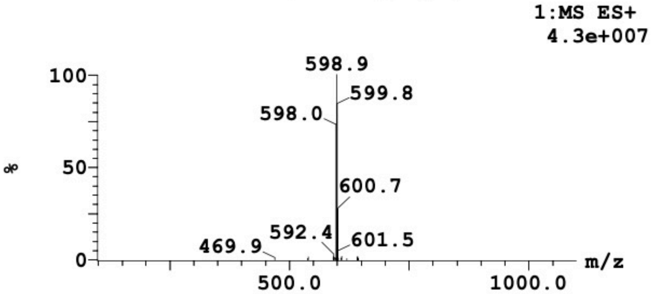

Peak ID	Compound	Time	Mass Found
1		1.31	Not Found

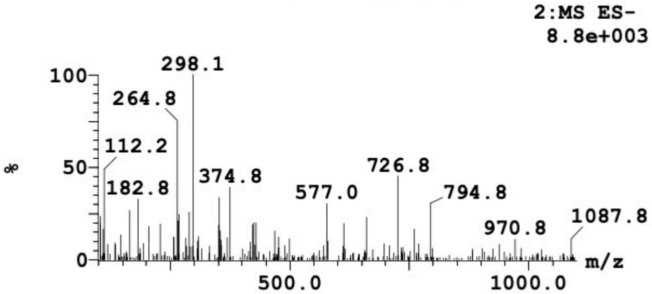

Peak ID	Compound	Time	Mass Found
2		1.50	Not Found

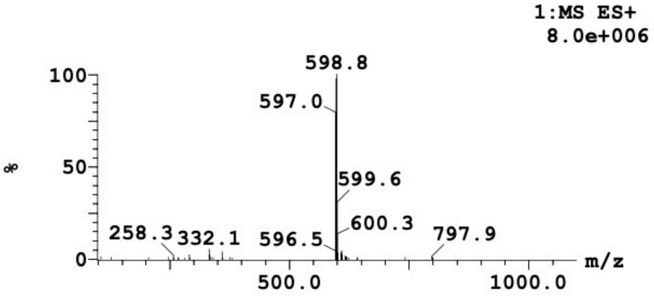

Peak ID	Compound	Time	Mass Found
2		1.50	Not Found

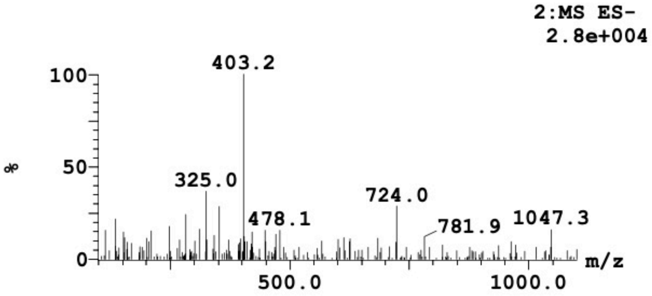

HRMS Compound **4**:

Spectrum Plot Report

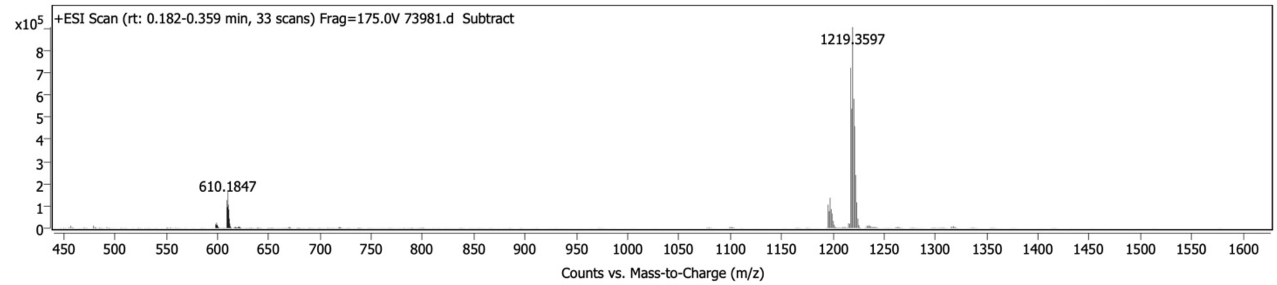

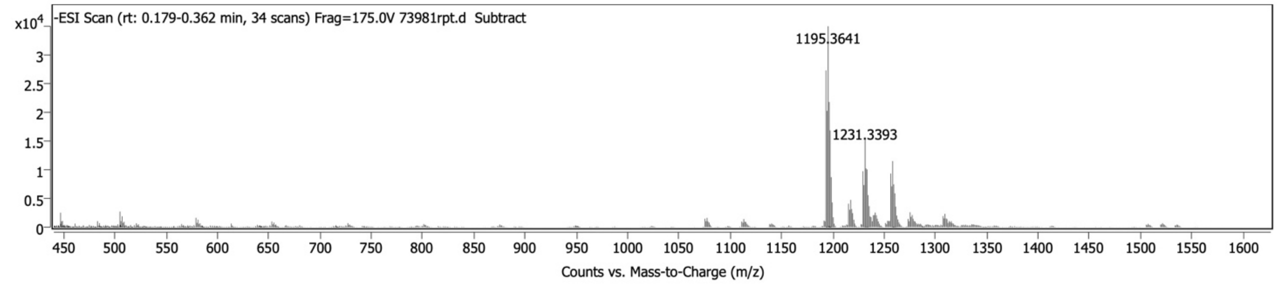

Spectrum Plot Report

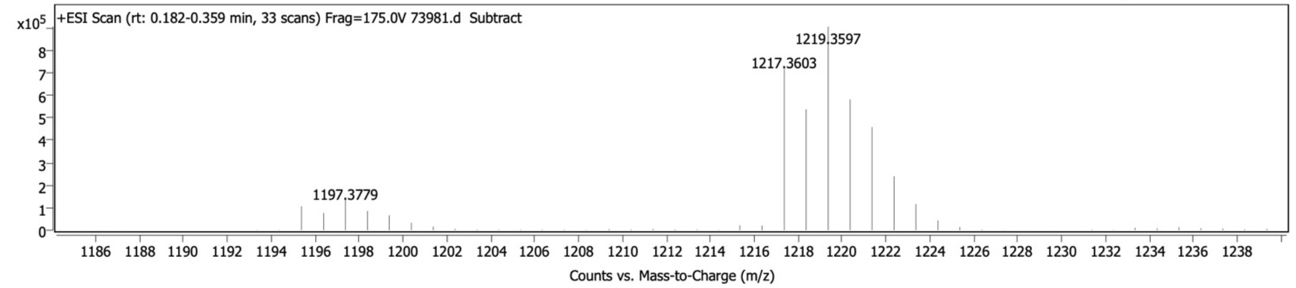

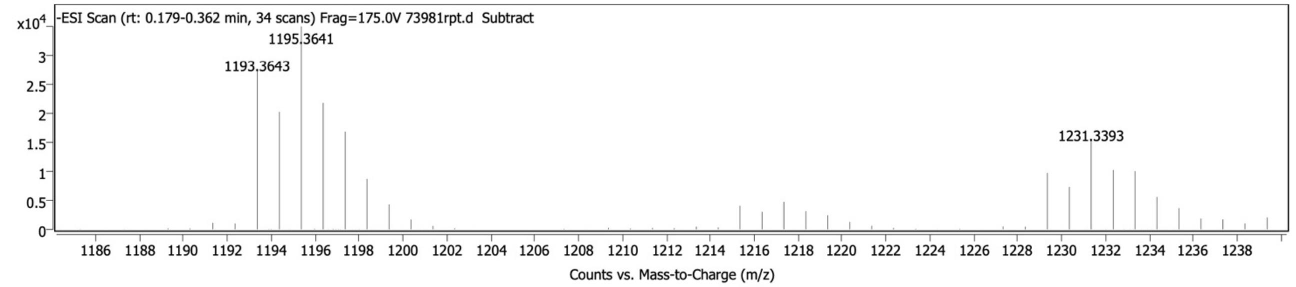

Target Screening Report

Compound Spectra (overlaid)

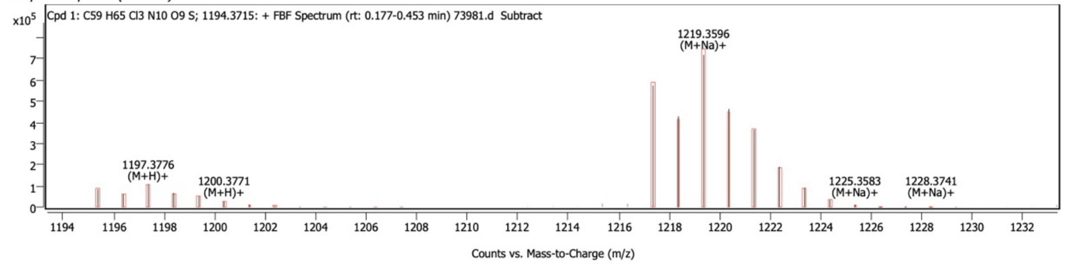

Compound ID Table

Name	Formula	Species	RT	RT Diff	Mass	CAS	ID Source	Score	Score (Lib)	Score (Tgt)
	C59 H65 Cl3 N10 O9 S	(M+H)+<br>(M+Na)+	0.249		1194.3715		FBF	99.20		99.20

MassHunter Qual 10.0
(End of Report)

^{1}H NMR Compound **5**:

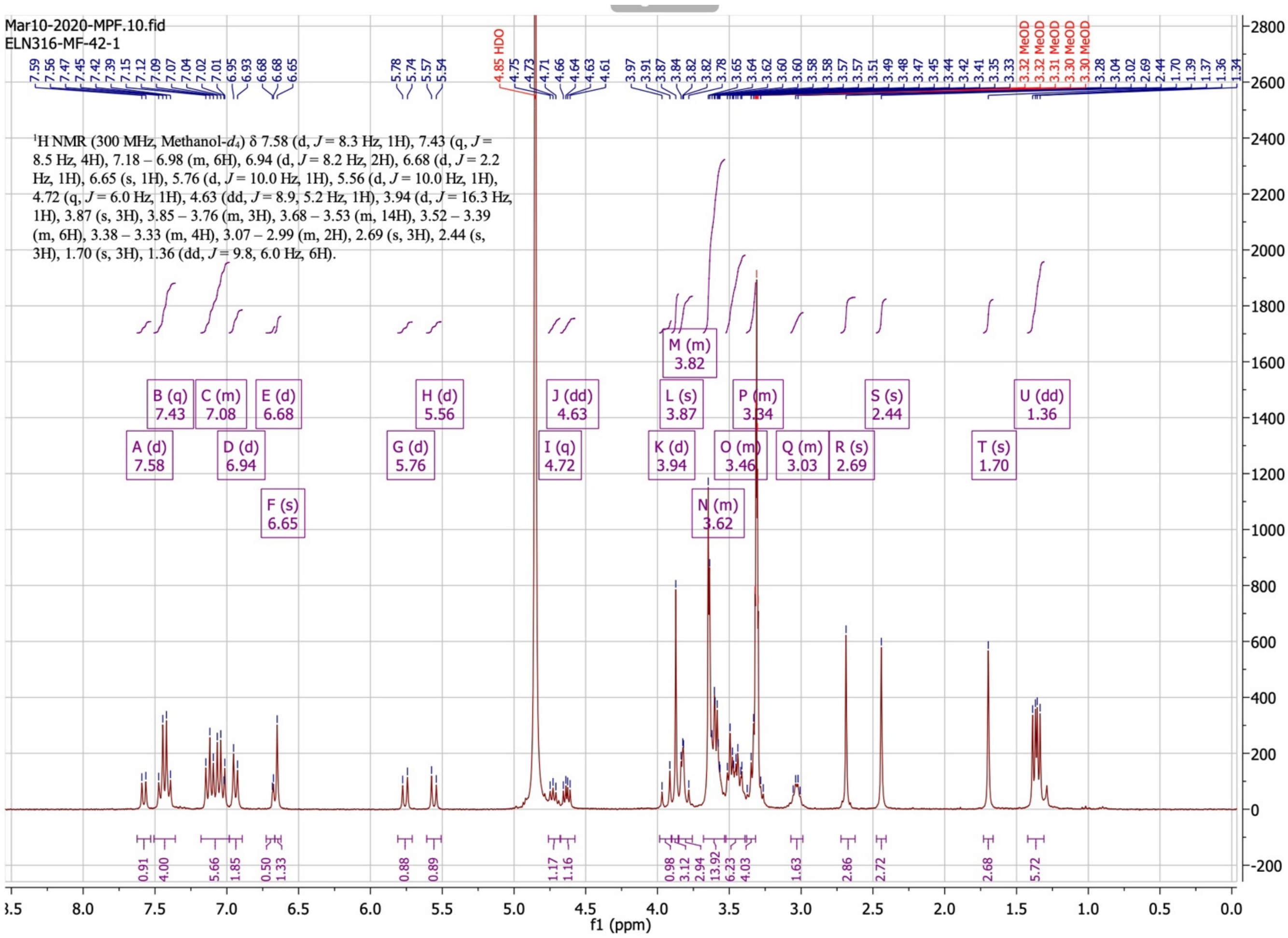

^{13}C NMR Compound **5**:

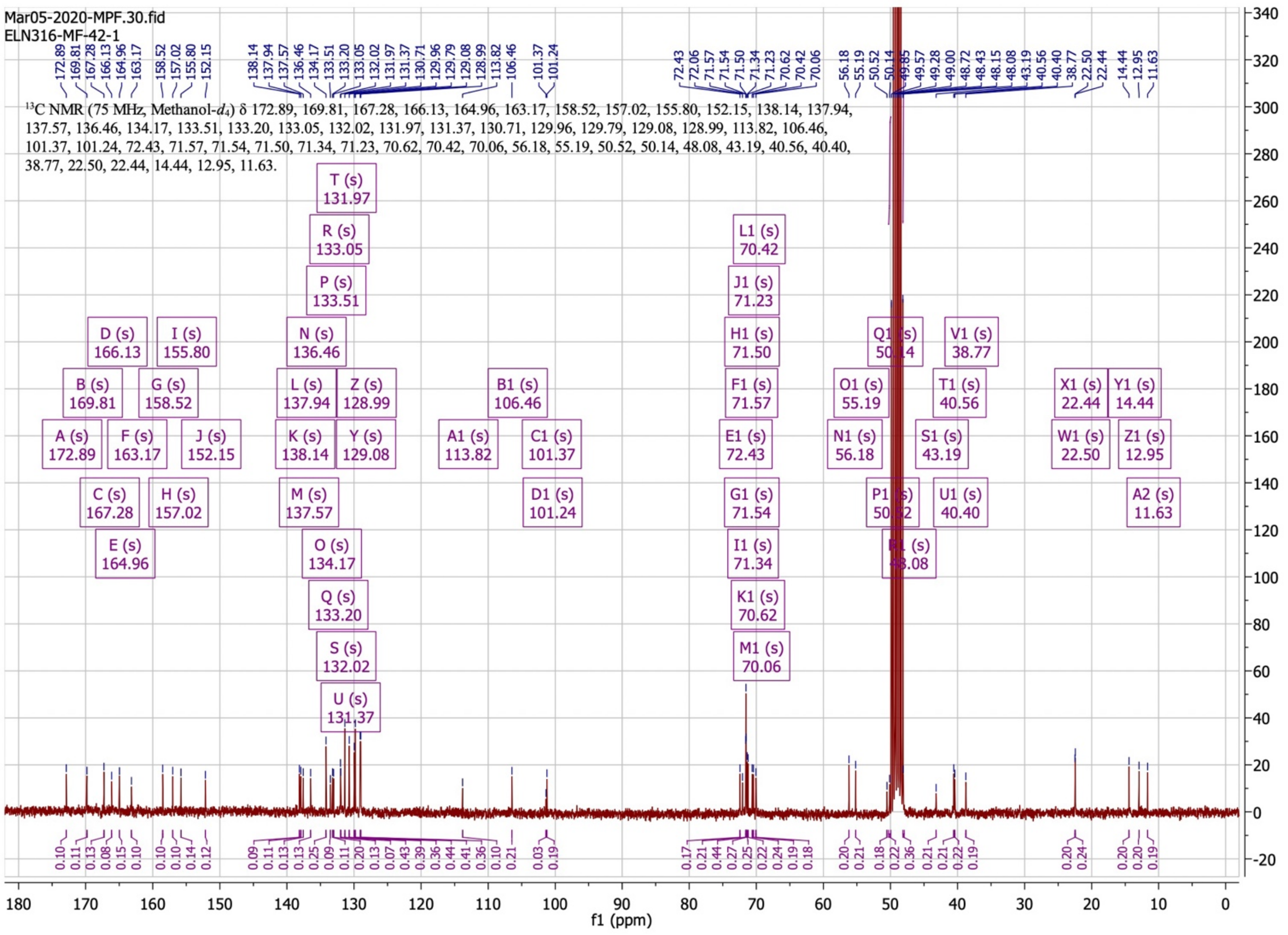

LC–MS (high pH) Compound **5**:

Openlynx Report - mficu-I- Page 1

Sample: 1 Vial:1:40 SampleID and Description:ELN316-MF-42-1

File:mficu-I-129-1 Date:10-Mar-2020 Time:15:52:38

Method:C:\MassLynx\High_pH2min.olp

Printed: Tue Mar 10 15:56:30 2020

3: UV Detector: TAC: Wavelength Range: (210 - 400) Smooth (SG, 2x1) 2.182e+2 Range: 2.19e+2

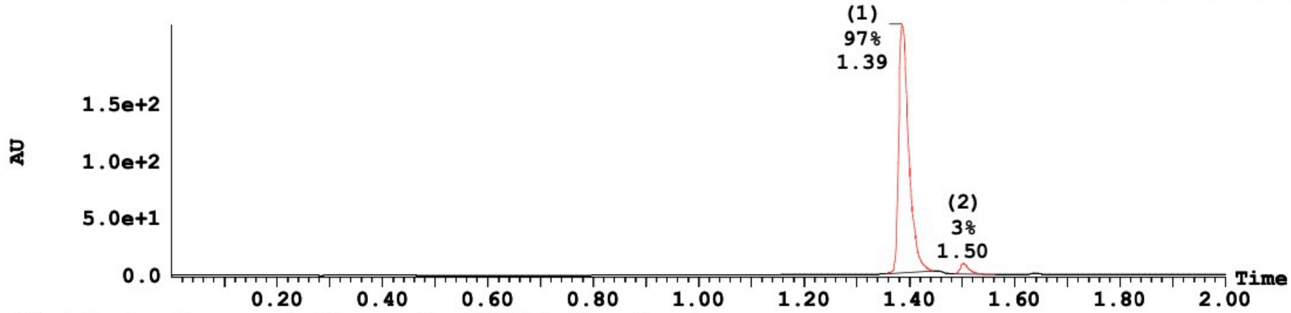

Peak Number	Compound	Time	Area %Total	Mass Found
1		1.39	96.54	Not Found
2		1.50	3.46	Not Found

Peak ID	Compound	Time	Mass Found
1		1.39	Not Found

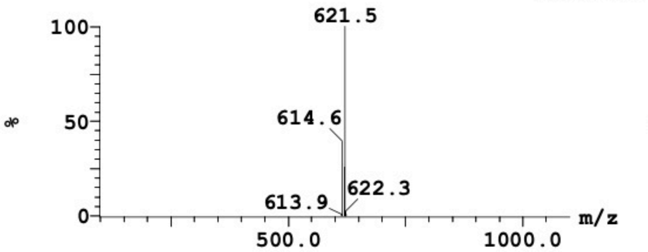

Peak ID	Compound	Time	Mass Found
1		1.39	Not Found

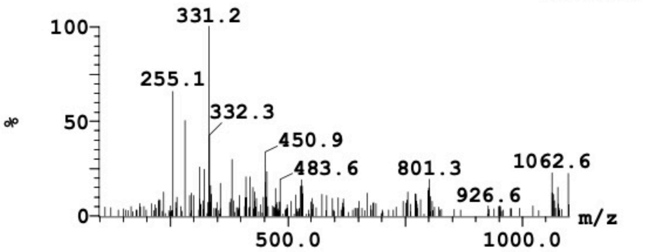

Peak ID	Compound	Time	Mass Found
2		1.50	Not Found

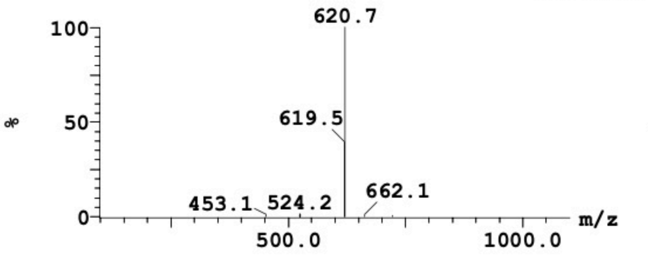

Peak ID	Compound	Time	Mass Found
2		1.50	Not Found

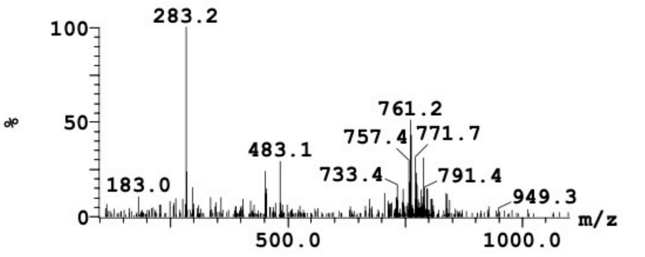

LC–MS (low pH) Compound **5**:

Openlynx Report - mficu-A- Page 1

Sample: 1	Vial:1:40	SampleID and Description:ELN316-MF-42-1
File:mficu-A-230-1	Date:10-Mar-2020	Time:17:27:08
Method:C:\MassLynx\Low_pH_2min.olp		

Printed: Tue Mar 10 17:30:19 2020

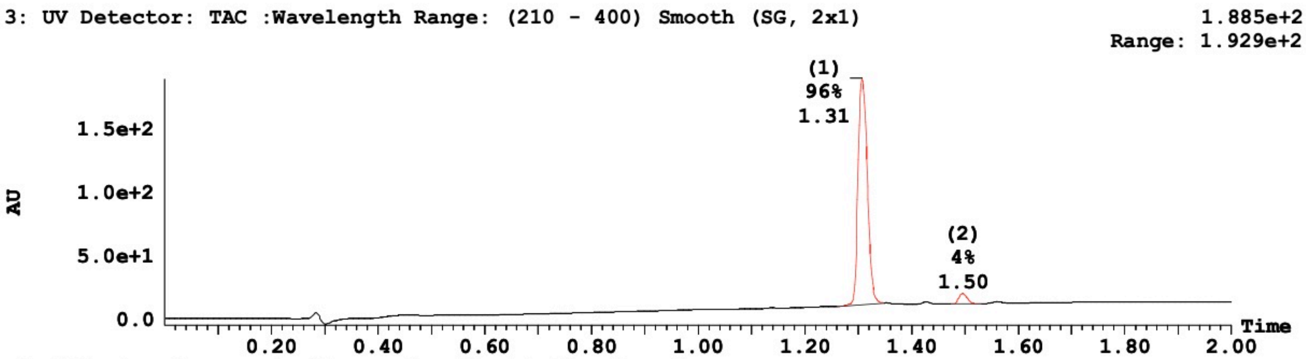

Peak Number	Compound	Time	Area %Total	Mass Found
1		1.31	95.98	Not Found
2		1.50	4.02	Not Found

Peak ID	Compound	Time	Mass Found
1		1.31	Not Found

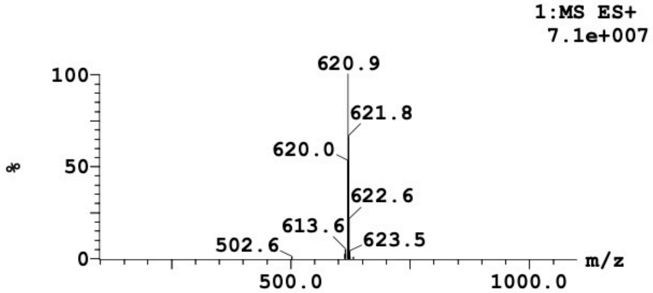

Peak ID	Compound	Time	Mass Found
1		1.31	Not Found

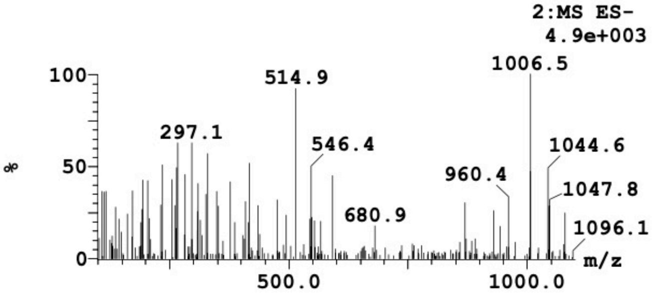

Peak ID	Compound	Time	Mass Found
2		1.50	Not Found

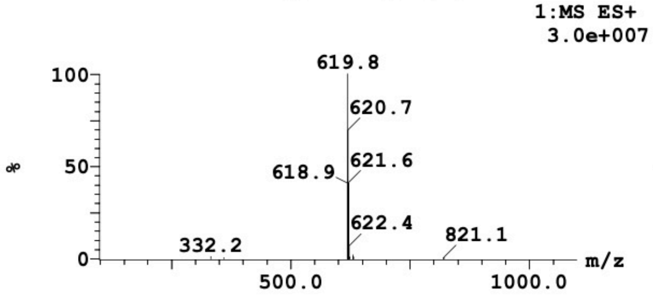

Peak ID	Compound	Time	Mass Found
2		1.50	Not Found

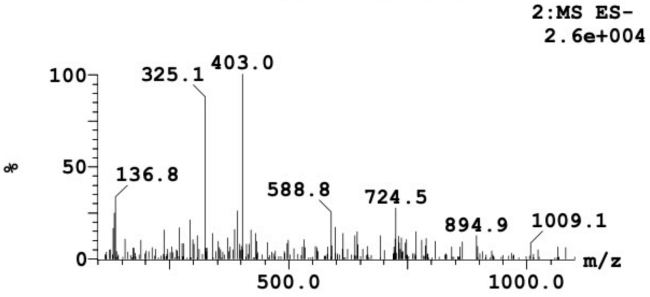

HRMS Compound **5**:

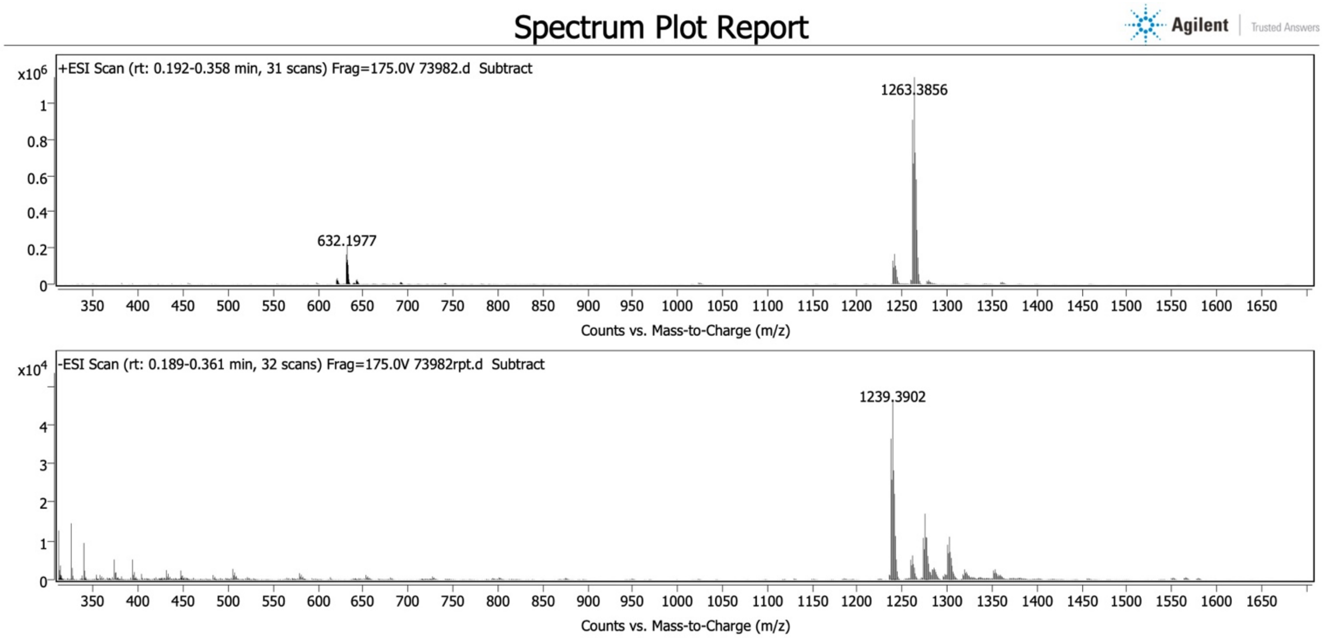

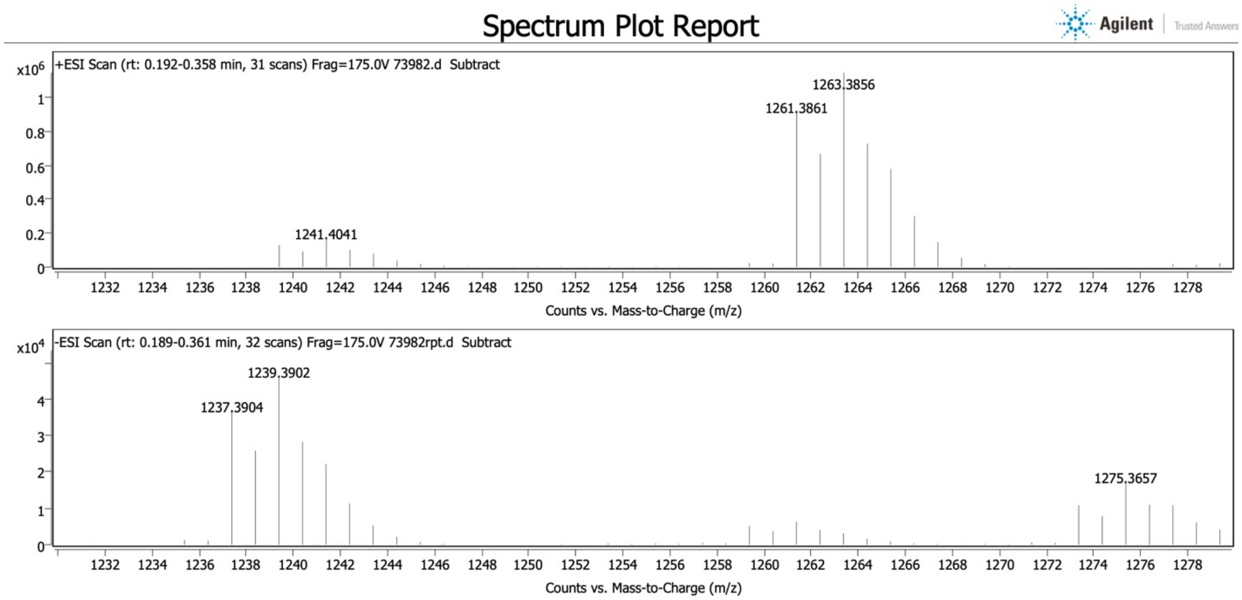

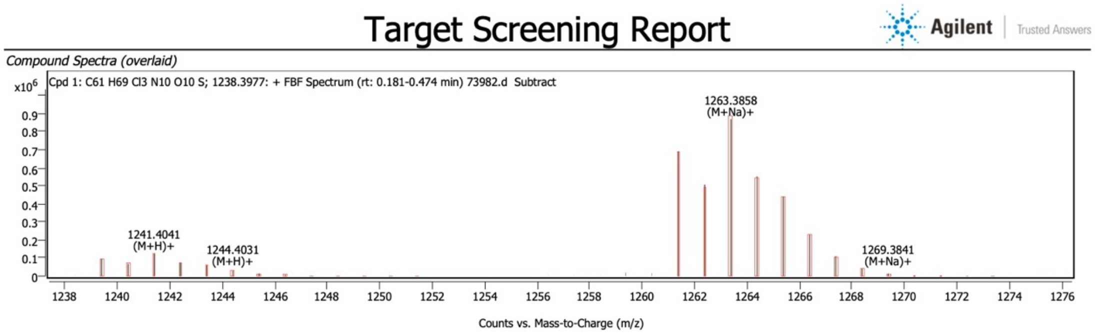

Compound ID Table

Name	Formula	Species	RT	RT Diff	Mass	CAS	ID Source	Score	Score (Lib)	Score (Tgt)
	C61 H69 Cl3 N10 O10 S	(M+H)+<br>(M+Na)+	0.253		1238.3977		FBF	99.56		99.56

MassHunter Qual 10.0
(End of Report)

^{1}H NMR Compound **6**:

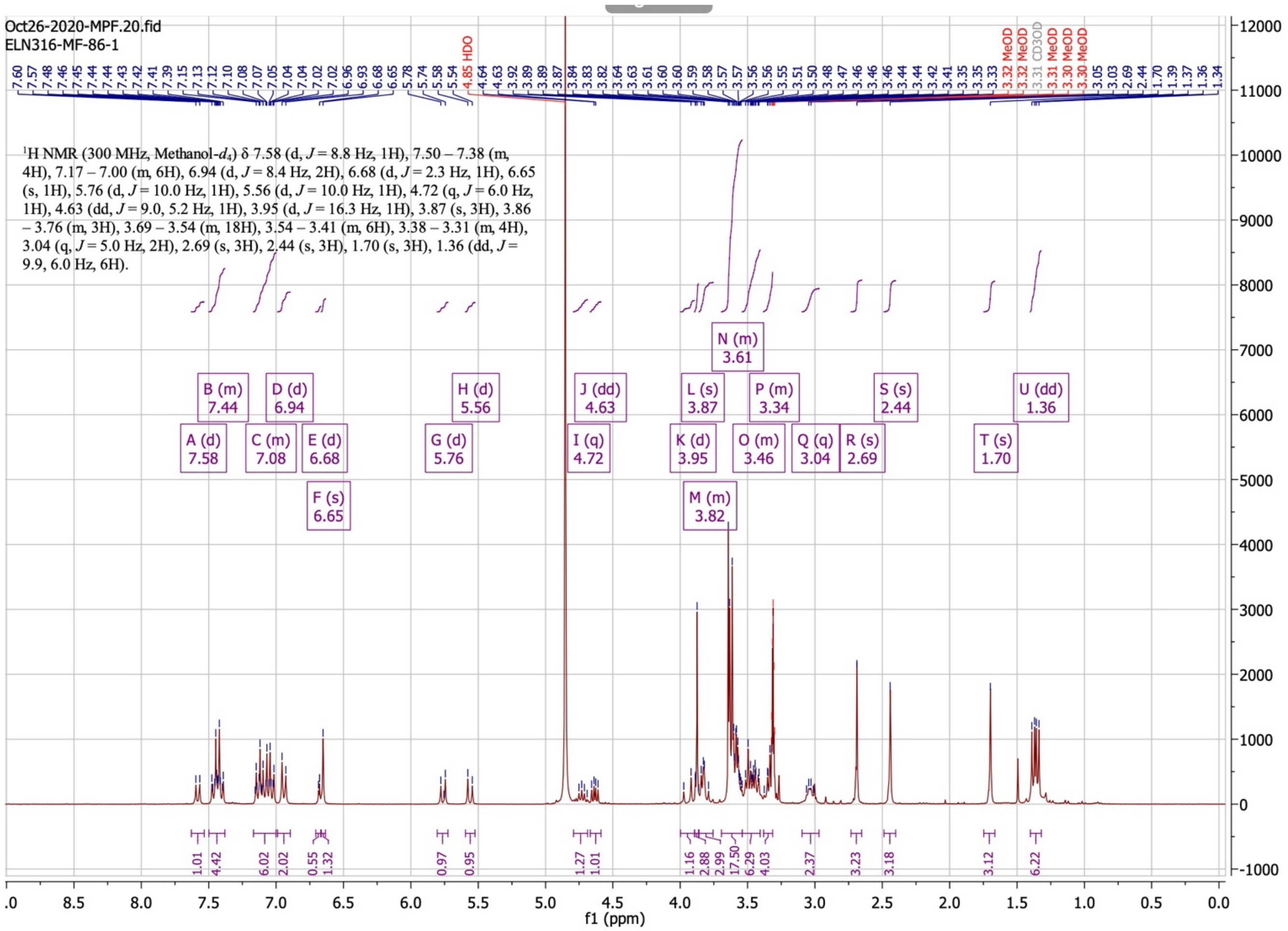

^{13}C NMR Compound **6**:

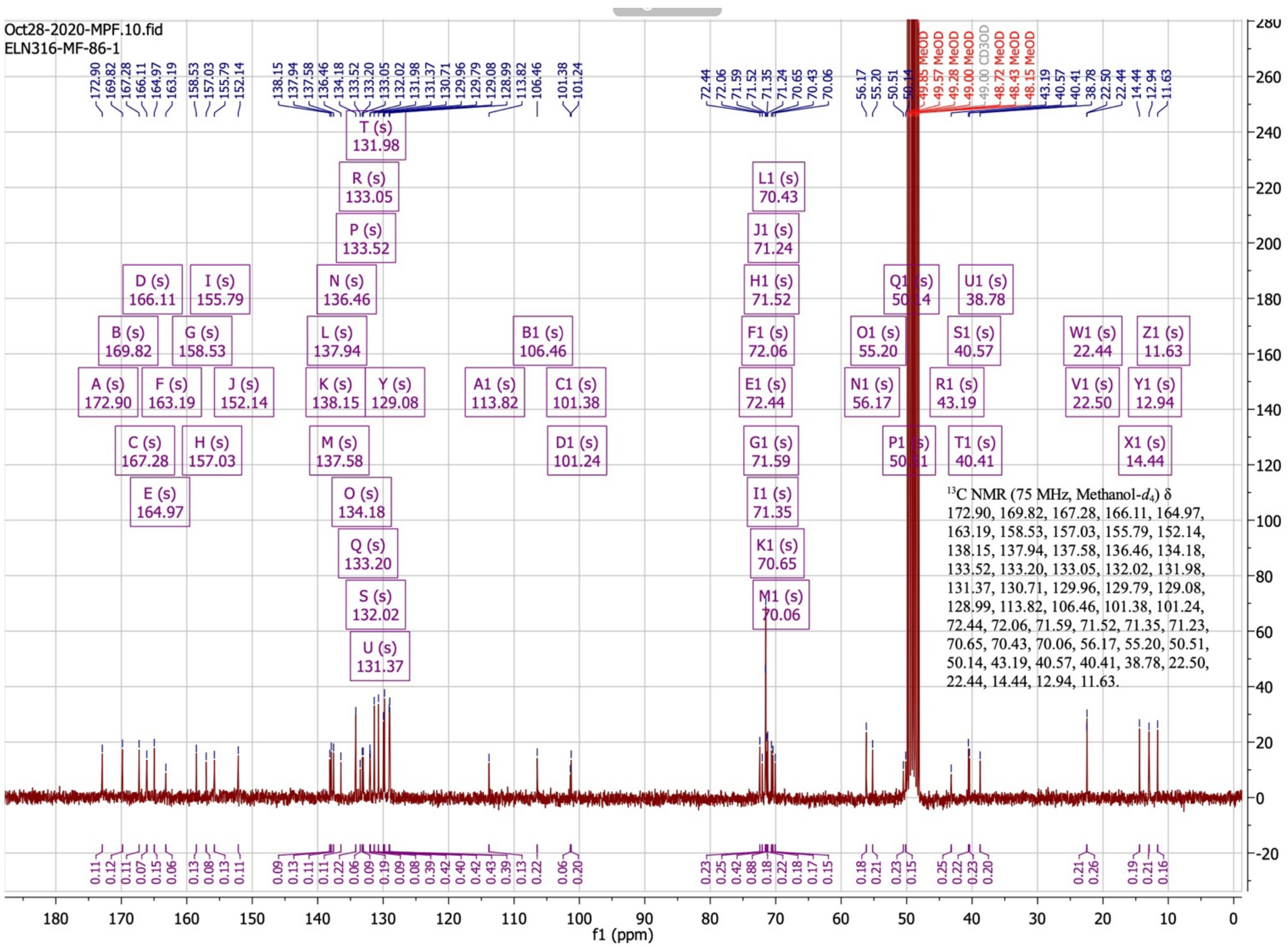

LC–MS (high pH) Compound **6**:

Openlynx Report - mficu-A- Page 1

Sample: 1 Vial:1:1
SampleID and Description:ELN316-MF-86-1-1 File:mficu-A-449-1
Date:26-Oct-2020 Time:19:35:32
Method:C:\MassLynx\High_pH_HIGH_mass_2min.olp

Printed: Mon Oct 26 19:39:20 2020

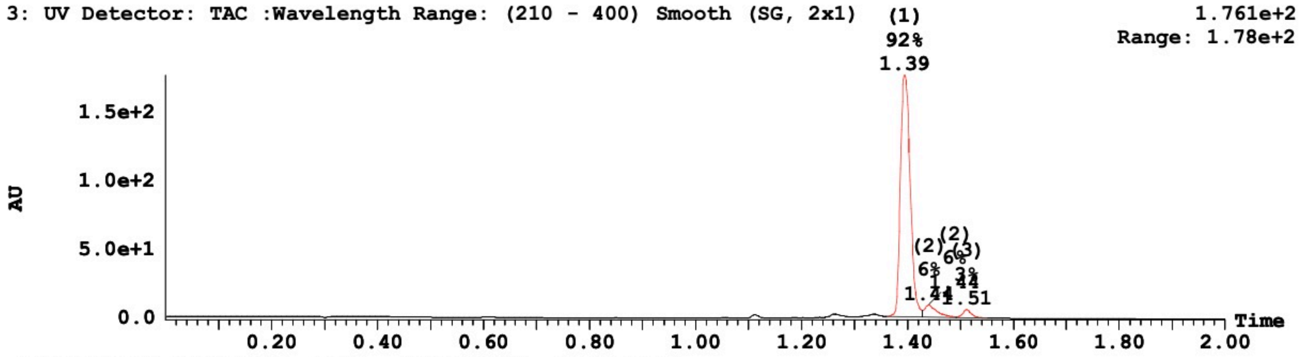

Peak Number	Compound	Time	Area %Total	Mass Found
1		1.39	91.62	Not Found
2		1.44	5.77	Not Found
3		1.51	2.61	Not Found

Peak ID	Compound	Time	Mass Found
1		1.39	Not Found

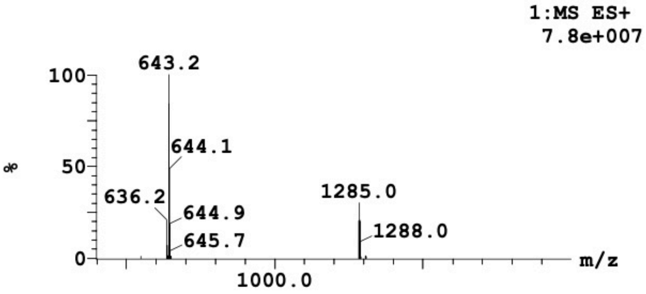

Peak ID	Compound	Time	Mass Found
1		1.39	Not Found

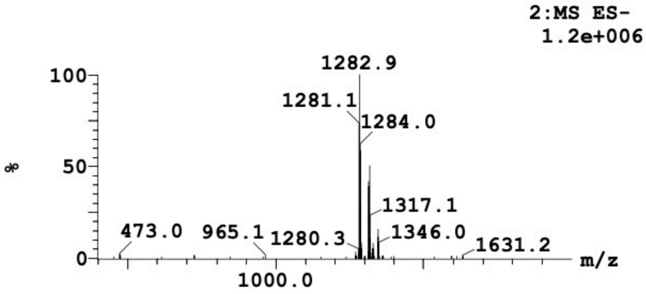

Peak ID	Compound	Time	Mass Found
2		1.44	Not Found

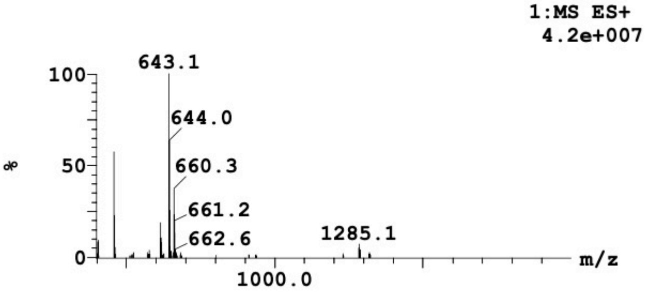

Peak ID	Compound	Time	Mass Found
2		1.44	Not Found

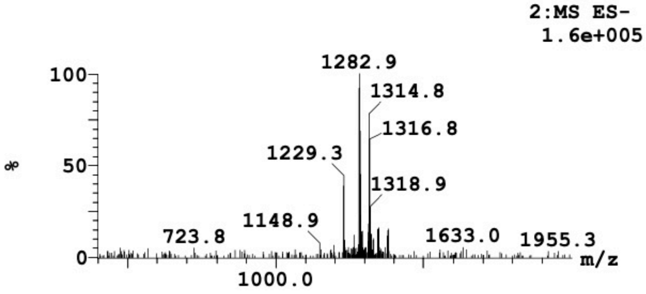

LC–MS (low pH) Compound **6**:

Openlynx Report - mficu-A- Page 1

Sample: 1 Vial:1:1

SampleID and Description:ELN316-MF-86-1-2 File:mficu-A-450-1

Date:26-Oct-2020 Time:19:39:20

Method:C:\MassLynx\Low_pH_2min_HIGH_mass.olp

Printed: Mon Oct 26 19:45:25 2020

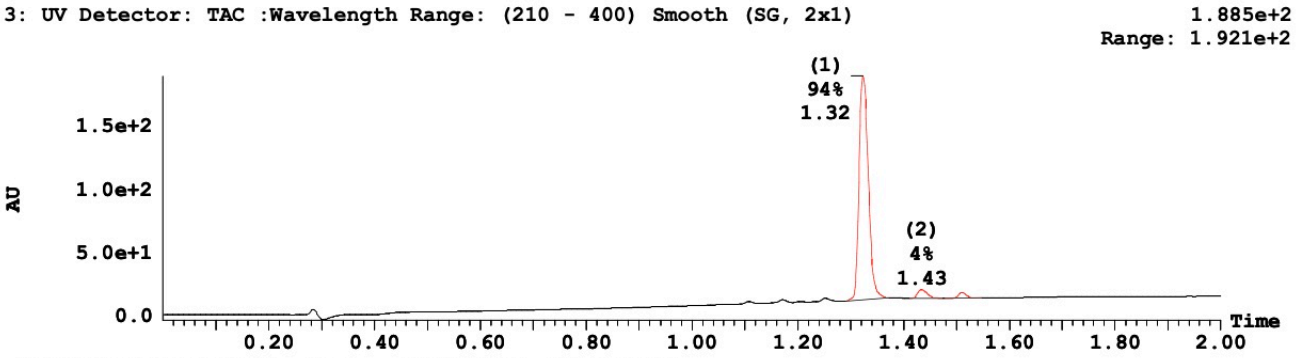

Peak Number	Compound	Time	Area %Total	Mass Found
1		1.32	93.96	Not Found
2		1.43	3.96	Not Found
3		1.51	2.08	Not Found

Peak ID	Compound	Time	Mass Found
1		1.32	Not Found

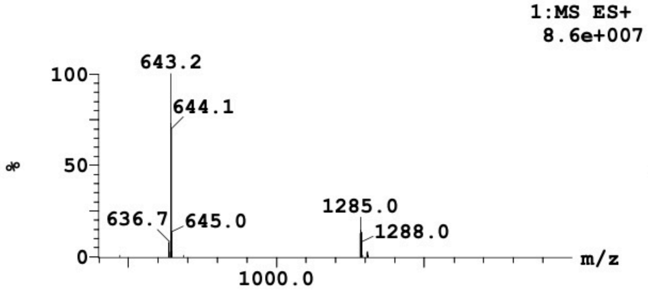

Peak ID	Compound	Time	Mass Found
1		1.32	Not Found

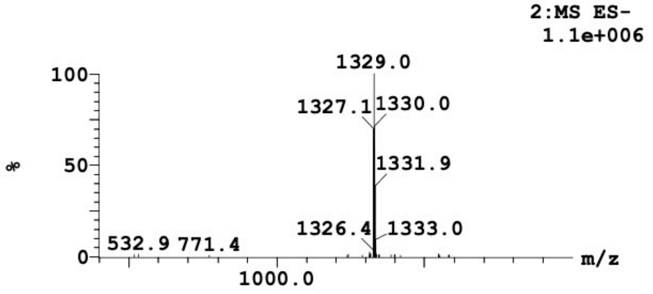

Peak ID	Compound	Time	Mass Found
2		1.43	Not Found

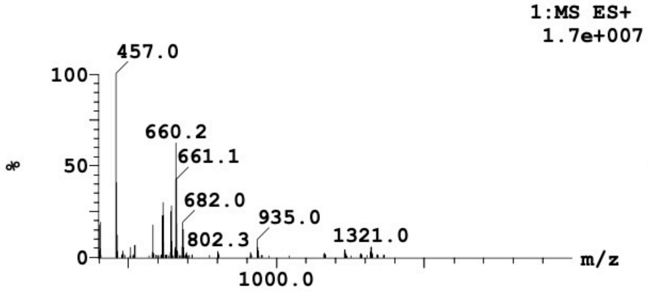

Peak ID	Compound	Time	Mass Found
2		1.43	Not Found

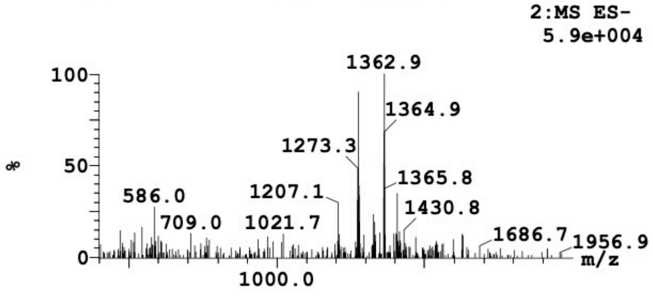

HRMS Compound **6**:

Spectrum Plot Report

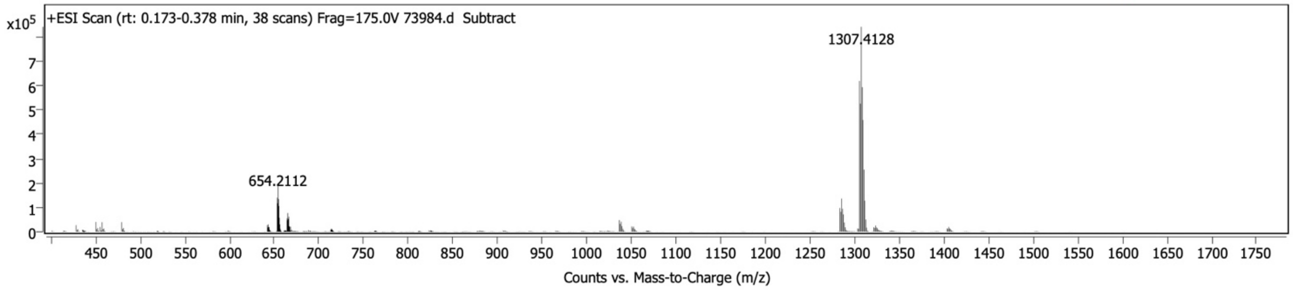

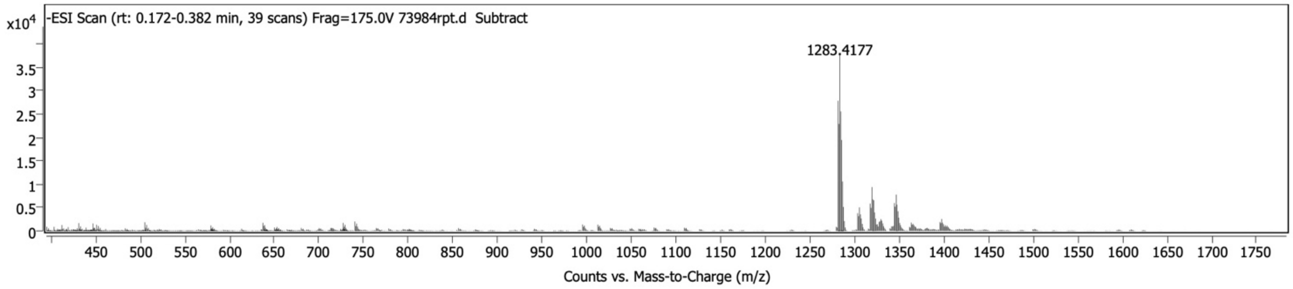

Spectrum Plot Report

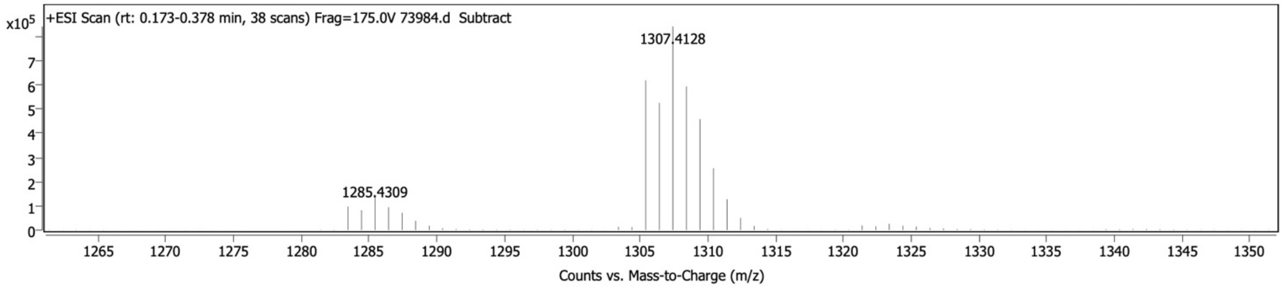

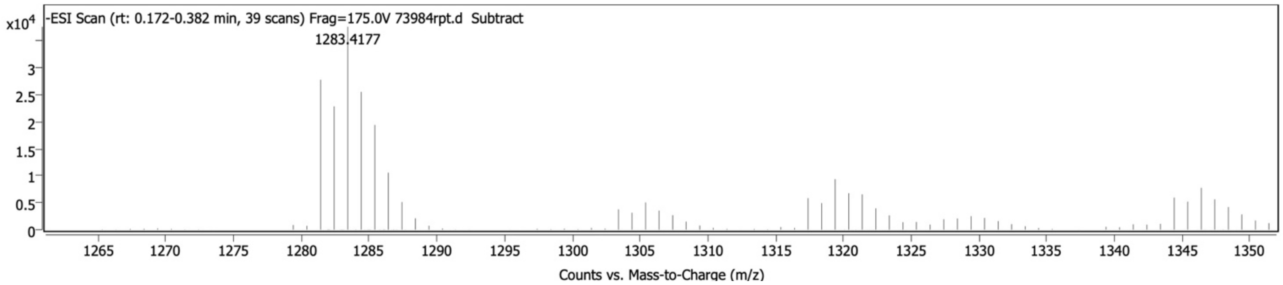

Target Screening Report

Compound Spectra (overlaid)

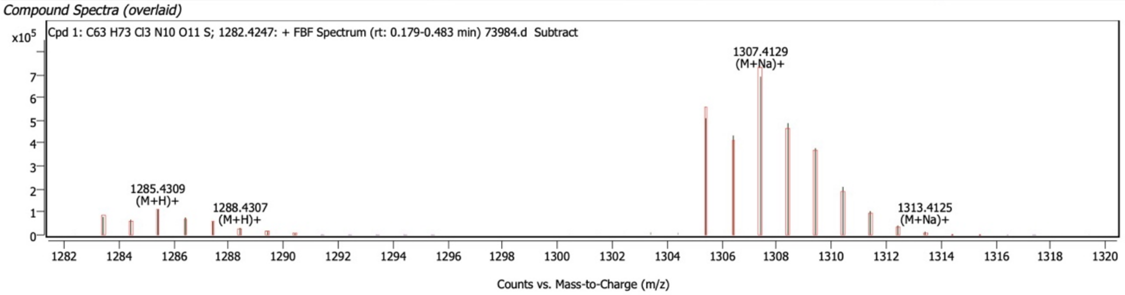

Compound ID Table

Name	Formula	Species	RT	RT Diff	Mass	CAS	ID Source	Score	Score (Lib)	Score (Tgt)
	C63 H73 Cl3 N10 O11 S	(M+H)+ (M+Na)+	0.256		1282.4247		FBF	97.31		97.31

MassHunter Qual 10.0
(End of Report)

Full scans of the blots:

Full scans of Western blots for Figure 2 (main manuscript):

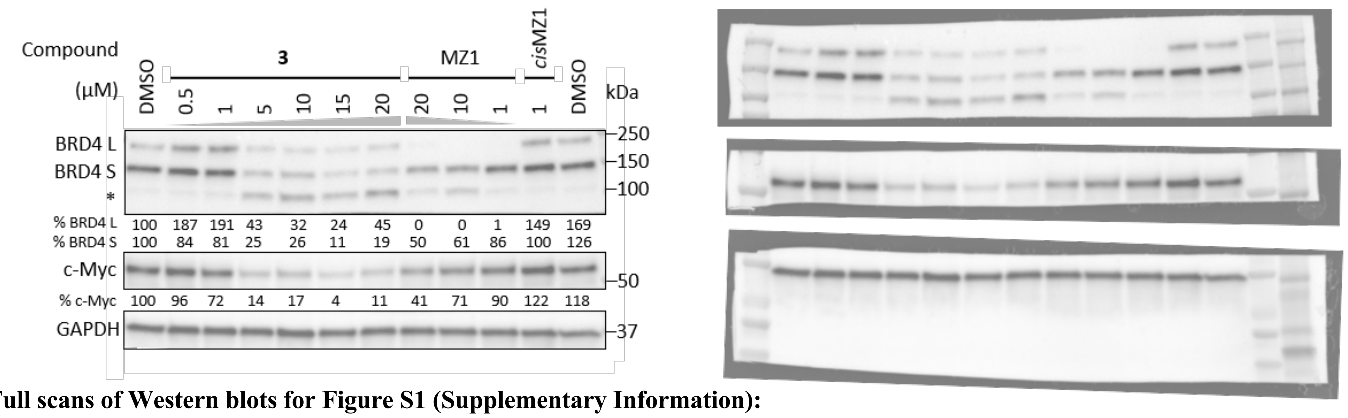

Full scans of Western blots for Figure S1 (Supplementary Information):

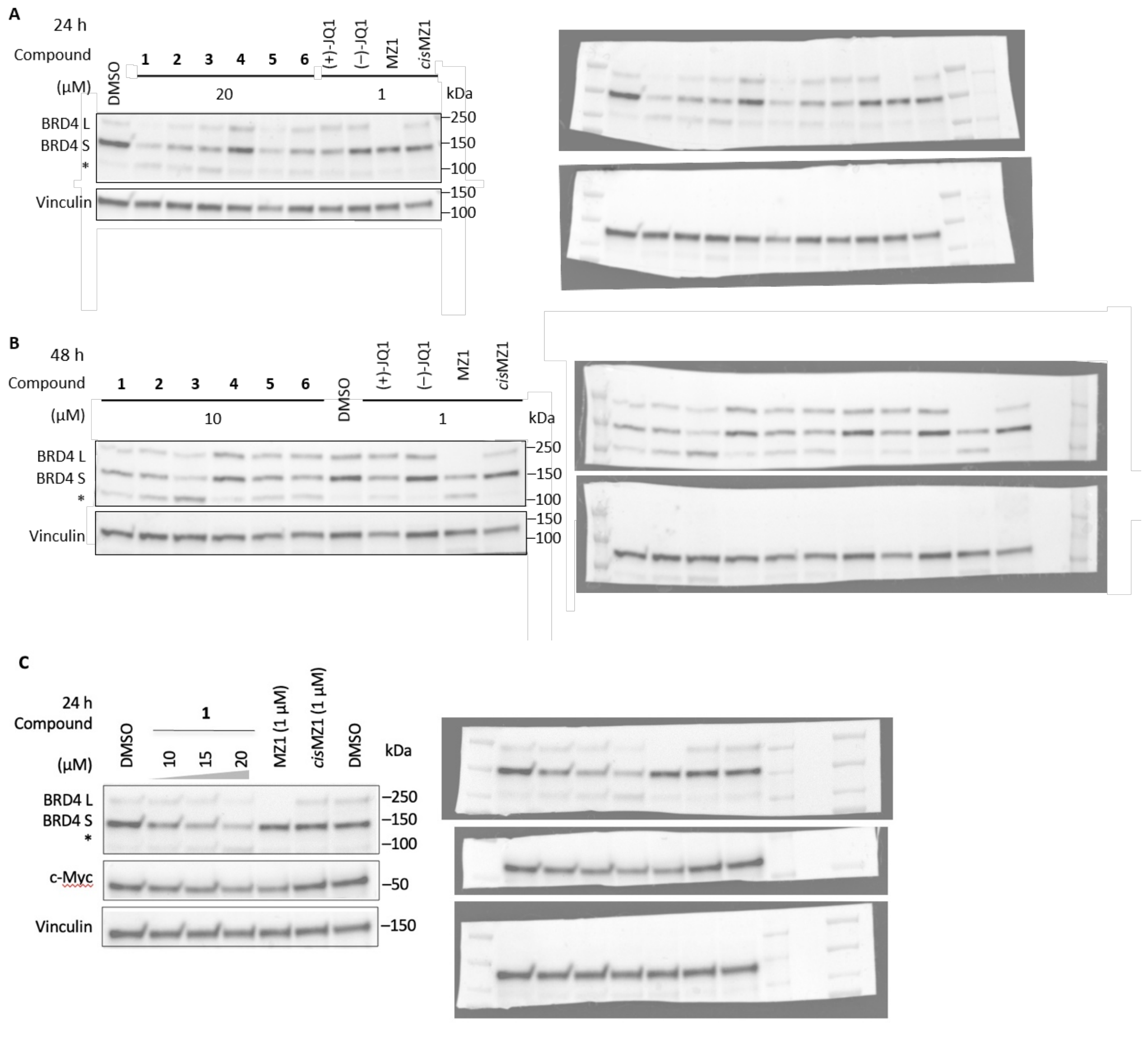

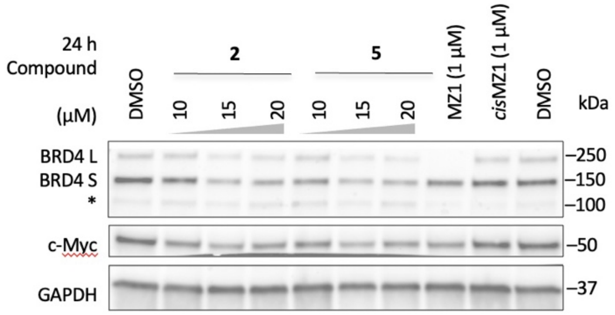

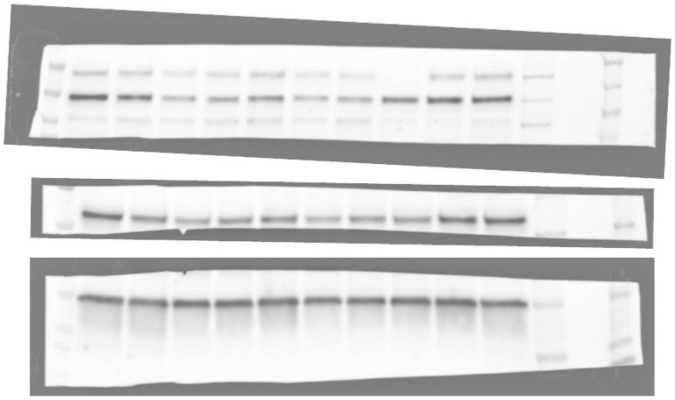

D

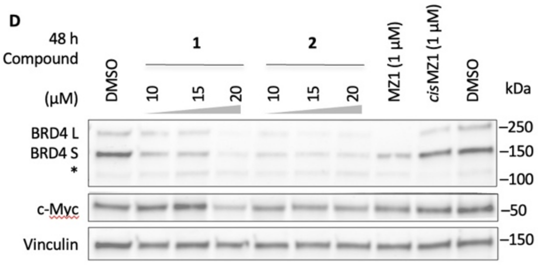

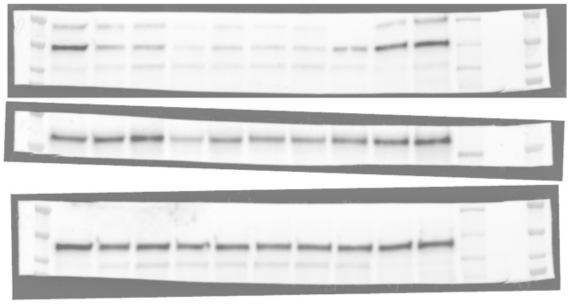

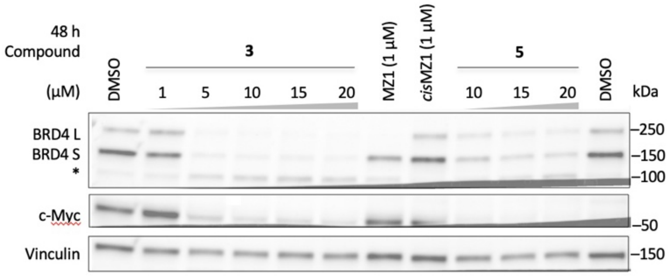

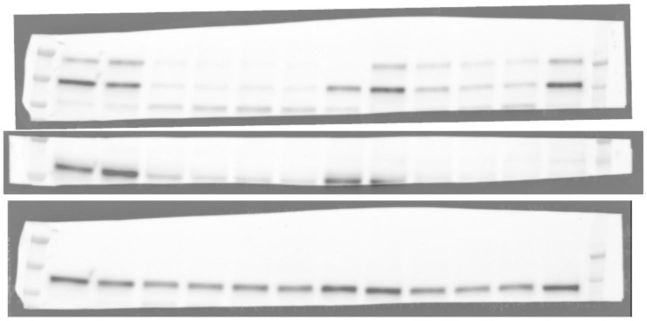

Full scans of Western blots for Figure S2 (Supplementary Information):

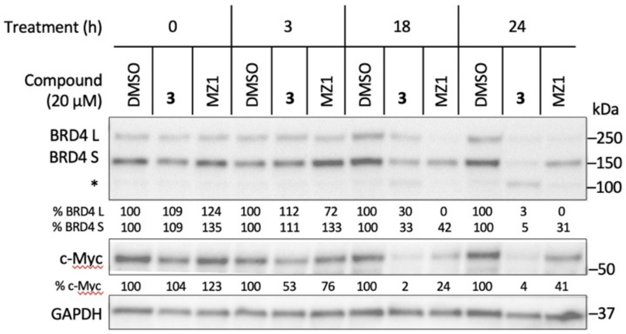

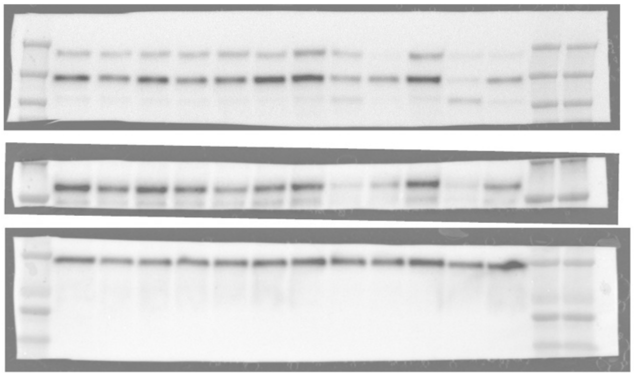

Supplement: Supplementary file 1 — Supplementary Material [file CBIC-26-e202500133-s001.pdf]
